# TAPE-seq is a cell-based method for predicting genome-wide off-target effects of prime editor

**DOI:** 10.1038/s41467-022-35743-y

**Published:** 2022-12-29

**Authors:** Jeonghun Kwon, Minyoung Kim, Seungmin Bae, Anna Jo, Youngho Kim, Jungjoon K. Lee

**Affiliations:** Toolgen, Seoul, Republic of Korea

**Keywords:** Assay systems, Next-generation sequencing, CRISPR-Cas9 genome editing

## Abstract

Prime editors (PEs) are powerful tools that widen the possibilities for sequence modifications during genome editing. Although methods based on the analysis of Cas9 nuclease or nickase activity have been used to predict genome-wide off-target activities of PEs, no tool that directly uses PEs for this purpose has been reported yet. In this study, we present a cell-based assay, named TAgmentation of Prime Editor sequencing (TAPE-seq), that provides genome-wide off-target candidates for PEs. TAPE-seq analyses are successfully performed using many different versions of PEs. The TAPE-seq predictions are compared with results from two other off-site prediction methods, Cas9 nuclease-based GUIDE-seq and Cas9 nickase-based Digenome-seq (nDigenome-seq). TAPE-seq shows a lower miss rate, and a higher area under the receiver operating characteristic curve compared to the other methods. TAPE-seq also identified valid off-target sites that were missed by the other methods.

## Abstract (web summary)

Methods to predict genome-wide off-target activities of prime editors (PEs) are currently lacking. Here the authors report a cell-based assay, TAgmentation of Prime Editor sequencing (TAPE-seq), that provides genome-wide off-target candidates for PEs.

## Introduction

CRISPR-Cas9 can introduce double-strand breaks (DSBs) at off-target as well as on-target sites, and various experimental protocols have been developed to predict such off-target activities at a genome-wide level. The methods can be categorized into three types depending on their mechanism of action: Cell-based (GUIDE-seq^1^, GUIDE-tag^2^, BLISS^3^, BLESS^4^, DISCOVER-seq^5^, integrase-defective lentiviral vector-mediated DNA break capture^6^, HTGTS^7^, CReVIS-Seq^8^, ITR-seq^9^, TAG-seq^10^, and INDUCE-seq^11^), in vitro (e.g., Digenome-seq^12^, DIG-seq^13^, CHANGE-seq^14^, CIRCLE-seq^15^, and SITE-seq^16^), and in silico (e.g., Cas-OFFinder^17^, CRISPOR^18^, and CHOPCHOP^19^). Because each has pros and cons, two or three methods have been used in combination to predict genome-wide off-target activities of CRISPR-based therapeutics^20–22^.

These tools can also be used to predict genome-wide off-target activities of cytidine base editors (CBEs)^23^ and adenine base editors (ABEs)^24^. However, the development of more sophisticated versions of these prediction tools, such as the cell-based methods ONE-seq^25^ and Detect-seq^26^ and the in vitro methods CBE Digenome-seq^27^, ABE Digenome-seq^28^, and EndoV-seq^29^ enabled more direct predictions, because these tools either use the same molecular mechanisms as the base editors or mimic these mechanisms.

Prime editor 2 (PE2) is a versatile genome editing tool that can insert, delete, or substitute nucleotides in target genomic DNA sequences^30^. It consists of a fusion between catalytically impaired Cas9 nickase and an engineered reverse transcriptase (RT) complexed with a prime editing guide RNA (pegRNA), which contains a spacer sequence, a primer binding site (PBS), and a RT template that contains the desired edit. The Cas9 nickase, guided by the spacer sequence in the pegRNA, nicks the non-target DNA strand. The PBS in the pegRNA then binds to the single-stranded DNA released from the nicked strand, the end of which then primes reverse transcription of DNA using the RT template in the pegRNA. The newly synthesized DNA ultimately hybridizes with the uncleaved complementary DNA strand after cleavage of a 5′ flap sequence, which lacks the edit, and is ligated with the nicked DNA strand. The mismatch in the heteroduplex is repaired via cellular repair mechanisms, resulting in the insertion of the RT template sequence at the target locus.

Because the first step of the PE2 mechanism is Cas9 nickase-induced nicking of the non-target DNA strand, it has been expected that the off-target activity of PE2 would resemble that of Cas9 or Cas9 nickase. Therefore, the genome-wide off-target activity of PE2 has been estimated using GUIDE-seq^30^, nDignome-seq^31^, and in silico prediction tools like CAS-OFFinder^17,32^, which measure or predict DSB or nickase activity of Cas9 nuclease or nickase. However, a method that directly measures the off-target activity of PE2 has not been reported. Because Cas9 and PE2 are different enzymes, a new method that directly measures the genome-wide off-target activity of PE is needed.

In this study, we develop a cell-based genome-wide off-target prediction tool for PEs named TAPE-seq, which involves direct analysis of PE activity in live cells. We optimize TAPE-seq by using various versions of PEs that had previously been analyzed using GUIDE-seq and nDigneome-seq, allowing comparisons to be made between the three methods.

## Results

### Optimization of the tagmentation rate

Experimental genome-wide off-target prediction methods can be categorized as either cell-based or in vitro based^33^. Because prime editing is a multi-step process involving many cellular enzymes, including flap endonuclease, exonuclease, and ligase, it is difficult to develop an in vitro-based assay that closely mimics this complex cellular process. On the other hand, most of the cell-based methods introduce tag sequences into on- and off-target loci so that they can be amplified by PCR during a later step. Since PE2 nicks its target without causing a DSB, it is not possible to insert double-stranded oligonucleotides or viral DNA fragments as tags for amplification purposes.

However, PE2 itself has the ability to insert any short sequence into the target site. We, therefore, designed pegRNAs with an additional 34-bp tag sequence between the PBS and RT template sequences. For the tag, we chose the same sequence that is used in GUIDE-seq^1^, because it has been proven to work in cells from many different origins. We also chose PBS and RT template sequences that were used in validation experiments after GUIDE-seq^30^ and nDigenome-seq^31^ were used as prediction tools (Supplementary Data 1).

**Fig. 1:**
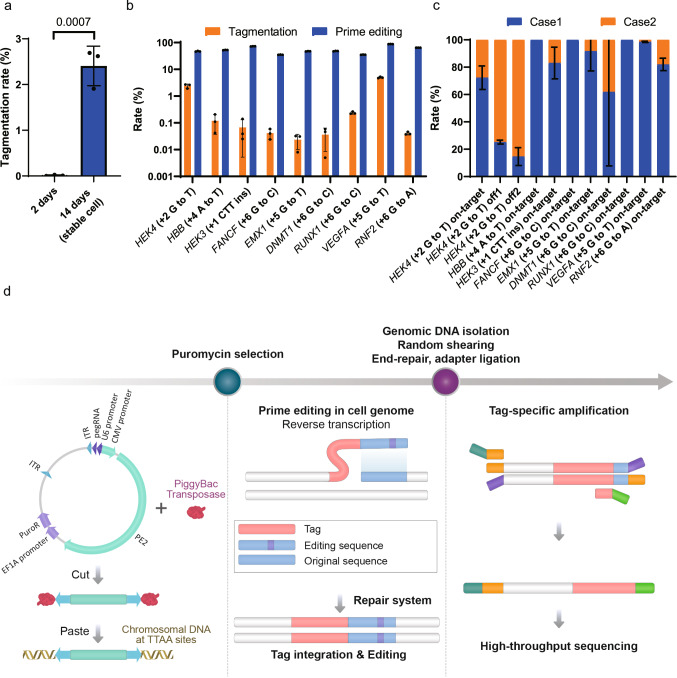
Optimization of the tagmentation rate and analysis of the tagmentation pattern. **a** Tagmentation rates after 2 or 14 days; two-sided unpaired student *t* test. **b** Tagmentation and prime editing rates at nine different on-target sites. **c** Ratios of Case 1 and Case 2 editing determined by targeted deep sequencing and PE Analyzer. **d** Schematic of the TAPE-seq method. The bars represent the mean. Error bars indicate standard deviation (*n* = 3 independent transfections).

The signal-to-noise ratio of the developed off-target prediction method would be proportional to the efficiency of tag insertion at on- and off-target loci. We therefore first optimized the experimental conditions for tag integration into the on-target site. When plasmids encoding PE2 and a *HEK4*-targeting pegRNA (incorporating a + 2 G to T edit, numbered relative to the nick) containing the tag sequence were transiently transfected into HEK293T cells, a tag integration rate of only 0.011% was observed. To improve this rate, we constructed an all-in-one vector encoding PE2 and the pegRNA in the piggyBac system^34^. A stable cell line was constructed via transfection of this vector with transposase; in this situation, the tag integration (tagmentation) rate increased to more than 2% (Fig. 1a) after 14 days of puromycin selection. [Puromycin selection for 14 days successfully enriched green fluorescent protein (GFP) positive cells following transfection with a GFP-piggyBac construct (Supplementary Figure 1)]. The improvements in the number of targets found were not significant even if we prolonged the incubation time from 2 to 7 weeks (Supplementary Figure 2a). [We have assigned a similar number of Miseq reads to the 2 week (5329899), 4 week (5313548), 6 week (2324242), and 7 week (4021702) samples (Supplementary Data 4). The higher number of on-target reads in the 2 week sample (62565) compared to the 4 week (2369), 6 week (1060), and 7 week (1594) samples (Supplementary Data 3) could simply indicate a higher signal-to-noise ratio from the TAPE-seq analysis of the 2 week sample compared to the other samples.] Therefore, puromycin selection was performed for 2 weeks in subsequent studies.

We further optimized the tagmentation rates by finding the optimum amount of piggyBac vector to co-transfect with the transposase plasmid, testing amounts ranging from 50 ng to 1000 ng. When the copy number of the piggyBac vector was measured, 1000 ng resulted in the highest value (Supplementary Figure 2b). In addition, 1000 ng consistently resulted in high tagmentation rates at on-target (Supplementary Figure 2c) and off-target sites (Supplementary Figure 2d). Therefore, we transfected 1000 ng of piggyBac vector in subsequent TAPE-seq experiments.

Next, we tested various lengths of the probe sequence, ranging from 19 to 34 bp, as it is possible that a shorter probe sequence could result in a higher tagmentation rate. Indeed, for the on-target site of the *HEK4* (+2 G to T) pegRNA, a 19-bp probe sequence resulted in higher integration rates compared to a 34-bp probe sequence (Supplementary Figure 2e–j). However, for one of the off-target sites, the opposite trend was observed (Supplementary Figure 2h–j). We chose to use a 34-bp sequence for subsequent analyses, because the objective of TAPE-seq is the tagmentation of off-target sites and because the GUIDE-seq experiment and analysis was optimized using a 34-bp tag sequence. [Both GUIDE-seq^1,35^ and its predecessor, the anchored multiplex PCR (AMP)^36^ method, involve a nested PCR step to ensure high specificity, which is achieved by using two unidirectional primers. When primers were optimized for 17 different targets for AMP analysis^37^, the use of the two tandem primers yielded target priming sites ranging from 35 bp to 71 bp in length, with an average of 46 bp and a median of 44 bp. We reasoned that the reduction in length of the target priming site from 34 bp to 19 bp would eliminate the high specificity obtained with the nested PCR step in GUIDE-seq and the AMP method. Indeed, when the length of the probe sequence was reduced from 34 bp to 19 bp, the number of Nucleotide BLAST^38,39^ hits surged from 1 to approximately 4000, suggesting a 4000 times higher chance of genome-wide mis-priming, which would result in a lower signal-to-noise ratio. Because the 34-bp probe sequence used in GUIDE-seq successfully tagged on-target and off-target sites on six different occasions, we chose to use the 34-bp sequence for subsequent analyses.]

When the tagmentation rates were measured for samples incubated under optimized conditions, each with nine different pegRNAs that contained a tag sequence and that targeted different genes, tagmentations were observed at all of the targets (Fig. 1b). The tagmentation efficiencies were not directly proportional to the PE2 efficiencies, which were measured from the stable cell lines expressing PE2 and the corresponding pegRNAs without the tag sequences. We also compared the tagmentation rates of one on-target and five off-target loci that had previously been identified by nDigenome-seq (Supplementary Figure 3a). Because one of the off-target loci showed a ~100% tagmentation rate, we proceeded to the next step with the aforementioned conditions for the tagmentation step.

### Analysis of on-target and off-target tagmentation patterns

Next, we compared the prime editing pattern at the on-target loci for each prime-edited sample obtained using pegRNAs with the tag sequence. The addition of the tag sequence to the pegRNA results in two alternative integration scenarios. In the first case (Case 1), the 34-bp tag sequence is added without perturbing the rest of the prime editing pattern, such that if the 34-bp probe sequence is removed from this pattern, it is identical to that induced by the pegRNA without the tag. In the second case (Case 2), the tag integration perturbs the prime editing pattern, such that if the 34-bp tag is removed, it is different than that induced by the pegRNA without the tag. When the tag integration patterns at on- and off-target loci for nine different pegRNAs were analyzed with targeted deep sequencing analysis and PE-Analyzer^40^, the majority of the tagmented samples corresponded to the Case 1 scenario (Fig. 1c). In addition, further analysis of Case 1 samples revealed that most of them included both the tag and the prime editing; only a small fraction was tagmented without prime editing (Supplementary Figure 3b, Supplementary Data 2). From these results, we concluded that the presence of the tag sequence has minimal effect on the prime editing pattern at on- and off-target sites.

### Analysis of tagmented genomic DNA to predict the genome-wide off-target effects of PE2

We purified the tagmented genomic DNA and processed it using the protocol from GUIDE-seq^1,35^ for tag-specific amplification to produce a TAPE-seq library. In the previous analysis^31^, *HEK4*-targeted pegRNAs were associated with a large number of validated off-target sites compared to pegRNAs targeting other sites. We, therefore, optimized the TAPE-seq protocol using the *HEK4* site as a case study. First, we analyzed the TAPE-seq library made from the same genomic DNA pool, produced after cells were transfected with plasmids encoding PE2 and the *HEK4* (+2 G to T) pegRNA, with MiSeq and HiSeq, and summarized the results in a Venn diagram (Supplementary Figure 4a). HiSeq (53,771,178 reads) did not reveal more candidate off-target sites, indicating that the read number for MiSeq (2,251,379 reads) is large enough for this analysis. However, the HiSeq and MiSeq results each missed some of the other’s predicted off-target sites even when the TAPE-seq library made from the same genomic DNA sample was used. We speculate that due to low tagmentation efficiencies at off-target sites, the tag-specific amplifications of some of these off-target sites were not replicated in each run. We also compared the TAPE-seq results for the *HEK4* (+2 G to T) and *HEK4* (+3 ATT ins) pegRNAs, which were also previously analyzed. The results, summarized in a Venn diagram, show that TAPE-seq analysis of the *HEK4* (+2 G to T) pegRNA-treated sample correctly predicted a validated off-target site of the *HEK4* (+3 ATT ins) pegRNA, which was missed by TAPE-seq analysis for the *HEK4* (+3 ATT ins) pegRNA (Supplementary Figure 4b), and vice versa (Supplementary Figure 4c). We speculate that the off-target profile of the *HEK4* (+2 G to T) pegRNA is similar to that of the *HEK4* (+3 ATT ins) pegRNA, so the difference between the TAPE-seq results for these two samples may be caused by the same replication issue found for the HiSeq and MiSeq samples following treatment with the *HEK4* (+2 G to T) pegRNA: the low tagmentation rate of off-target sites. We, therefore, combined all three sets of TAPE-seq results for the *HEK4* pegRNA for later analysis and labeled the combined results *HEK4* [combined] for simplicity.

### Comparisons of TAPE-seq prediction results with those from GUIDE-seq and nDigenome-seq

**Fig. 2:**
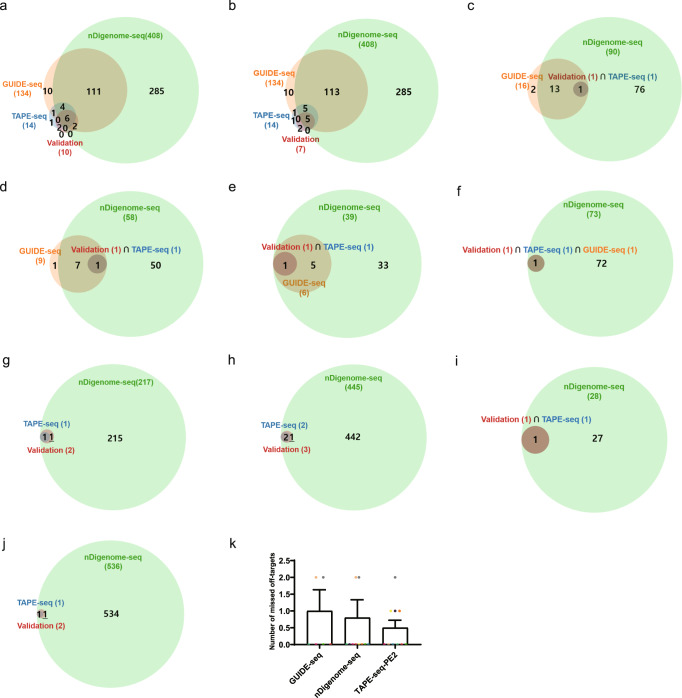
Comparisons of TAPE-seq prediction results with those from GUIDE-seq and nDigenome-seq. **a**–**j** Venn diagrams showing the number of and overlap between off-target loci predicted by nDigenome-seq, GUIDE-seq, and TAPE-seq and validated off-target sites for **a** *HEK4* (+2 G to T), **b** *HEK4* (+3 TAA ins), **c** *EMX1* (+5 G to T), **d** *FANCF* (+6 G to C), **e** *HEK3* (+1 CTT ins), **f** *RNF2* (+6 G to A), **g** *DNMT1* (+6 G to C), **h** *HBB* (+4 A to T), **i** *RUNX1* (+6 G to C), and **j** *VEGFA* (+5 G to T) pegRNAs. **k** The number of off-target sites missed by nDigenome-seq (*n* = 10 independent experiments each represented by different color), GUIDE-seq (*n* = 6 independent experiments each represented by different color), and TAPE-seq (*n* = 10 independent experiments each represented by different color). Some numbers have been underlined to differentiate neighboring numbers (**g**, **h**, **j**). The bars represent the mean. Error bars indicate standard deviation (**k**).

TAPE-seq analyses were performed with the optimized protocol for ten different pegRNAs (Supplementary Data 3 and 4) and compared with the previous predictions made by GUIDE-seq and nDigenome-seq. Validation experiments were performed for all of the off-target candidates (referred to herein as off1, off2, etc.) predicted by TAPE-seq using a HEK293T cell line that stably expressed PE2 and the appropriate pegRNA (Supplementary Data 5). Some of the targets identified by nDigenome-seq that were determined to be false positives were validated in our experiment (Supplementary Data 6). This result may be due to the prolonged incubation period in our protocol (4 weeks) compared to the transient transfection used in the nDigenome-seq validation experiments (96 h). We also performed validation experiments for the validated target loci identified by the methods from previous papers even if they were missed by TAPE-seq. Venn diagrams showing the overlap of off-target sites predicted by TAPE-seq, GUIDE-seq, and nDigenome-seq and validated loci were constructed (Fig. 2a–j). TAPE-seq predicted far fewer off-target sites than GUIDE-seq and nDigneome-seq. However, TAPE-seq also missed fewer off-target sites than either of the other methods (Fig. 2k), suggesting that TAPE-seq predictions show high accuracy.

### TAPE-seq analysis using PE2 and PE4 in different cell lines

**Fig. 3:**
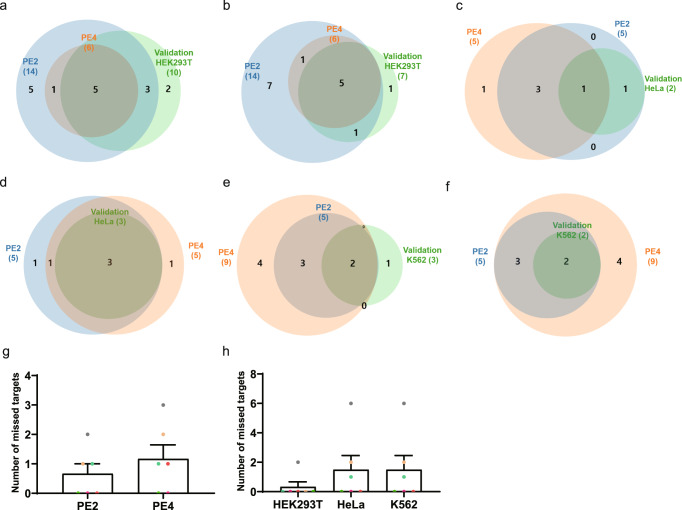
TAPE-seq analysis using PE2 and PE4 in different cell lines. **a**–**f** Venn diagrams showing the number of and overlap between validated off-target sites in PE2-transfected cell lines and the off-target sites predicted by TAPE-seq using PE2 or PE4 with the following pegRNAs and cell lines: **a** *HEK4* (+2 G to T) in HEK293T cells, **b** *HEK4* (+3 TAA ins) in HEK293T cells, **c** *HEK4* (+2 G to T) in HeLa cells, **d** *HEK4* (+3 TAA ins) in HeLa cells, **e** *HEK4* (+2 G to T) in K562 cells, and **f** *HEK4* (+3 TAA ins) in K562 cells. **g** The number of off-target sites missed by the different versions of PE (PE2 or PE4) used for TAPE-seq. **h** The number of missed off-target sites in the different cell lines (HEK293T, HeLa, and K562) used for TAPE-seq. The bars represent the mean. Error bars indicate standard deviation (*n* = 6 independent experiments each represented by different color).

Later versions of PEs have been developed and have been reported to show higher prime editing efficiencies than earlier versions. We reasoned that TAPE-seq could be further optimized by using Prime Editor 4 (PE4), which is a modified version of PE2 that exhibits higher prime editing efficiencies due to the inclusion of a plasmid that encodes dominant negative MLH1 to inhibit mismatch repair^41^. It is possible that the higher efficiency of PE4 would also lead to a higher number of off-target candidates compared to that seen with PE2. In addition, we wanted to check whether performing TAPE-seq in different cell lines would produce better predictions. To this end, we performed TAPE-seq using PE2 and PE4 in HEK293T, HeLa, and K562 cells (Supplementary Data 3). No significant differences were seen in the tagmentation rates at the on-target and one of the off-target loci of the *HEK4* (+2G to T) pegRNA in the three cell lines (Supplementary Figure 5a, b). We validated the predicted off-target locus via targeted deep sequencing. Venn diagrams show that PE4 missed more validated off-target sites than PE2 (Fig. 3a–f, g). In addition, TAPE-seq performed in HEK293T cells missed fewer validated off-target sites compared to analysis in the other two cell lines (Fig. 3h).

Next, we determined whether the candidate off-target sites in the HEK293T, HeLa, and K562 cell lines could be validated and compared the validation results with the TAPE-seq predictions for the respective cell lines using Venn diagrams (Supplementary Figure 5c–h). Far fewer validated off-target sites were found in HeLa and K562 cells compared to HEK293T cells; furthermore, only a few off-target sites were missed by TAPE-seq in each cell line (Supplementary Figure 5i). We speculate that the TAPE-seq predictions in each cell line are accurate. In addition, TAPE-seq predictions made using the HEK293T cell line identified all valid off-target sites for HeLa and K562 cells. Therefore, we excluded PE4 and used HEK293T cells for all subsequent experiments.

### TAPE-seq analysis using PE2-nuclease and PEmax-nuclease with engineered pegRNAs

Prime editor nucleases, which contain wild-type Cas9 nuclease instead of Cas9 nickase, have also been reported to exhibit higher prime editing efficiencies than PE2^42^. However, these PEs also result in a higher indel ratio as a side effect. We reasoned that the use of these prime editor nucleases would result in higher tagmentation rates at off-target loci, increasing the success rate of TAPE-seq for identifying novel off-target loci.

**Fig. 4:**
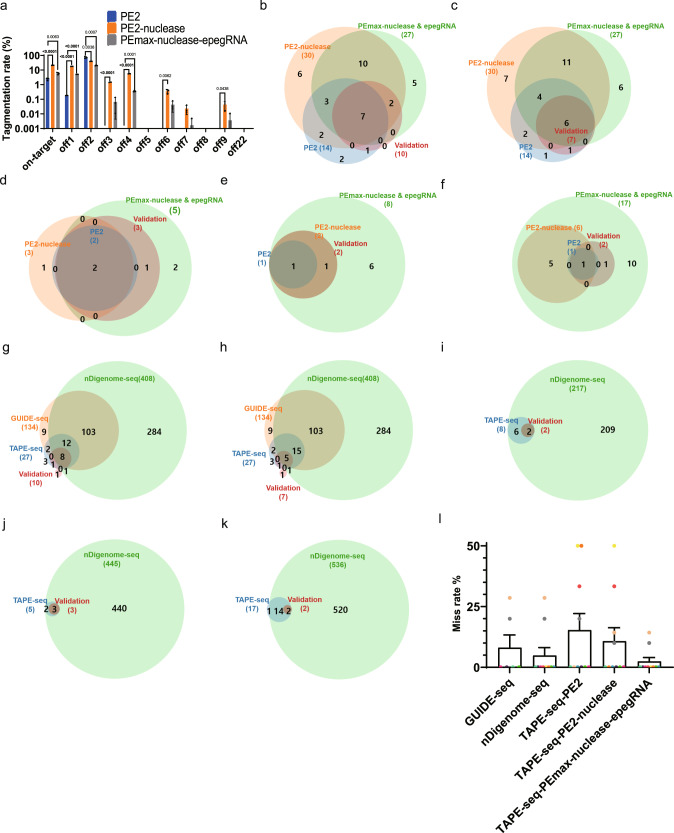
TAPE-seq analysis using PE2-nuclease and PEmax-nuclease with engineered pegRNAs. **a** Tagmentation rates at the *HEK4* on-target site and various associated off-target sites after TAPE-seq using PE2, PE2-nuclease, or PEmax-nuclease with epegRNAs was performed; two-sided unpaired student *t* test. (*n* = 3 independent transfection). **b**–**f** Venn diagrams showing the number of and overlap between off-target loci predicted by TAPE-seq using PE2, PE2-nuclease, or PEmax-nuclease with epegRNAs, and validated off-target sites for the **b** *HEK4* (+2 G to T), **c** *HEK4* (+3 TAA ins), **d** *HBB* (+4 A to T), **e** *DNMT1* (+6 G to C), and **f** *VEGFA* (+5 G to T) pegRNAs. **g**–**k** Venn diagrams showing the number of and overlap between off-target loci predicted by TAPE-seq using PEmax-nuclease with epegRNAs, GUIDE-seq, and nDigenome-seq and validated off-target sites for the **g** *HEK4* (+2 G to T), **h** *HEK4* (+3 TAA ins), **i** *HBB* (+4 A to T), **j** *DNMT1* (+6 G to C), and **k** *VEGFA* (+5 G to T) pegRNAs. **l** Miss rates of the five different off-target prediction methods (*n* = 6 independent experiments for GUIDE-seq and *n* = 10 independent experiments for the rest of the methods each represented by different color). The bars represent the mean. Error bars indicate standard deviation.

Optimization experiments showed that transient transfections were sufficient for PE2-nuclease^42^ and PEmax-nuclease with engineered pegRNAs (epegRNAs)^41,43^, confirmed by the high tagmentation rates compared to that found with TAPE-seq performed with PE2 (Fig. 4a). [Although the on-target tagmentation rates of PE2-nuclease and PEmax-nuclease with epegRNAs were significantly higher than that of PE2 (Fig. 4a), there were only 1110 on-target TAPE-seq reads for PE2-nuclease and 906 for PEmax-nuclease with epegRNAs, compared to 62565 for the PE2 (2 week) sample (Supplementary Data 3). Nevertheless, PE2-nuclease and PEmax-nuclease with epegRNAs led to the identification of 30 and 27 candidates, respectively, compared to 8 candidates identified in the PE2 (2-week) sample.]

We undertook TAPE-seq with ten different pegRNAs using PE2-nuclease and PEmax-nuclease with epegRNAs and compared the results with that of TAPE-seq using PE2 with Venn diagrams (Fig. 4b–f, Supplementary Figure 6a–e). Venn diagrams were also used to compare predictions from TAPE-seq using PEmax-nuclease with epegRNAs with those from GUIDE-seq and nDigenome-seq (Fig. 4g–k, Supplementary Figure 6g–j). To summarize the results, we compared the miss rates (defined as the number of missed validated off-target sites divided by the total number of validated off-target sites) of TAPE-seq performed using PE2, PE2-nuclease, and PEmax-nuclease with epegRNAs to those of GUIDE-seq and nDigenome-seq for ten different pegRNAs (Supplementary Data 7, Fig. 4l). TAPE-seq using PEmax-nuclease with epegRNAs showed the lowest miss rate. It should be noted that the number of missed validated off-target sites for PE2 has increased compared to the results shown in Fig. 2k, because TAPE-seqs performed using PE2-nuclease and PEmax-nuclease with epegRNAs have identified novel validated off-target sites.

### TAPE-seq analysis using PEmax-nuclease with epegRNAs shows the highest area under the receiver operating characteristic (ROC) curve

**Fig. 5:**
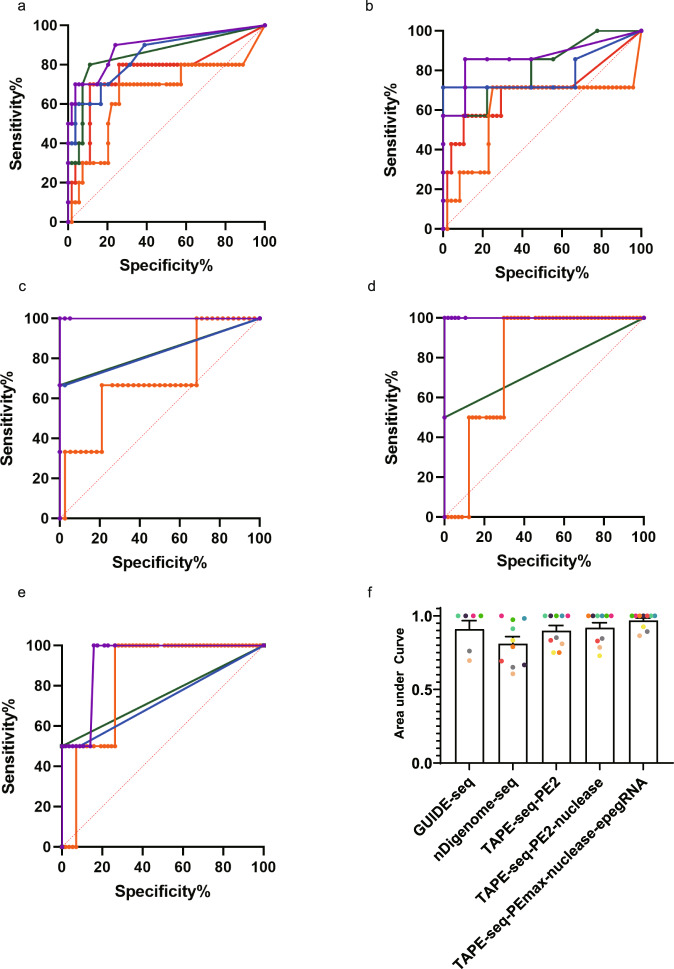
ROC curve analysis. **a**–**e** ROC curves for GUIDE-seq (red), nDigenome-seq (orange), and TAPE-seq using PE2 (green), PE2-nuclease (blue), and PEmax-nuclease with epegRNAs (purple) for the **a** *HEK4* (+2 G to T), **b** *HEK4* (+3 TAA ins), **c** *HBB* (+4 A to T), **d** *DNMT1* (+6 G to C), and **e** *HEK3* (+1 CTT ins) pegRNAs. **f** Area under ROC curves (*n* = 6 independent experiments for GUIDE-seq and *n* = 10 independent experiments for the rest of the methods each represented by different color). The bars represent the mean. Error bars indicate standard deviation.

A ROC curve is a plot that shows the diagnostic ability of a binary classifier. We reasoned that by constructing ROC curves for TAPE-seq analyses using PE2, PE2-nuclease, and PEmax-nuclease with epegRNAs to compare with those for GUIDE-seq and nDigenome-seq, we could quantitatively compare the diagnostic ability of TAPE-seq’s metric (copy number) with that of GUIDE-seq (copy number) and nDignome-seq (DNA cleavage score). When the area under the ROC curves were compared to each other (Fig. 5a–e, Supplementary Figure 7a–e), TAPE-seq using PEmax-nuclease with epegRNAs showed the highest value (Fig. 5f). This result suggests that the TAPE-seq metric shows superior diagnostic ability when compared to that of GUIDE-seq and nDigenome-seq in predicting off-target sites.

### Editing patterns at validated off-target sites

**Fig. 6:**
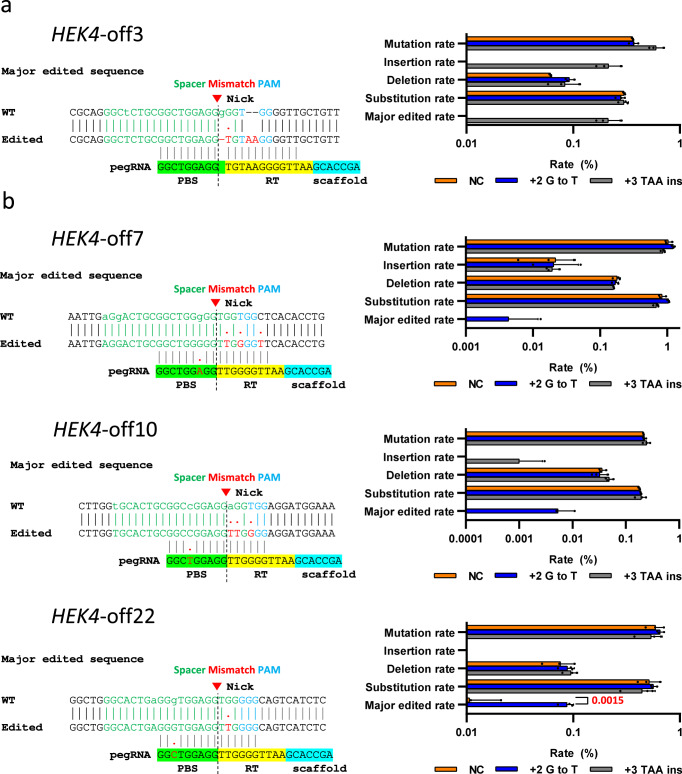
Off-target prime editing patterns. **a** Editing patterns induced by the *HEK4* (+3 TAA ins) pegRNA at the *HEK4*-off3 site predicted by TAPE-seq. **b** Editing patterns induced by the *HEK4* (+2 G to T) pegRNA at the *HEK*-off7, *HEK*-off10, and *HEK*-off22 sites predicted by TAPE-seq; two-sided unpaired student *t* test. NC negative control. The bars represent the mean. Error bars indicate standard deviation (*n* = 3 independent transfection). Small letters indicate mismatches compared to the pegRNA. Major edited rate corresponds to the frequency of the ‘Edited’ sequence.

The editing patterns at all of the validated off-target sites were analyzed by comparing targeted deep sequencing results for the *HEK4* (+2 G to T), *HEK4* (+3 TAA ins), *HBB* (+4 A to T), *DNMT1* (+6 G to C), and *VEGFA* (+5 G to T) pegRNAs used with PE2, PE2-nuclease, and PEmax-nuclease with epegRNAs in HEK293T, HeLa, and K562 cells (Supplementary Data 9). At the *HEK4*-off3 site predicted by TAPE-seq, only the *HEK4* (+3 TAA ins) pegRNA induced editing (Fig. 6a), whereas for the off-target sites *HEK4-*off7, *HEK4-*off10, and *HEK4-*off22 predicted by TAPE-seq, only the *HEK4* (+2 G to T) pegRNA gave rise to off-target effects (Fig. 6b). These results suggest that off-target effects are also dependent on the RT template sequence. This phenomenon may partly explain the higher area under ROC curve for TAPE-seq compared to GUIDE-seq or nDigenome-seq, as these two methods are performed with single guide RNAs lacking a RT template sequence.

### Mismatch analysis by region

**Fig. 7:**
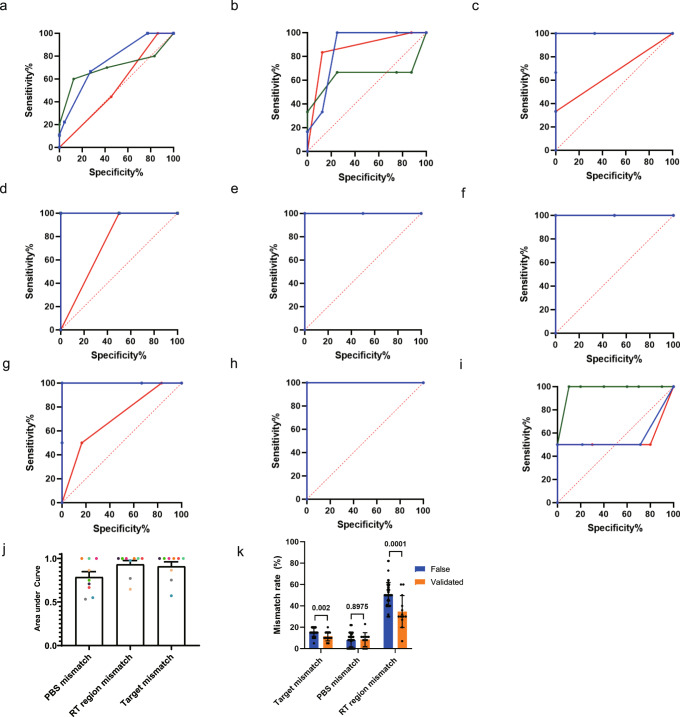
Mismatch analysis by region. **a**–**i** ROC curves for mismatches in the PBS (red), RT template (green), and target (blue) regions for the **a** *HEK4* (+2 G to T), **b** *HEK4* (+3 TAA ins), **c** *HBB* (+4 A to T), **d** *HEK3* (+1 CTT ins), **e** *FANCF* (+6 G to C), **f** *EMX1* (+5 G to T), **g** *DNMT1* (+6 G to C), **h** *RUNX1* (+6 G to C), and **i** *VEGFA* (+5G to T) pegRNAs. **j** Area under the ROC curve for the nine different pegRNAs (*n* = 10 independent experiments each represented by different color). **k** Mismatch rates for sites predicted by TAPE-seq, false positive sites (*n* = 54 independent target loci for Target mismatch and RT region mismatch and *n* = 47 target loci for PBS mismatch; 7 loci with DNA/RNA bulge for PBS mismatch were excluded from the analysis), and validated sites (*n* = 13 independent target loci for Target mismatch and RT region mismatch and *n* = 12 target loci for PBS mismatch; 1 locus with DNA/RNA bulge for PBS mismatch was excluded from the analysis); two-sided unpaired student *t* test. The bars represent the mean. Error bars indicate standard deviation.

Next, we tabulated mismatch numbers in the PBS, RT template, and spacer regions in the pegRNAs for the on-target and off-target sites and listed them together with validation results (Supplementary Data 8). ROC curves were constructed using the number of mismatches instead of the copy number as the metric to predict validation results as a binary classification (Fig. 7a–i; *RNF2* was excluded as it had only one sample). In most cases, the area under the ROC curve for mismatches in the RT template region was higher than that for mismatches in the PBS and target regions (Fig. 7j). In addition, when the mismatch rates of false and validated targets in the PBS, target, and RT template regions were compared, rates for false were significantly higher than that for validated in the target and RT template regions, not in the PBS region (Fig. 7k). All in all, RT template mismatches seem to show as much diagnostic ability as target mismatches for predicting the validity of potential off-target sites. Unlike TAPE-seq, GUIDE-seq and nDigenome-seq do not involve RT in their protocols, limiting their ability to accommodate the molecular mechanism of RT in their off-target prediction processes. We speculate that TAPE-seq’s higher diagnostic ability originates from its recruitment of RT and subsequent elimination of false positive off-target sites.

## Discussion

In this paper, we describe the development of TAPE-seq, which shows high predictive power for genome-wide off-target effects of PE2. Similar to results described in the previous papers^1–3^, TAPE-seq also identified fewer off-target loci for PE2 compared to those associated with DSB-inducing Cas9 targeted to the same sites. Recently, various techniques have been developed^41,42,44–47^ to increase the efficiency of PE2. Some of these techniques^41–43^ have been applied to the TAPE-seq protocol to increase the tagmentation rate, which also increased the sensitivity of TAPE-seq for identifying novel off-target loci that were missed by previous methods. It is anticipated that increasing the sensitivity of TAPE-seq will result in the identification of more, previously missed, off-target loci. In addition, the tagmentation condition could also be further optimized to increase the sensitivity of TAPE-seq.

The potential advantages of TAPE-seq include that it is an unbiased cell-based method that can detect cell type-specific prime editing events with high validation rates, low miss rates, and high areas under the ROC curve. This method directly measures PE genome editing activities by accommodating the RT mechanism, unlike other methods such as nDigenome-seq and GUIDE-seq that only give indirect measures of the nickase or DSB activities of Cas9. In addition, whereas the biggest limitation of GUIDE-seq is the necessity for transfecting a double-stranded oligodeoxynucleotide (dsODN) tag, which could be toxic to some intolerant cells or not possible in an animal model, the TAPE-seq tag sequence is included in the pegRNA itself, so that toxicities due to dsODNs are irrelevant; furthermore, in vivo delivery of TAPE-seq vectors is also possible.

There are several potential limitations of TAPE-seq. First, performing TAPE-seq in a ‘surrogate’ cell line to predict off-target loci in other cell types could result in a high off-target prediction miss rate due to cell type-specific activities. Additionally, because the pegRNA is single-stranded, the tag sequence could form a secondary structure with the neighboring RT or PBS sequence. Such an occurrence could be detected by a low on-target tagmentation rate, in which case the tag sequence should be modified before the definitive TAPE-seq analysis is performed; the reverse complementary sequence of 34-bp tag sequence could be used or another tag sequence that does not form the secondary structure could be designed, a process that could be assisted by prediction tools such as Vienna2.0^48^, which is used to engineer pegRNA designs^43^.

For recently developed CRISPR-based therapeutics like EDIT-101^21^ and NTLA-2001^20^, off-target prediction results from cell-based, in vitro, and in silico methods were combined for the Initiation of New Drug (IND) applications. We expect that as more PE-based therapeutics are developed^44,49–52^, TAPE-seq will become one of the powerful cell-based methods for studying the safety of PE-based drugs before clinical trials.

## Methods

### Plasmid construction

The sgRNA-expressing plasmid pRG2 (addgene #104174) was modified to create a pegRNA-expressing plasmid (pRG2-pegRNA) by Gibson assembly following cleavage at the BsmBI restriction site at the 3′ terminus of the sgRNA scaffold. The plasmid was modified to contain a BsaI site (for incorporation of a spacer sequence) and a BsmBI site (for incorporation of the pegRNA 3′ extension). To create the piggyBac PE2 all-in-one plasmid (pAllin1-PE2), the piggyBac PE2-expressing plasmid DNA was synthesized and cloned to make a vector (piggy-PE2). It was then digested with Mlu I. The pegRNA-encoding sequence was amplified from pRG2-pegRNA by PCR to generate the insert fragment, which was cloned into the digested piggyBac PE2 vector via Gibson assembly. Other PE all-in-one plasmids (pAllin1-PE4, pAllin1-PE2-nuclease, and pAllin1-PEmax-nuclease) were constructed using the same procedure that was used to construct pAllin1-PE2. The pRG2-epegRNA vector was constructed using the same procedure that was used to construct pRG2-pegRNA. The DNA sequences of all of the constructed vectors (pRG2-pegRNA, pAllin1-PE2, piggy-PE2, pRG2-epegRNA, pAllin1-PE4, pAllin1-PE2-nuclease, and pAllin1-PEmax-nuclease) are available in Supplementary Data 10.

### Human cell culture and transfection

HEK293T (ATCC CRL-1268), HeLa (ATCC CCL-2), and K562 (Sigma 89121407) cells were maintained in the appropriate medium [Dulbecco’s Modified Eagle Medium (DMEM) for HEK293T and HeLa cell lines, Roswell Park Memorial Institute 1640 Medium (RPMI 1640) for the K562 cell line] containing 10% fetal bovine serum (FBS) and 1% penicillin-streptomycin at 37°C in the presence of 5% CO_2_. 1 × 10^5^ HEK293T cells or 4 × 10^4^ HeLa cells were seeded in a 24-well plate in preparation for transfection. One day after seeding, cells were transfected with an adequate amount of plasmid (see below) and 2 μl Lipofectamine 2000 (Thermo Fisher Scientific). [For transient PE2 expression, 500 ng piggy-PE2 and 500 ng pRG2-pegRNA; for stable PE2 expression, 850 ng pAllin1-PE2 and 150 ng piggyBac Transposase Expression Vector (System Biosciences); for stable PE4 expression, 880 ng pAllin1-PE4 and 120 ng piggyBac Transposase Expression Vector; for stable PE2-EGFP expression, 865 ng pAllin1-PE2-EGFP and 135 ng piggyBac Transposase Expression Vector; for transient PE2-nuclease expression, 1000 ng pAllin1-PE2-nuclease; and for transient PEmax-nuclease and epegRNA expression, 1000 ng pAllin1-PEmax-nuclease-epegRNA were used.] The transposon and piggybac plasmids were used at about a 2.5:1 transposon:transposase plasmid molar ratio. 1 × 10^5^ K562 cells were electroporated with the above-mentioned quantities of plasmid via a Neon transfection system (electroporation conditions: 1450 V, 10 ms, 3 pulses). One day after transfection (or electroporation), antibiotic selection was conducted using puromycin (InvivoGen) at a concentration of 2 mg/ml. Puromycin selection was continued for 2 weeks [for TAPE-seq and fluorescence-activated cell sorting (FACs)], 4 weeks (for targeted deep sequencing), or 2 days (for TAPE-seq using PE2-nuclease or PEmax-nuclease; after puromycin selection, cells were cultured for an additional 4 days in normal media). Genomic DNA was purified with a Blood Genomic DNA Extraction Mini Kit (Favorgen) following the manufacturer’s instructions.

### TAPE-seq

A full description of the TAPE-seq method can be found in Supplementary Note 1. Genomic DNA was sheared with a Covaris M220 instrument to an average length of 325 bp and isolated with 1× AMPure XP beads (Beckman coulter). Using an NEBNext® Ultra™ II DNA Library Prep Kit for Illumina (NEB), a next-generation sequencing (NGS) library was prepared according to the manufacturer’s protocol, with slight modifications to certain reaction times (adaptor ligation, 1 h; treatment with Uracil-Specific Excision Reagent, 30 min). Using tag- and adaptor-specific primers, tag-specific library amplification was performed according to previously described GUIDE-seq methods^1,2^. The amplified library was analyzed with a MiSeq or HiSeq platform (Illumina).

Paired-end FASTQ files were processed using the following steps: 1. Sequences including the tag were collected using the BBDuk program (Tag sequences for sense library(+), 5′-GTTTAATTGAGTTGTCATATGT-3′ and 5′-ACATATGACAACTCAATTAAAC; Tag sequences for antisense library(-), 5′-TTGAGTTGTCATATGTTAATAACGGTA-3′ and 5′-TACCGTTATTAACATATGACAACTCAA-3′). 2. Filtered FASTQ files were mapped to the reference genome (hg19) and the read depth was calculated using BWA, Picard tools, and SAMtools programs. 3. Off-target candidates (containing up to 4 mismatches and/or 2 bulges relative to the on-target site) were identified using Cas-OFFinder^3^ (http://www.rgenome.net). 4. The read depths at the sites identified by Cas-OFFinder were extracted from the region spanning −150 bp to +150 bp around the site using an in-house script. 5. Short-mapped sequences (less than 30 bp in length) and false tagmentation sequences (in which tagmentation occurred outside of the PE nick site) were excluded.

### Targeted deep sequencing and validation of off-target sites

Following expression of PE2 and the pegRNA, target sites were analyzed by targeted deep sequencing. Deep sequencing libraries were generated by PCR. TruSeq HT Dual Index primers were used to label each sample. Pooled libraries were subjected to paired-end sequencing using MiSeq (Illumina). Paired-end FASTQ files were analyzed with PE-Analyzer (http://www.rgenome.net). Candidates that satisfied the following two conditions were designated as ‘validated off-targets’: 1. The frequency of at least one of the following events (mutation, insertion, deletion, substitution, or major editing) was higher than that in the wild-type sample. 2. A mutated sequence that could only be generated by prime editing (the major edited sequence) was present. To overcome the detection limits of NGS and problems created by PCR error, validation experiments were conducted using cells in which PE2 had been stably expressed for 4 weeks, and were performed in triplicate using biologically independent genomic DNA. The validation rate was calculated by dividing the number of validated targets by the sum of (the number of validated targets + the number of false positive targets). Targets that were not analyzed were excluded from the validation rate calculation.

### Prime editing tagmentation analysis

The presence of the tag sequence (34-bp full-length tag: GTTTAATTGAGTTGTCATATGTTAATAACGGTAT, 29-bp tag: GTTTAATTGAGTTGTCATATGTTAATAAC, 24-bp tag: GTTTAATTGAGTTGTCATATGTTA, 19-bp tag GTTTAATTGAGTTGTCATA) was defined as tagmentation. PE-Analyzer (http://www.rgenome.net) was used to identify reads in which tagmentation occurred^40^. Tagmentation Case 1 and Case 2 were distinguished by sequence analysis. After TAPE-seq reads were analyzed by NGS, only the reads that contained full-length tag sequences were selected. The tag sequences were then removed from the sequences for analysis, and the remaining sequences were compared with the NGS reads from targeted deep sequencing of the cells that had undergone prime editing with pegRNAs without the tag sequence. Case 1 means that the editing pattern after the tag sequence is removed is the same as the editing pattern generated with the pegRNA lacking the tag sequence. If that pattern could not be found, the sequence was classified as Case 2.

### PiggyBac copy number analysis

To quantify the average copy number of integrated piggyBac transposons, we used a set of primers directed at the 5′ inverted repeat (IR) of the piggyBac vector. The sequences of the forward and reverse primers used to amplify the 5′ IR are 5′-CTAAATAGCGCGAATCCGTC-3′ and 5′-TCATTTTGACTCACGCGG-3′, respectively. Copy numbers were calculated using standard curves generated using a mixture of untransfected HEK293T genomic DNA and the serially diluted piggyBac plasmid with a known copy number. Real-time PCR was performed using a QuantStudio 3 Real-Time PCR System (Applied Biosystems) with PowerUp SYBR Green Master Mix (Applied Biosystems).

### FACS of GFP-expressing cells

Two weeks after puromycin selection, cells were washed with phosphate-buffered saline and detached from the plate with trypsin-EDTA. Cells were centrifuged at 500 × *g* for 5 min at room temperature and resuspended in phosphate-buffered saline with 2% FBS. GFP-positive cells were isolated using an Attune NxT Acoustic Focusing Cytometer (Thermo Scientific). Attune NxT software v4.2.0 was used to analyze the raw data.

### Statistics and reproducibility

10 sample sites, which were studied in the previous nDigenome-seq paper^31^, were analyzed. No data were excluded from the analyses. Results from the two-sided unpaired student t-test calculated by Prism (version 9.4.1) are shown.

### Reporting summary

Further information on research design is available in the Nature Portfolio Reporting Summary linked to this article.

## Supplementary information

Supplementary Information

Description of Additional Supplementary Files

Supplementary Data 1. pegRNA,epegRNA sequence

Supplementary Data 2. PE2 TAPE-seq on-target Tagmentation sequence analysis

Supplementary Data 3. On- and off-target loci identified by TAPE-seq and comparison with off-target loci identified by nDigenome-seq and GUIDE-seq

Supplementary Data 4. Sequencing platform and the number of reads generated for each TAPE-seq analysis

Supplementary Data 5. Validation by targeted deep sequencing of candidate off-target loci identified by TAPE-seq

Supplementary Data 6. Calculation of the validation rate for predictions made by TAPE-seq

Supplementary Data 7. Miss rate of the validated off-targets

Supplementary Data 8. Mismatch analysis by region

Supplementary Data 9. Off-target Validation

Supplementary Data 10. Vector construct sequence information

Reporting Summary

## Data Availability

Deep sequencing data that support the findings of this study have been deposited in NCBI Bioproject with the accession code PRJNA802977. Source Data is available as a Source Data file. Source data are provided with this paper.

## Source data

Source Data

## Code availability

Codes supporting the findings of this study have been archived online (https://github.com/PhyzenInc/TAPE-seq_flanking_depth).
