# Supplementary material for: TAPE-seq is a cell-based method for predicting genome-wide off-target effects of prime editor
Source: Nat Commun. 2022 Dec 29;13:7975. doi: 10.1038/s41467-022-35743-y (PMC9800413; doi:10.1038/s41467-022-35743-y)
Supplement: Supplementary file 1 — Supplementary Information [file 41467_2022_35743_MOESM1_ESM.pdf]

Supplementary Information

TAPE-seq is a cell-based method for predicting genome-wide off-target effects of prime editor

Contents:

Supplementary Figures 1 - 7

Supplementary Note 1

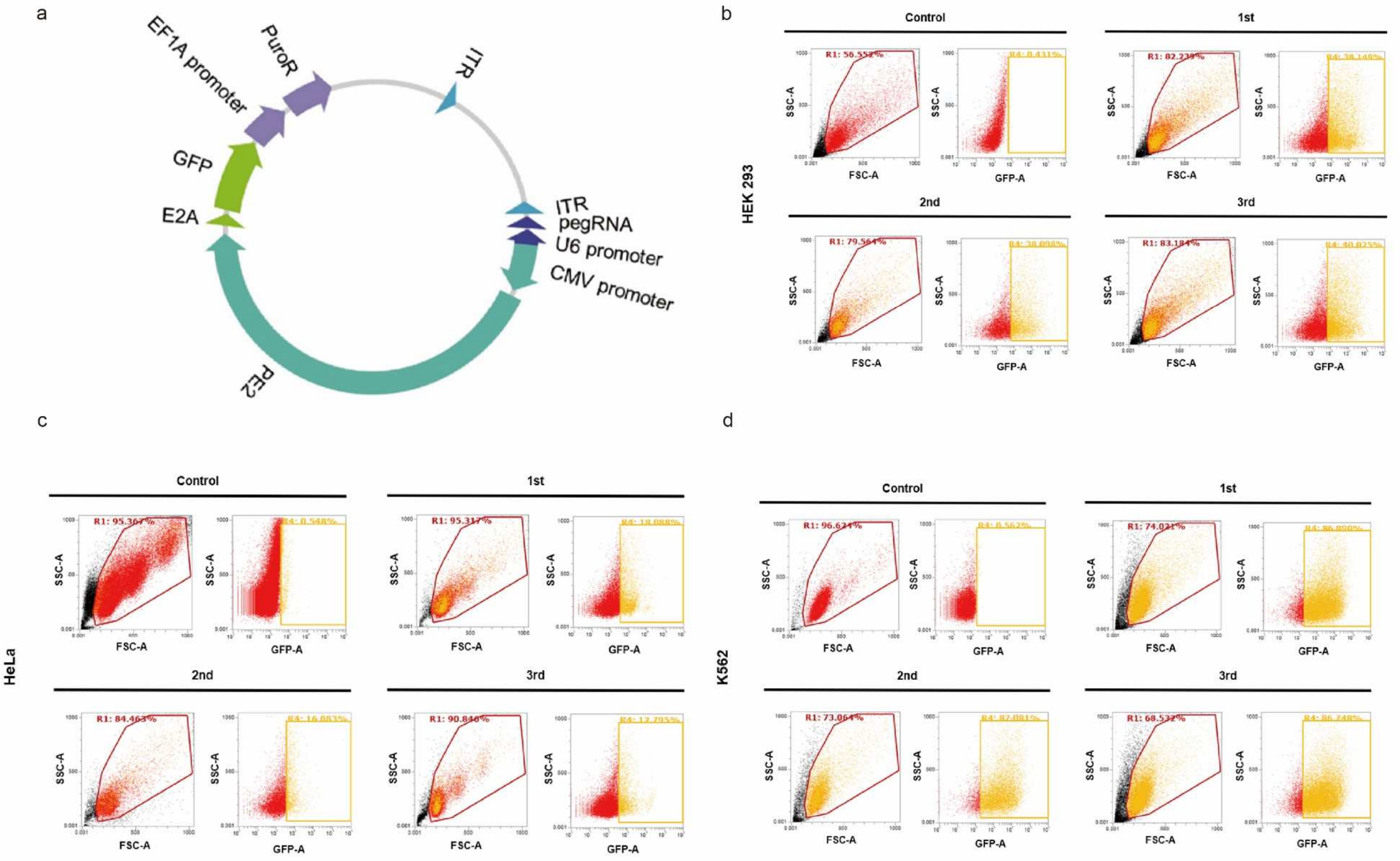
a
EF1A promoter
PuroR
ITR
GFP
E2A
ITR
pegRNA
U6 promoter
CMV promoter
PE2
b
HEK 293
Control
1st
2nd
3rd
SSC-A
FSC-A
GFP-A
c
HeLa
Control
1st
2nd
3rd
d
K562
Control
1st
2nd
3rd

Supplementary Figure 1. Puromycin selection for 14 days enriches cells transfected with the GFP-piggyback construct. (a) Map of the GFP-piggyback vector; GFP is linked to PE2 via an E2A sequence. (b-d) FACS-based analysis to detect GFP expression in three different transfected cell populations (HEK293T, HeLa, K562). Cell populations were gated by SSC-A vs. FSC-A and then the GFP+ population was plotted vs. SSC-A. Experiments were performed in triplicate (1st, 2nd and 3rd). (b) HEK293T, (c) HeLa, and (d) K562 cells.

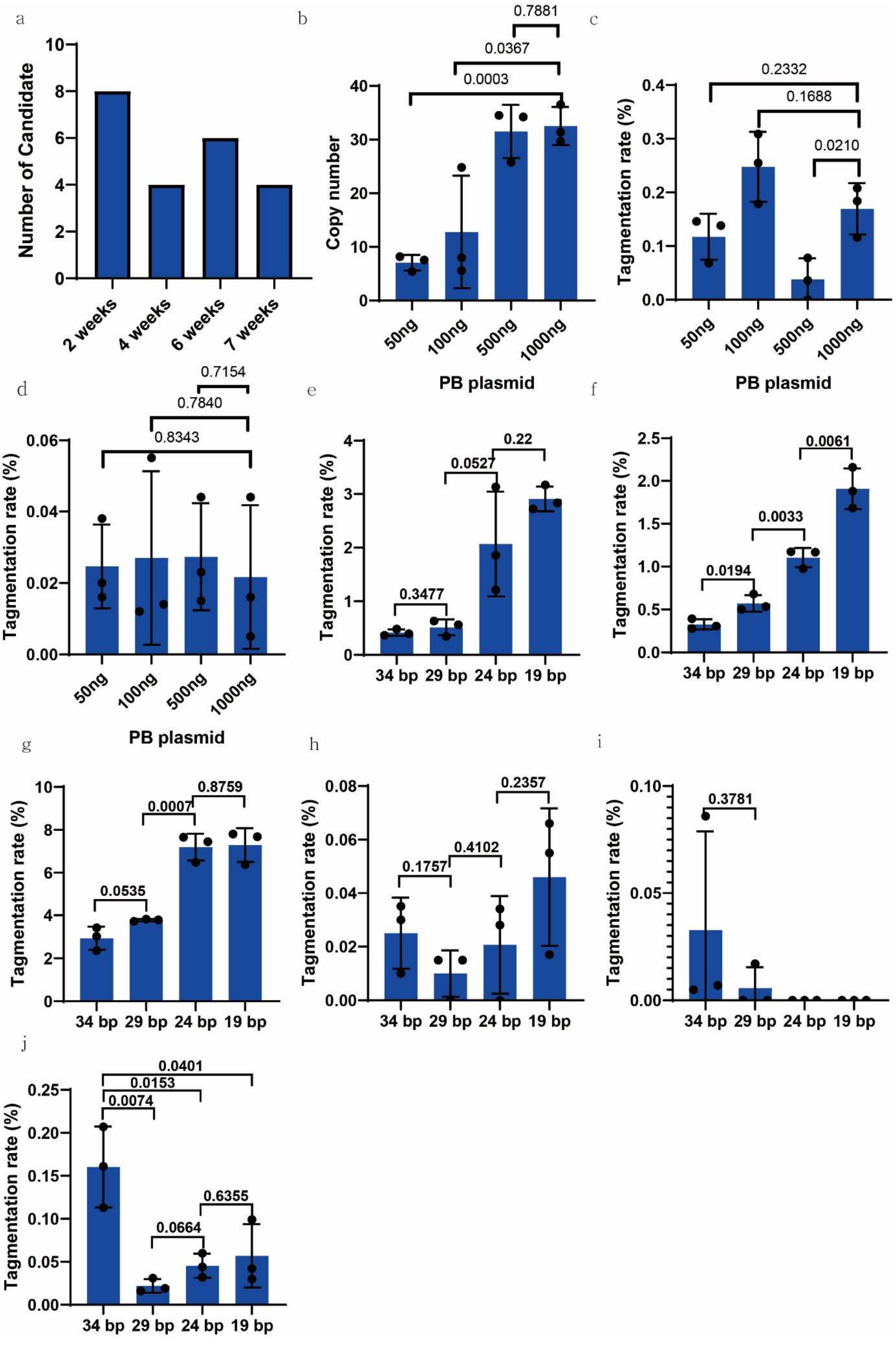
a
Number of Candidate
10
8
6
4
2
0
2 weeks
4 weeks
6 weeks
7 weeks
b
0.7881
0.0367
0.0003
Copy number
40
30
20
10
0
50ng
100ng
500ng
1000ng
PB plasmid
c
0.2332
0.1688
0.0210
Tagmentation rate (%)
0.4
0.3
0.2
0.1
0.0
50ng
100ng
500ng
1000ng
PB plasmid
d
0.7154
0.7840
0.8343
Tagmentation rate (%)
0.06
0.04
0.02
0.00
50ng
100ng
500ng
1000ng
PB plasmid
e
0.22
0.0527
0.3477
Tagmentation rate (%)
4
3
2
1
0
34 bp
29 bp
24 bp
19 bp
f
0.0061
0.0033
0.0194
Tagmentation rate (%)
2.5
2.0
1.5
1.0
0.5
0.0
34 bp
29 bp
24 bp
19 bp
g
0.0007
0.8759
0.0535
Tagmentation rate (%)
10
8
6
4
2
0
34 bp
29 bp
24 bp
19 bp
h
0.2357
0.4102
0.1757
Tagmentation rate (%)
0.08
0.06
0.04
0.02
0.00
34 bp
29 bp
24 bp
19 bp
i
0.3781
Tagmentation rate (%)
0.10
0.05
0.00
34 bp
29 bp
24 bp
19 bp
j
0.0401
0.0153
0.0074
0.6355
0.0664
Tagmentation rate (%)
0.25
0.20
0.15
0.10
0.05
0.00
34 bp
29 bp
24 bp
19 bp

Supplementary Figure 2. Optimization of tagmentation rates. (a) Number of candidate off-target sites found by TAPE-seq as a function of the incubation time following transfection of the *HEK4* (+2 G to T) pegRNA into HEK293T cells. (b) Copy number of the piggyBac construct found in cells via quantitative PCR as a function of the amount of piggyBac plasmid (ng) used to transfect HEK293T cells. (c) Tagmentation rate at the on-target site for the *HEK4* (+2 G to T) pegRNA as a function of the amount of piggyBac plasmid (ng) used to transfect HEK293T cells. (d) Tagmentation rate at off-target site 1 for the *HEK4* (+2 G to T) pegRNA as a function of the amount of piggyBac plasmid (ng) used to transfect HEK293T cells. (e, f, g) Tagmentation rate at the on-target site of the *HEK4* (+2 G to T) pegRNA as a function of the length of the probe sequence in (e) HEK293T, (f) HeLa, and (g) K562 cells. (h, i, j) Tagmentation rate at off-target site 1 of the *HEK4* (+2 G to T) pegRNA as a function of the length of the probe sequence in (h) HEK293T, (i) HeLa, and (j) K562 cells. The bars represent the mean. Error bars indicate standard deviation (n=3 independent transfection). Two-sided unpaired student-t test. PB, piggyBac.

a

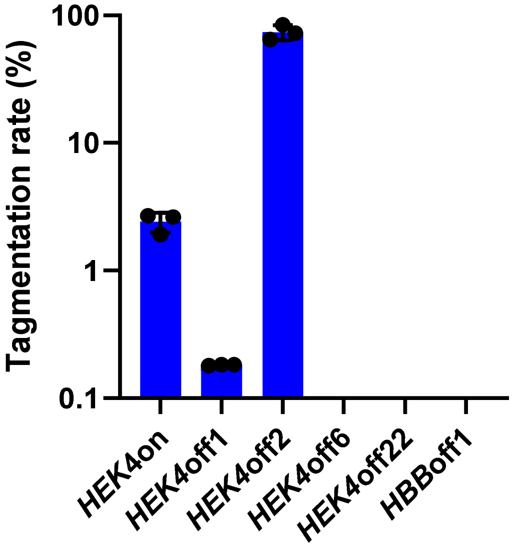

c

b

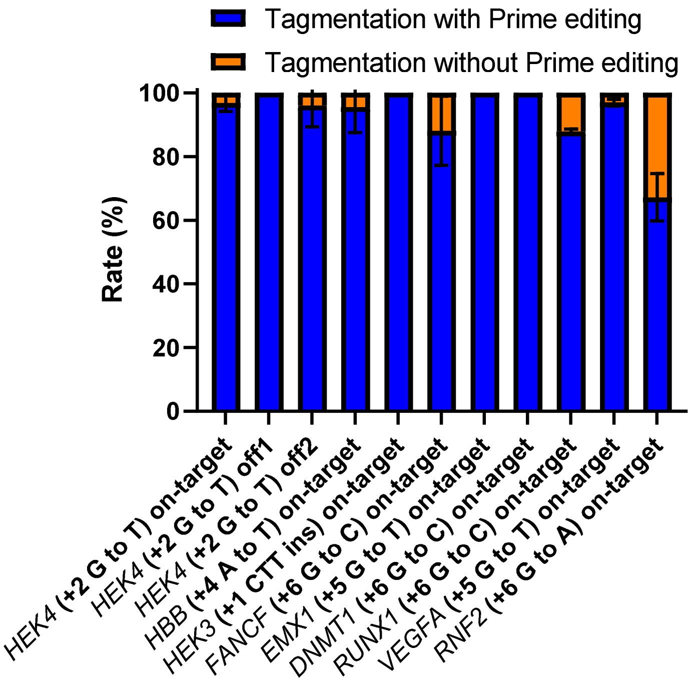

Supplementary Figure 3. (a) Tagmentation rates at six different target sites of the *HEK4* (+2 G to T) and *HBB* (+4 A to T) pegRNAs measured by targeted deep sequencing. (b) Ratio of tagmentation rates with or without prime editing when ten different on-target and off-target loci were analyzed. The bars represent the mean. Error bars indicate standard deviation (n=3 independent transfections).

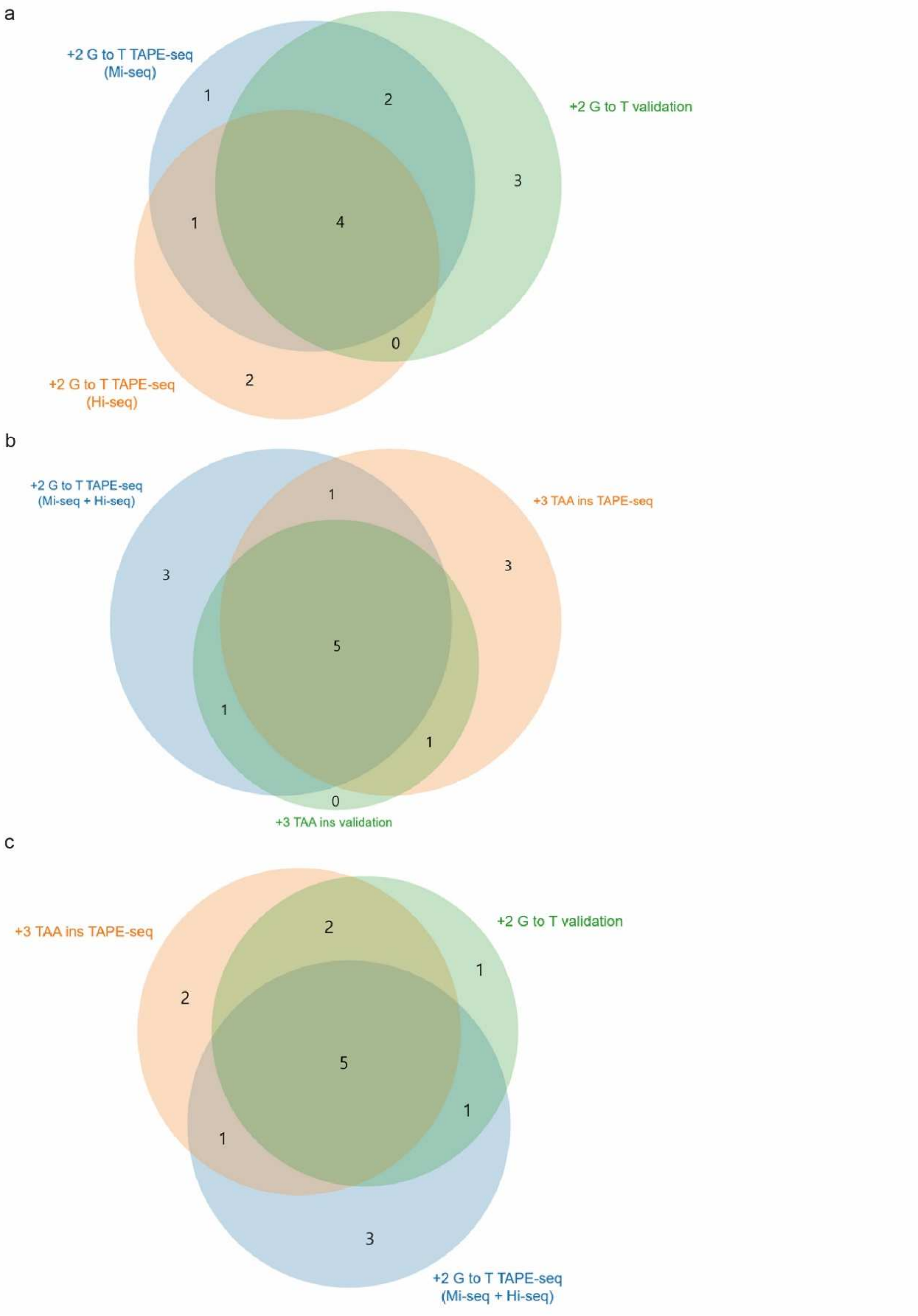
a
+2 G to T TAPE-seq (Mi-seq)
+2 G to T validation
+2 G to T TAPE-seq (Hi-seq)
1
2
3
1
4
0
2
b
+2 G to T TAPE-seq (Mi-seq + Hi-seq)
+3 TAA ins TAPE-seq
+3 TAA ins validation
1
3
3
5
1
1
0
c
+3 TAA ins TAPE-seq
+2 G to T validation
+2 G to T TAPE-seq (Mi-seq + Hi-seq)
2
1
2
5
1
1
3

Supplementary Figure 4. Venn diagrams showing the number of and overlap between off-target sites of *HEK4*-targeted pegRNAs predicted by TAPE-seq compared to the number of validated sites. (a) *HEK4* (+2 G to T) validation, *HEK4* (+2 G to T) predicted by TAPE-seq (Mi-seq), and *HEK4* (+2 G to T) predicted by TAPE-seq (Hi-seq); (b) *HEK4* (+3 TAA ins) validation, *HEK4* (+2 G to T) predicted by TAPE-seq (Mi-seq + Hi-seq), and *HEK4* (+3 TAA ins) predicted by TAPE-seq; (c) *HEK4* (+2 G to T) validation, *HEK4* (+2 G to T) predicted by TAPE-seq (Mi-seq + Hi-seq), and *HEK4* (+3 TAA ins) predicted by TAPE-seq.

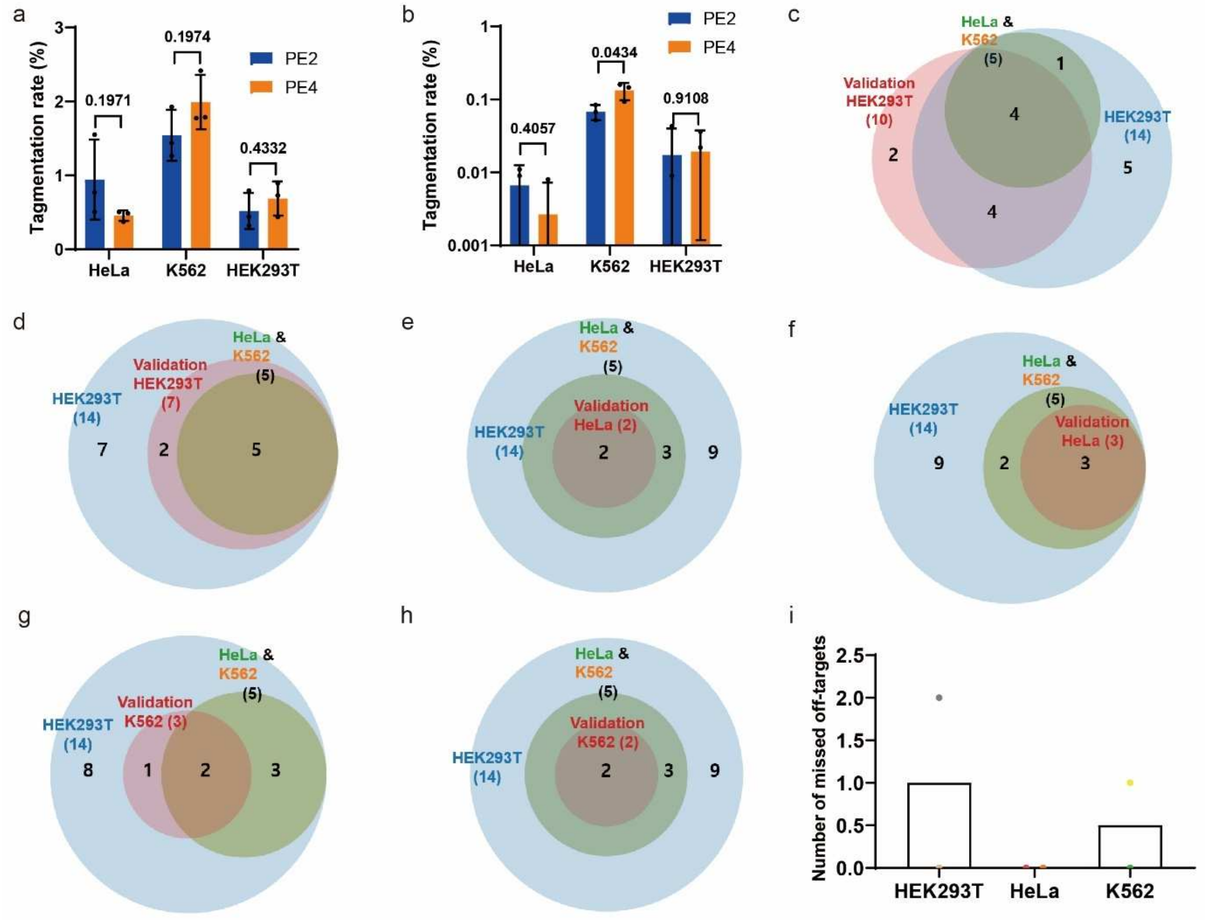

Supplementary Figure 5. (a, b) Tagmentation rates for the *HEK4* (+2 G to T) pegRNA at (a) the on-target site and (b) off-target site 1 in HEK293T, HeLa, and K562 cells. (c-h) Venn diagrams showing the number of and overlap between off-target sites predicted by TAPE-seq and validated sites for the (c) *HEK4* (+2 G to T) pegRNA in HEK293T cells, (d) the *HEK4* (+3 TAA ins) pegRNA in HEK293T cells, (e) the *HEK4* (+2 G to T) pegRNA in HeLa cells, (f) the *HEK4* (+3 TAA ins) pegRNA in HeLa cells, (g) the *HEK4* (+2 G to T) pegRNA in K562 cells, and (h) the *HEK4* (+3 TAA ins) pegRNA in K562 cells. (i) Number of missed off-target sites within each cell type. The bars represent the mean. Error bars indicate standard deviation (n=3 independent transfections for (a) and (b), n=2 independent experiments for (i) each represented by different colour). Two-sided unpaired student t-test.

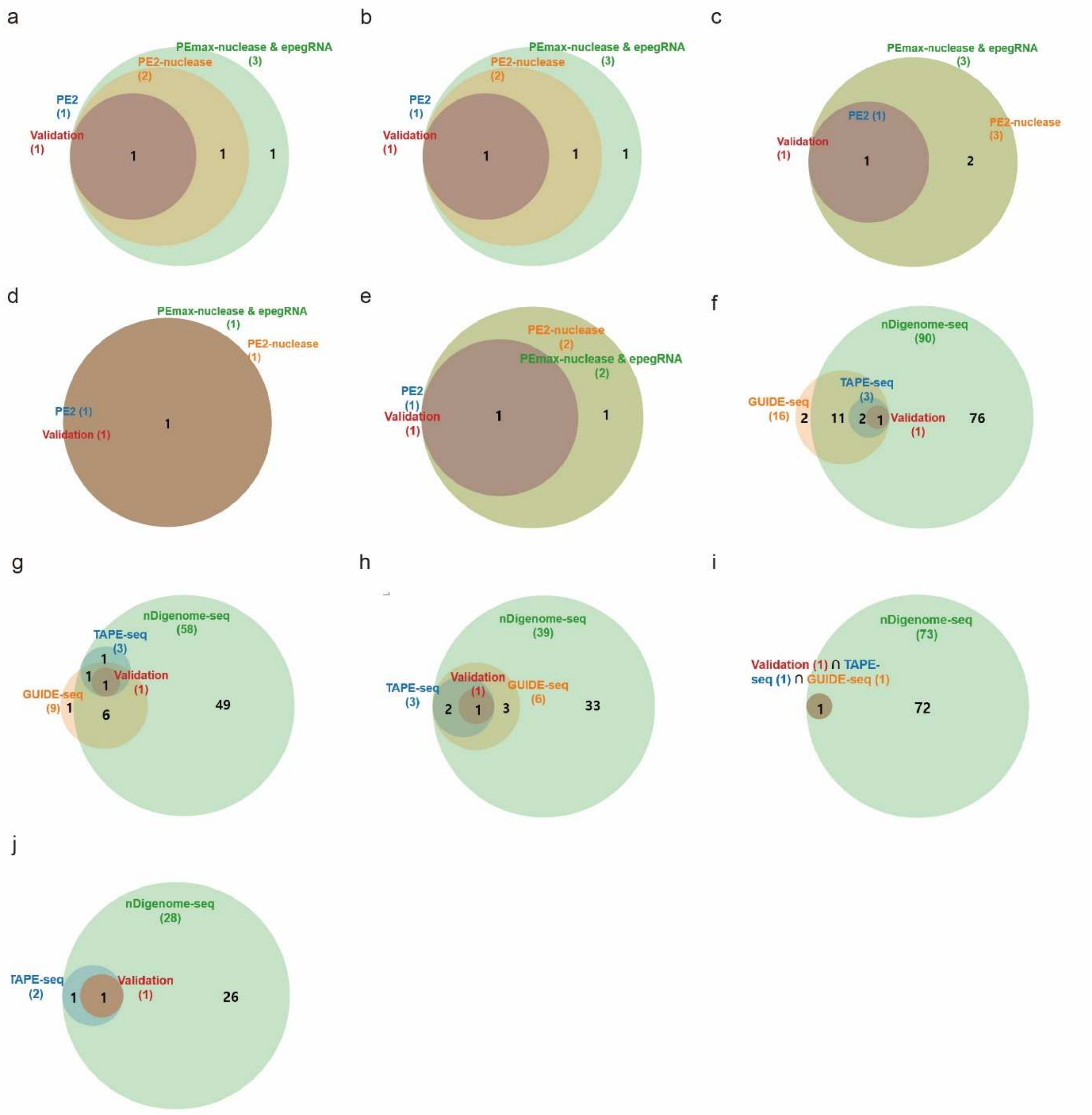

Supplementary Figure 6. (a-e) Venn diagrams showing the number of and overlap between off-target loci predicted by TAPE-seq using PE2, PE2-nuclease, and PEmax-nuclease with epegRNAs and validated off-target sites for the (a) *EMX1* (+5 G to T), (b) *FANCF* (+6 G to C), (c) *HEK3* (+1 CTT ins), (d) *RNF2* (+6 G to A), and (e) *RUNX1* (+6 G to C) pegRNAs. (f-j) Venn diagrams showing the number of and overlap between off-target loci predicted by TAPE-seq using PE2, GUIDE-seq, and nDigenome-seq and validated off-target sites for the (f) *EMX1* (+5 G to T), (g) *FANCF* (+6 G to C), (h) *HEK3* (+1 CTT ins), (i) *RNF2* (+6 G to A), and (j) *RUNX1* (+6 G to C) pegRNAs.

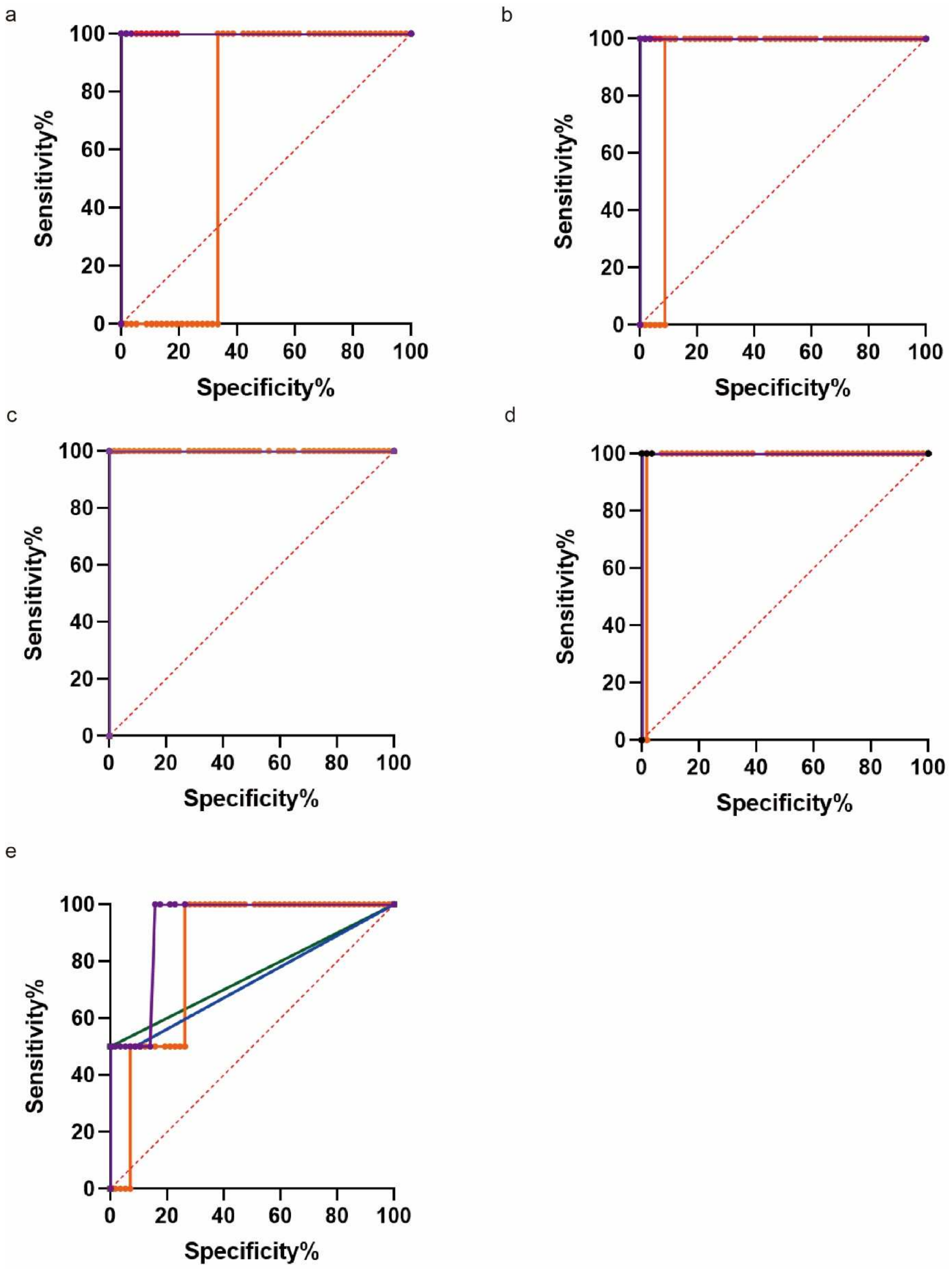
a
b
c
d
e
Sensitivity%
Specificity%
0
20
40
60
80
100

Supplementary Figure 7. ROC curves for GUIDE-seq (red), nDigenome-seq (orange), and TAPE-seq using PE2 (green), PE2-nuclease (blue), and PEmax-nuclease with epegRNAs (purple) for the (a) *EMX1* (+5 G to T), (b) *FANCF* (+6 G to C), (c) *RNF2* (+6 G to A), (d) *RUNX1* (+6 G to C), and (e) *VEGFA* (+5 G to T) pegRNAs.

Supplementary Note 1. TAPE-seq library preparation protocol

Reagents		
Item	Catalog	Vendor
Blood Genomic DNA Extraction Mini Kit	FABGK 001	Favorgen
AMPure XP beads	A63881	Beckman coulter
NEBNext® Ultra™ II DNA Library Prep Kit for Illumina	E7645L	NEB
NEBNext adaptor (from NEBNext Singleplex kit)	E7350	NEB
Ethyl Alcohol 99.9% GR grade	UN1170	DUKSAN
TMAC Buffer, 5M	T3411	Sigma Aldrich
1X TE Buffer	12090015	Invitrogen
Platinum® Taq DNA Polymerase	10966026	Invitrogen
dNTP Mix, 2.5 mM each	EBN-1006	Elpis biotech
Nuclease-Free Water	AM9932	Ambion
Q5 High-Fidelity DNA Polymerase	M0491L	NEB
microTUBE-50 AFA Fiber Screw-Cap	520166	Covaris

Equipment	
Item	Vendor
M220 Focused-ultrasonicator	Covaris
T100 Thermocycler	Bio rad
NEBNext® Magnetic Separation Rack	NEB
Nanodrop One C	Thermo
MiSeq	Illumina
HiSeq	Illumina

Oligonucleotide	Sequence
GSP1+	ATACCGTTATTAACATATGACA
GSP1-	GTTTAATTGAGTTGTCATATGTTAATAAC
GSP2+	GTGACTGGAGTTCAGACGTGTGCTCTTCCGATCTACATATGACAACTCAATTAAAC
GSP2-	GTGACTGGAGTTCAGACGTGTGCTCTTCCGATCTTTGAGTTGTCATATGTTAATAACGGTA
Index Forward (illumina D501-508 index, N's denote Index sequences)	AATGATACGGCGACCACCGAGATCTACACNNNNNNNNACACTCTTTCCCTACACGACG
Index Reverse (illumina D701-712 index)	CAAGCAGAAGACGGCATACGAGATTANNNNNNNNGACTGGAGTTCAGACGTGTGCTC

Genomic DNA shearing

1. Isolate genomic DNA (gDNA) using a Blood Genomic DNA Extraction Mini Kit, then elute the purified gDNA with 1X TE buffer and determine its concentration by nanodrop.
2. Shear 5 μg of gDNA to an average length of 325 bp with an M220 Focused-ultrasonicator. Detailed sonication conditions are indicated below.

Shearing conditions	
Tube	microTUBE-50 AFA Fiber Screw-Cap
Sample Volume	55 μl
Temperature (°C)	20
Peak Incident Power (W)	75
Duty Factor (%)	10
Cycles per Burst (cpb)	200
Treatment Time (sec)	90

3. Clean the sheared gDNA with 55 μl of AMPure XP beads (1X ratio) according to the manufacturer's protocol, and elute it in 50 μl 1X TE buffer.

End repair, A-tailing, and NEBNext adaptor ligation

4. Using an NEBNext® Ultra™ II DNA Library Prep Kit for Illumina, ligate 1 μg of sheared gDNA with NEBNext adaptors. (One TAPE-seq reaction needs two vials of adaptor-ligated gDNA for +(sense) and -(antisense) library construction.)
5. All steps should be conducted using the manufacturer's protocol, except that adaptor ligation and USER enzyme treatment reaction times have been modified (adaptor ligation, 1 hr / USER enzyme treatment, 30 min).
6. Purify the reaction product with 0.9X AMPure XP beads, and elute it in 12 μl nuclease-free water.

PCRs for Tag-specific library amplification

Modified GUIDE-seq Discovery PCR is used for Tag-specific library amplification. For +(sense) and -(antisense) library construction, GSP+ and GSP- primers should be separately used for each + and - PCR reaction.

7. Prepare two vials of the 1st PCR reaction for the + and - libraries.

1st PCR mixture

Nuclease-free water	10.1 µl
Buffer for Taq Polymerase, 10X (MgCl2 free)	3.0 µl
dNTP Mix, 2.5 mM each	2.4 µl
MgCl2, 50 mM	1.2 µl
Platinum Taq polymerase, 5 U/µl	0.3 µl
GSP1+ or GSP1- primer (10uM)*	0.75 µl
D5_#(Index forward D501-D508)	0.75 µl
TMAC (0.5M)	1.5 µl
DNA sample(from step 6)	10.0 µl
Total	30.0 µl
1st PCR Thermal cycler condition	
95°C for 5 min,<br>15 cycles of [95°C for 30 s, 70°C (-1°C/cycle) for 2 min, 72°C for 30 s],<br>10 cycles of [95°C for 30 s, 55°C for 1 min, 72°C for 30 s],<br>72°C for 5 min,<br>4°C hold	

*proceed with separate PCR reactions

8. Clean the PCR product with 0.9X AMPure XP beads and elute it in 20 µl nuclease-free water.

9. Prepare the 2nd PCR.

2nd PCR mixture	
Nuclease-free water	3.6 µl
Buffer for Taq Polymerase, 10X (MgCl2 free)	3.0 µl
dNTP Mix, 2.5 mM each	2.4 µl
MgCl2, 50 mM	1.2 µl
Platinum Taq polymerase, 5 U/µl	0.3 µl
GSP2+ or GSP2- Primer (10uM)*	1.5 µl
D5_#(Index forward D501-D508)	1.5 µl
TMAC (0.5M)	1.5 µl
DNA sample(from step 8)	15.0 µl
Total	30.0 µl
2nd PCR Thermal cycler condition	
95°C for 5 min,<br>15 cycles of [95°C for 30 s, 70°C (-1°C/cycle) for 2 min, 72°C for 30 s],<br>10 cycles of [95°C for 30 s, 55°C for 1 min, 72°C for 30 s],<br>72°C for 5 min,<br>4°C hold	

*proceed with separate PCR reactions

10. Clean the PCR product with 0.7X AMPure XP beads and elute it in 15 µl nuclease-free water.

11. Prepare the 3rd PCR.

3rd PCR mixture	
Nuclease-free water	20.5 µl
5X Q5 Reaction Buffer	10.0 µl
dNTP Mix, 2.5 mM each	4.0 µl
Q5 High-Fidelity DNA Polymerase	0.5 µl
D7_#(Index reverse D701-712)*	2.5 µl
D5_#(Index forward D501-D508)	2.5 µl
DNA sample(from step 10)	10.0 µl
Total	50.0 µl
3rd PCR Thermal cycler condition	
98°C for 30 s<br>30 cycles of [98°C for 10 s, 58°C for 20 s, 72°C for 30 s],<br>72°C for 5 min,<br>4°C hold	

12. Clean the PCR product with 0.7X AMPure XP beads and elute it in 60 µl of nuclease-free water.

13. Analyze the purified PCR products with 2x150-bp paired end Mi-seq or Hi-seq.
