# Supplementary material for: TAPE-seq is a cell-based method for predicting genome-wide off-target effects of prime editor
Source: Nat Commun. 2022 Dec 29;13:7975. doi: 10.1038/s41467-022-35743-y (PMC9800413; doi:10.1038/s41467-022-35743-y)
Supplement: Supplementary file 2 — Description of Additional Supplementary Files [file 41467_2022_35743_MOESM2_ESM.pdf]

Title: Supplementary Data 1.

Description: pegRNA,epegRNA sequence (Provided as a separate Excel file).

Title: Supplementary Data 2.

Description: PE2 TAPE-seq on-target Tagmentation sequence analysis (Provided as a separate Excel file).

Title: Supplementary Data 3.

Description: On- and off-target loci identified by TAPE-seq and comparison with off-target loci identified by nDigenome-seq and GUIDE-seq for sgRNAs targeting the same site, if available (Provided as a separate Excel file).

Title: Supplementary Data 4.

Description: Sequencing platform and the number of reads generated for each TAPE-seq analysis (Provided as a separate Excel file).

Title: Supplementary Data 5.

Description: Validation by targeted deep sequencing of candidate off-target loci identified by TAPE-seq (Provided as a separate Excel file).

Title: Supplementary Data 6.

Description: Calculation of the validation rate for predictions made by TAPE-seq (Provided as a separate Excel file).

Title: Supplementary Data 7.

Description: Miss rate of the validated off-targets (Provided as a separate Excel file).

Title: Supplementary Data 8.

Description: Mismatch analysis by region (Provided as a separate Excel file).

Title: Supplementary Data 9.

Description: Off-target Validation (Provided as a separate Power-point file).

Title: Supplementary Data 10.

Description: Vector construct sequence information (Provided as a separate Word file).
