# Supplementary material for: TAPE-seq is a cell-based method for predicting genome-wide off-target effects of prime editor
Source: Nat Commun. 2022 Dec 29;13:7975. doi: 10.1038/s41467-022-35743-y (PMC9800413; doi:10.1038/s41467-022-35743-y)
Supplement: Supplementary file 11 — Supplementary Data 9. Off-target Validation [file 41467_2022_35743_MOESM11_ESM.pptx]

## Slide 1
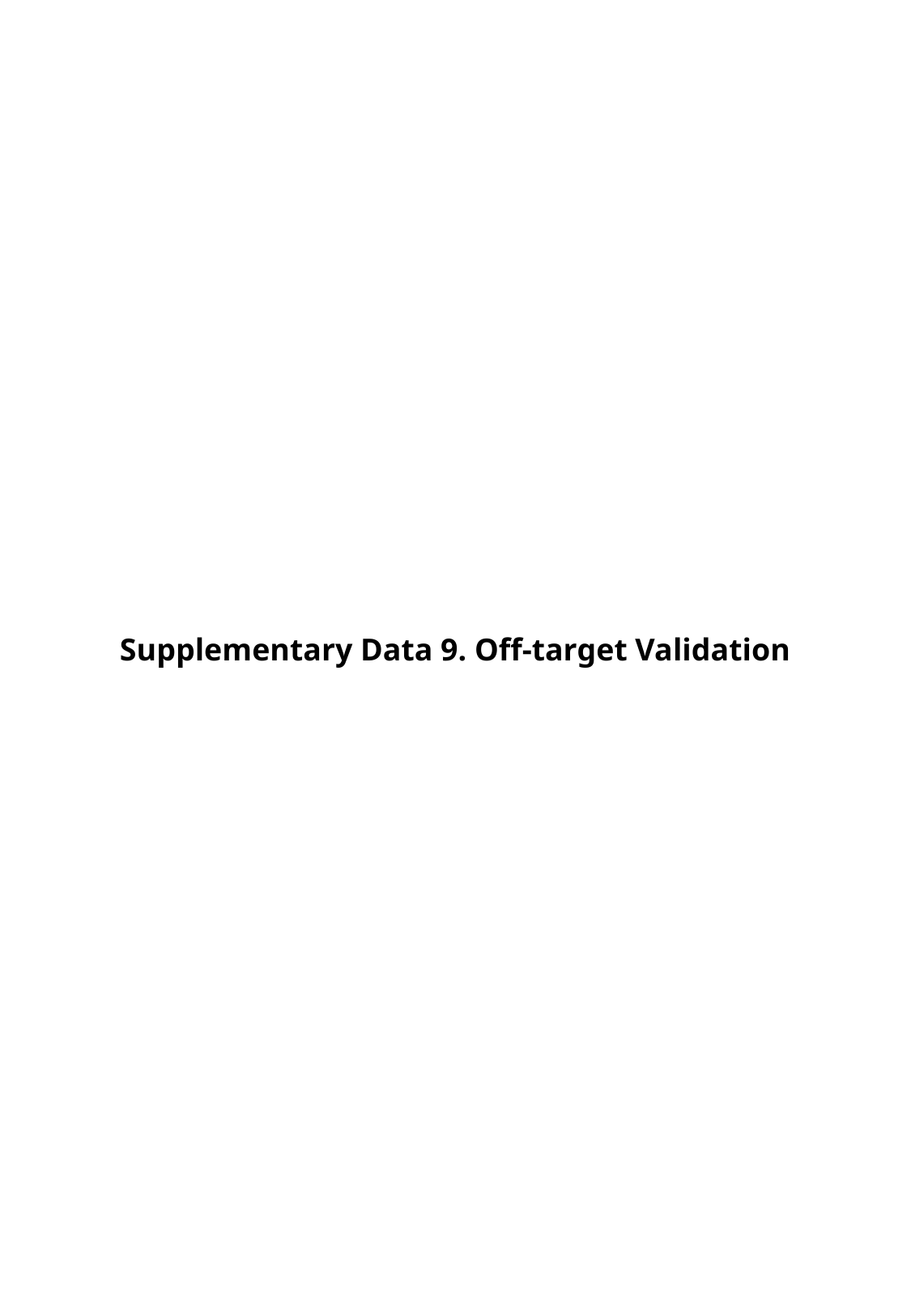

# Supplementary Data 9. Off-target Validation

## Slide 2
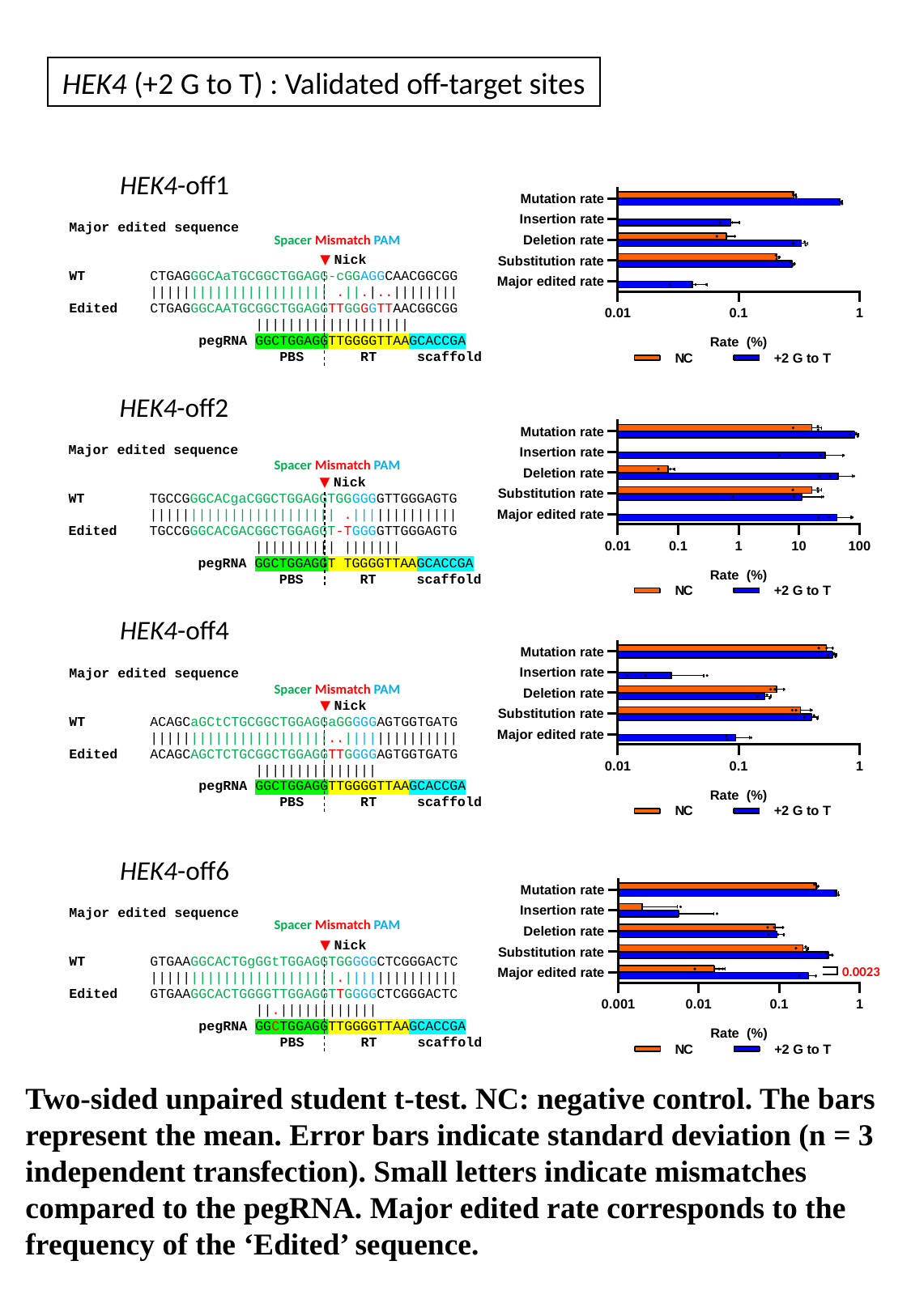

HEK4 (+2 G to T) : Validated off-target sites
HEK4-off1
Major edited sequence
 ▼ Nick
WT CTGAGGGCAaTGCGGCTGGAGG-cGGAGGCAACGGCGG
 |||||||||||||||||||||| .||.|..||||||||
Edited CTGAGGGCAATGCGGCTGGAGGTTGGGGTTAACGGCGG
 |||||||||||||||||||
 pegRNA GGCTGGAGGTTGGGGTTAAGCACCGA
 PBS RT scaffold
Spacer Mismatch PAM
HEK4-off2
Major edited sequence
 ▼ Nick
WT TGCCGGGCACgaCGGCTGGAGGTGGGGGGTTGGGAGTG
 ||||||||||||||||||||||| .|||||||||||||
Edited TGCCGGGCACGACGGCTGGAGGT-TGGGGTTGGGAGTG
 |||||||||| |||||||
 pegRNA GGCTGGAGGT TGGGGTTAAGCACCGA
 PBS RT scaffold
Spacer Mismatch PAM
HEK4-off4
Major edited sequence
 ▼ Nick
WT ACAGCaGCtCTGCGGCTGGAGGaGGGGGAGTGGTGATG
 ||||||||||||||||||||||..||||||||||||||
Edited ACAGCAGCTCTGCGGCTGGAGGTTGGGGAGTGGTGATG
 |||||||||||||||
 pegRNA GGCTGGAGGTTGGGGTTAAGCACCGA
 PBS RT scaffold
Spacer Mismatch PAM
HEK4-off6
Major edited sequence
 ▼ Nick
WT GTGAAGGCACTGgGGtTGGAGGTGGGGGCTCGGGACTC
 |||||||||||||||||||||||.||||||||||||||
Edited GTGAAGGCACTGGGGTTGGAGGTTGGGGCTCGGGACTC
 ||.||||||||||||
 pegRNA GGCTGGAGGTTGGGGTTAAGCACCGA
 PBS RT scaffold
0.0023
Spacer Mismatch PAM
Two-sided unpaired student t-test. NC: negative control. The bars represent the mean. Error bars indicate standard deviation (n = 3 independent transfection). Small letters indicate mismatches compared to the pegRNA. Major edited rate corresponds to the frequency of the ‘Edited’ sequence.

## Slide 3
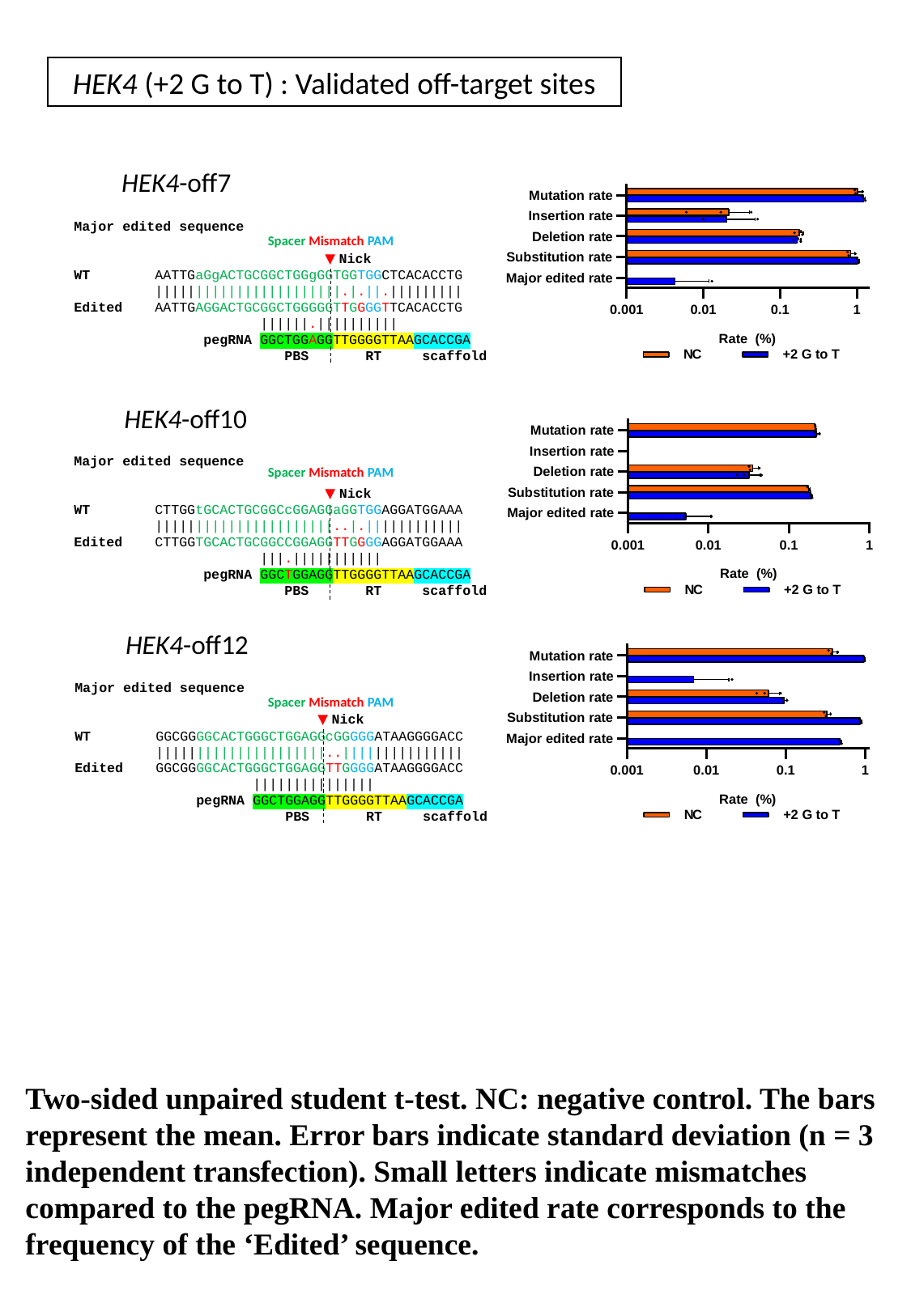

HEK4 (+2 G to T) : Validated off-target sites
HEK4-off7
Major edited sequence
 ▼ Nick
WT AATTGaGgACTGCGGCTGGgGGTGGTGGCTCACACCTG
 |||||||||||||||||||||||.|.||.|||||||||
Edited AATTGAGGACTGCGGCTGGGGGTTGGGGTTCACACCTG
 ||||||.||||||||||
 pegRNA GGCTGGAGGTTGGGGTTAAGCACCGA
 PBS RT scaffold
Spacer Mismatch PAM
HEK4-off10
Major edited sequence
 ▼ Nick
WT CTTGGtGCACTGCGGCcGGAGGaGGTGGAGGATGGAAA
 ||||||||||||||||||||||..|.||||||||||||
Edited CTTGGTGCACTGCGGCCGGAGGTTGGGGAGGATGGAAA
 |||.|||||||||||
 pegRNA GGCTGGAGGTTGGGGTTAAGCACCGA
 PBS RT scaffold
Spacer Mismatch PAM
HEK4-off12
Major edited sequence
 ▼ Nick
WT GGCGGGGCACTGGGCTGGAGGcGGGGGATAAGGGGACC
 |||||||||||||||||||||..|||||||||||||||
Edited GGCGGGGCACTGGGCTGGAGGTTGGGGATAAGGGGACC
 |||||||||||||||
 pegRNA GGCTGGAGGTTGGGGTTAAGCACCGA
 PBS RT scaffold
Spacer Mismatch PAM
Two-sided unpaired student t-test. NC: negative control. The bars represent the mean. Error bars indicate standard deviation (n = 3 independent transfection). Small letters indicate mismatches compared to the pegRNA. Major edited rate corresponds to the frequency of the ‘Edited’ sequence.

## Slide 4
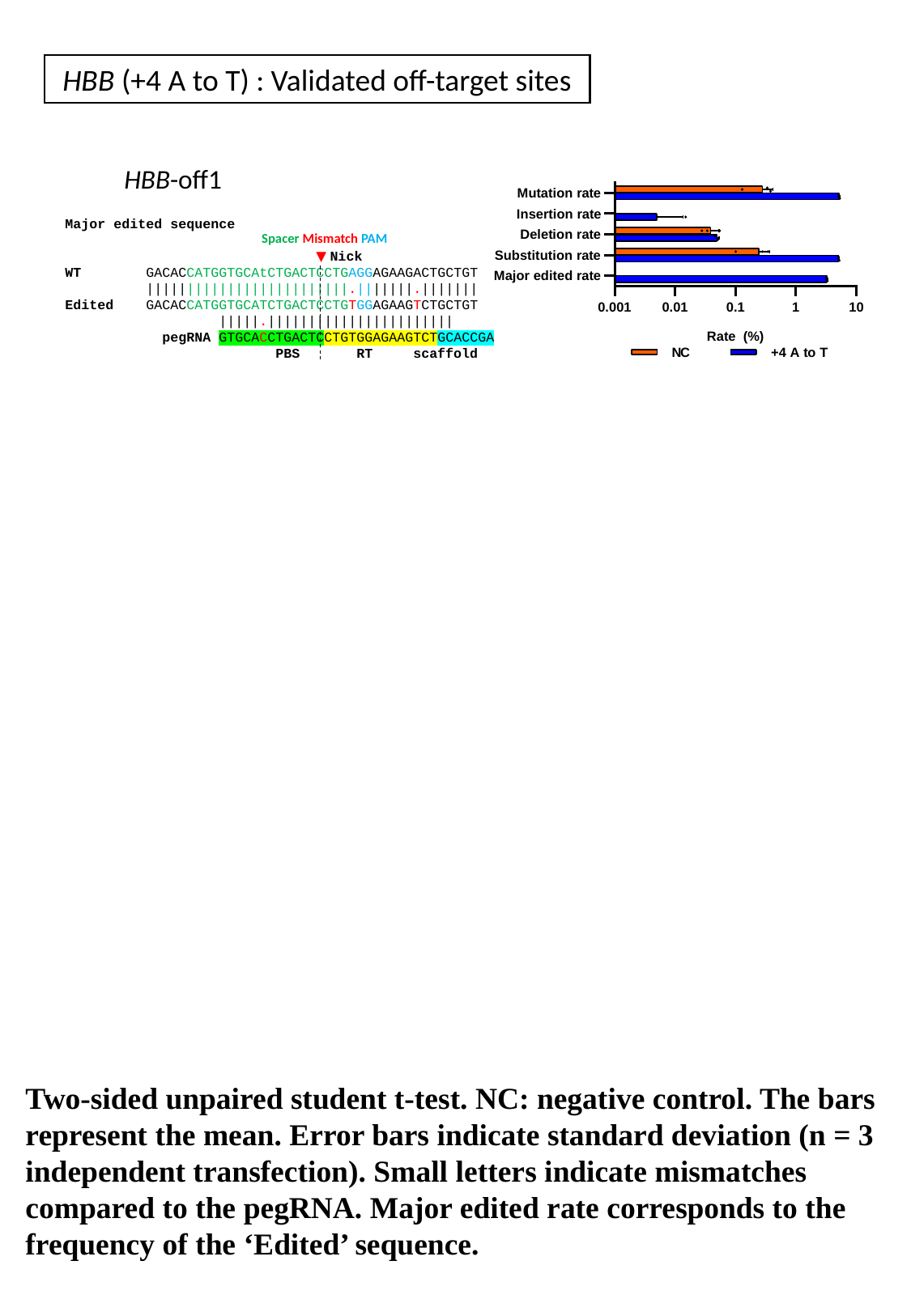

HBB (+4 A to T) : Validated off-target sites
HBB-off1
Major edited sequence
 ▼ Nick
WT GACACCATGGTGCAtCTGACTCCTGAGGAGAAGACTGCTGT
 |||||||||||||||||||||||||.|||||||.|||||||
Edited GACACCATGGTGCATCTGACTCCTGTGGAGAAGTCTGCTGT
 |||||.|||||||||||||||||||||||
 pegRNA GTGCACCTGACTCCTGTGGAGAAGTCTGCACCGA
 PBS RT scaffold
Spacer Mismatch PAM
Two-sided unpaired student t-test. NC: negative control. The bars represent the mean. Error bars indicate standard deviation (n = 3 independent transfection). Small letters indicate mismatches compared to the pegRNA. Major edited rate corresponds to the frequency of the ‘Edited’ sequence.

## Slide 5
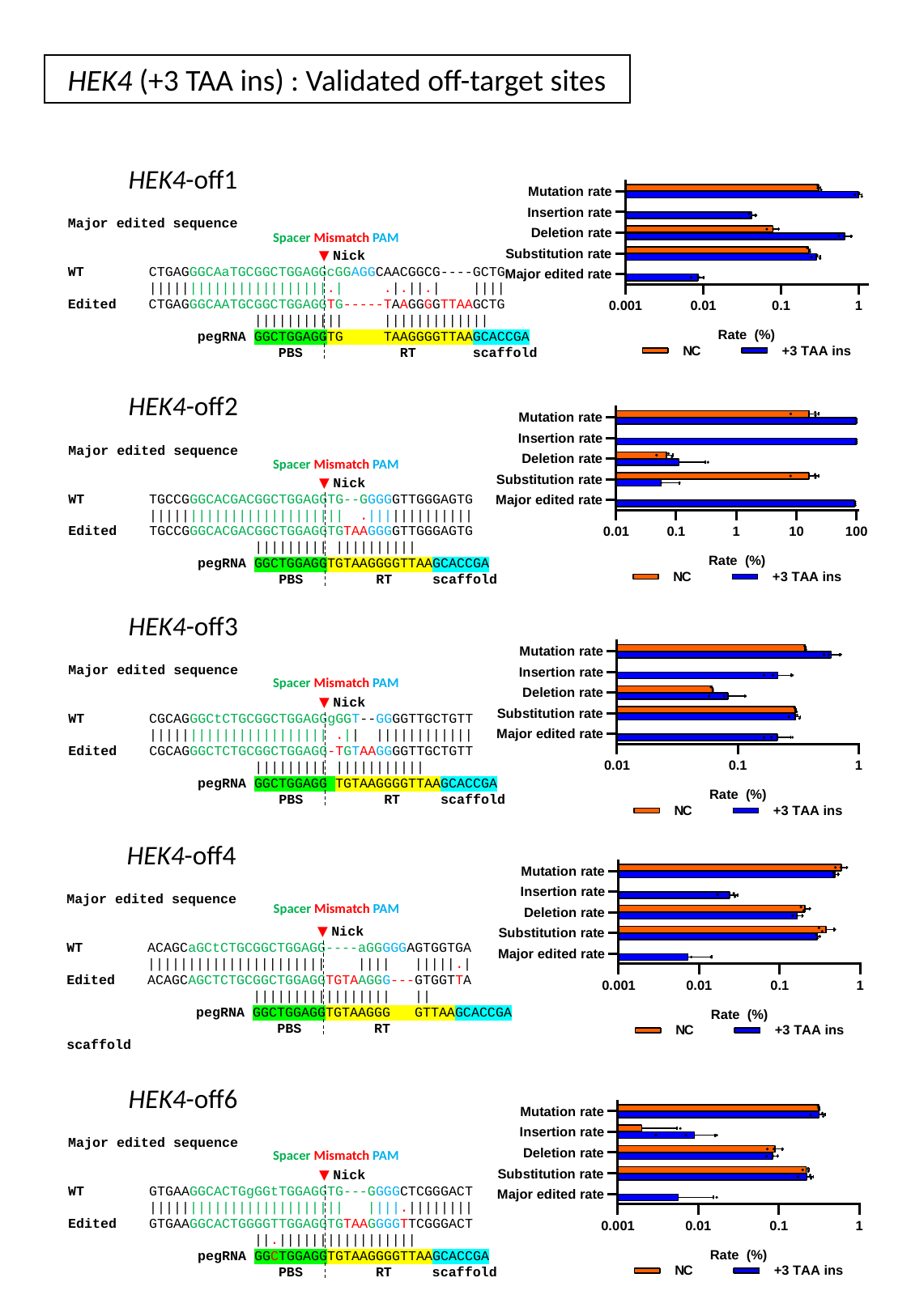

HEK4 (+3 TAA ins) : Validated off-target sites
HEK4-off1
Major edited sequence
 ▼ Nick
WT CTGAGGGCAaTGCGGCTGGAGGcGGAGGCAACGGCG----GCTG
 ||||||||||||||||||||||.|     .|.||.|    ||||
Edited CTGAGGGCAATGCGGCTGGAGGTG-----TAAGGGGTTAAGCTG
 ||||||||||| |||||||||||||
 pegRNA GGCTGGAGGTG TAAGGGGTTAAGCACCGA
 PBS RT scaffold
Spacer Mismatch PAM
HEK4-off2
Major edited sequence
 ▼ Nick
WT TGCCGGGCACGACGGCTGGAGGTG--GGGGGTTGGGAGTG
 ||||||||||||||||||||||||  .|||||||||||||
Edited TGCCGGGCACGACGGCTGGAGGTGTAAGGGGTTGGGAGTG
 ||||||||| ||||||||||
 pegRNA GGCTGGAGGTGTAAGGGGTTAAGCACCGA
 PBS RT scaffold
Spacer Mismatch PAM
HEK4-off3
Major edited sequence
 ▼ Nick
WT CGCAGGGCtCTGCGGCTGGAGGgGGT--GGGGTTGCTGTT
 |||||||||||||||||||||| .||  ||||||||||||
Edited CGCAGGGCTCTGCGGCTGGAGG-TGTAAGGGGTTGCTGTT
 ||||||||| |||||||||||
 pegRNA GGCTGGAGG TGTAAGGGGTTAAGCACCGA
 PBS RT scaffold
Spacer Mismatch PAM
HEK4-off4
Major edited sequence
 ▼ Nick
WT ACAGCaGCtCTGCGGCTGGAGG----aGGGGGAGTGGTGA
 ||||||||||||||||||||||    ||||   |||||.|
Edited ACAGCAGCTCTGCGGCTGGAGGTGTAAGGG---GTGGTTA
 ||||||||||||||||| ||
 pegRNA GGCTGGAGGTGTAAGGG GTTAAGCACCGA
 PBS RT scaffold
Spacer Mismatch PAM
HEK4-off6
Major edited sequence
 ▼ Nick
WT GTGAAGGCACTGgGGtTGGAGGTG---GGGGCTCGGGACT
 ||||||||||||||||||||||||   ||||.||||||||
Edited GTGAAGGCACTGGGGTTGGAGGTGTAAGGGGTTCGGGACT
 ||.|||||||||||||||||
 pegRNA GGCTGGAGGTGTAAGGGGTTAAGCACCGA
 PBS RT scaffold
Spacer Mismatch PAM

## Slide 6
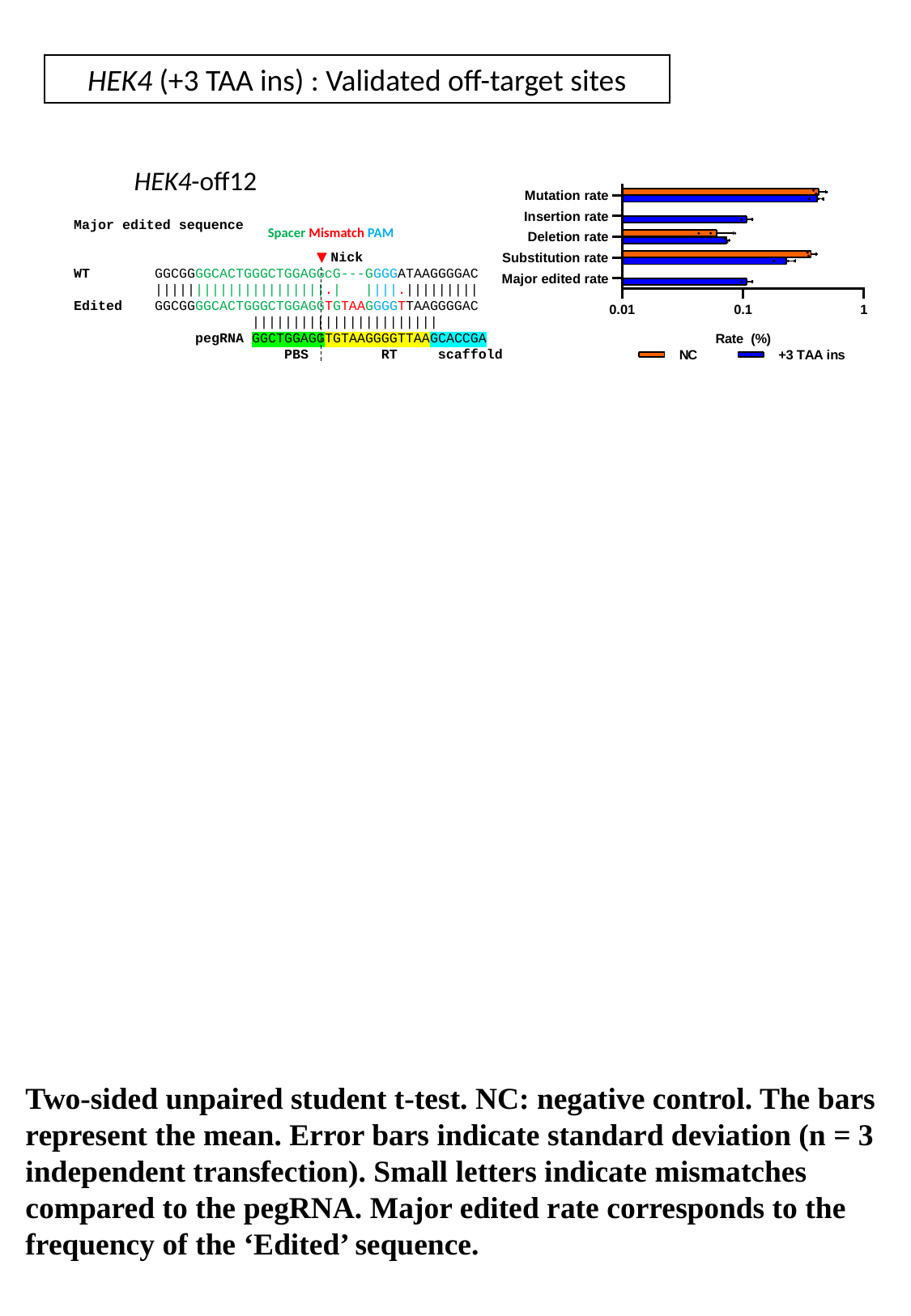

HEK4 (+3 TAA ins) : Validated off-target sites
HEK4-off12
Major edited sequence
 ▼ Nick
WT GGCGGGGCACTGGGCTGGAGGcG---GGGGATAAGGGGAC
 |||||||||||||||||||||.|   ||||.|||||||||
Edited GGCGGGGCACTGGGCTGGAGGTGTAAGGGGTTAAGGGGAC
 |||||||||||||||||||||||
 pegRNA GGCTGGAGGTGTAAGGGGTTAAGCACCGA
 PBS RT scaffold
Spacer Mismatch PAM
Two-sided unpaired student t-test. NC: negative control. The bars represent the mean. Error bars indicate standard deviation (n = 3 independent transfection). Small letters indicate mismatches compared to the pegRNA. Major edited rate corresponds to the frequency of the ‘Edited’ sequence.

## Slide 7
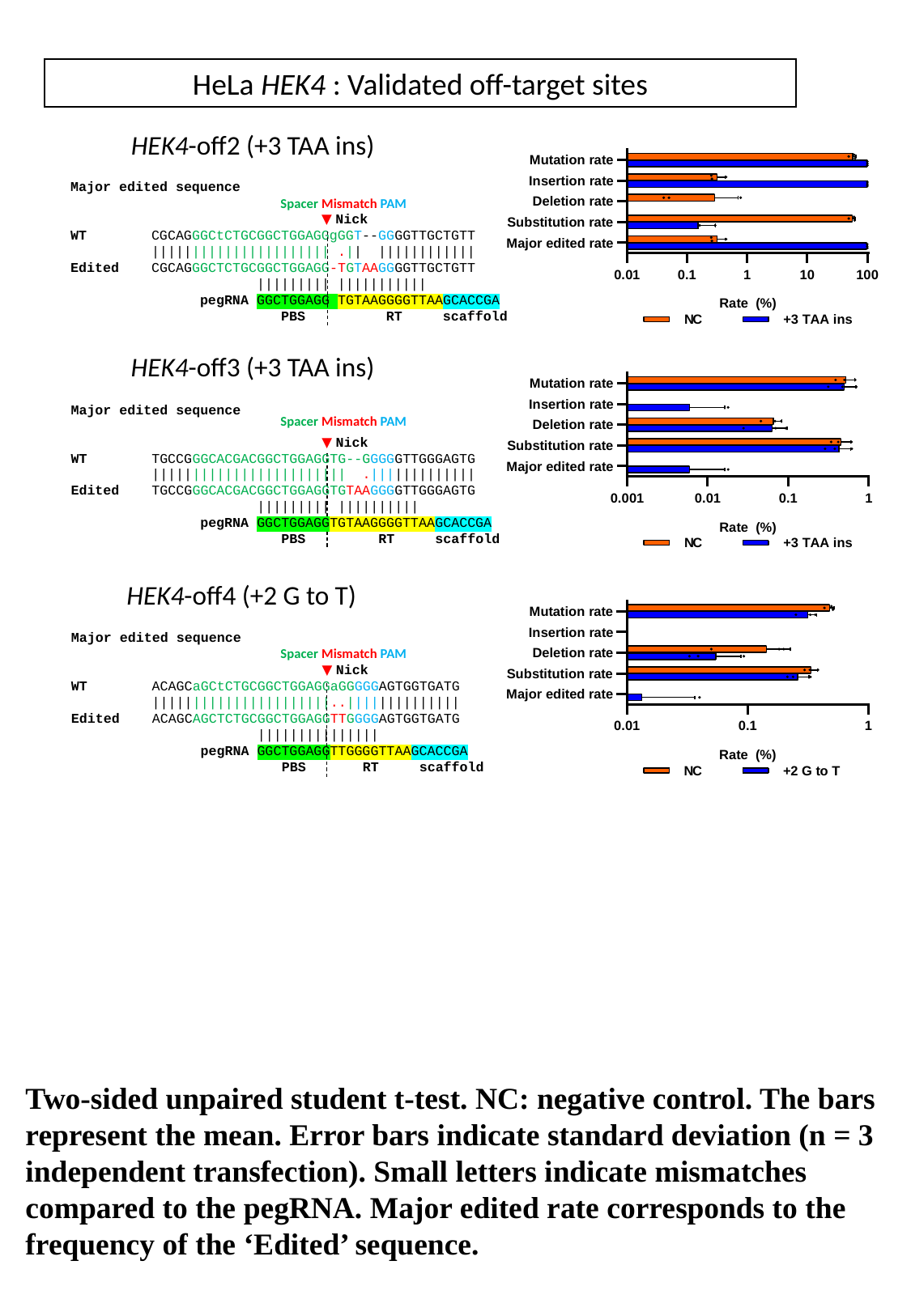

HeLa HEK4 : Validated off-target sites
HEK4-off2 (+3 TAA ins)
Major edited sequence
 ▼ Nick
WT TGCCGGGCACGACGGCTGGAGGTG--GGGGGTTGGGAGTG
 ||||||||||||||||||||||||  .|||||||||||||
Edited TGCCGGGCACGACGGCTGGAGGTGTAAGGGGTTGGGAGTG
 ||||||||| ||||||||||
 pegRNA GGCTGGAGGTGTAAGGGGTTAAGCACCGA
 PBS RT scaffold
Spacer Mismatch PAM
Major edited sequence
 ▼ Nick
WT CGCAGGGCtCTGCGGCTGGAGGgGGT--GGGGTTGCTGTT
 |||||||||||||||||||||| .||  ||||||||||||
Edited CGCAGGGCTCTGCGGCTGGAGG-TGTAAGGGGTTGCTGTT
 ||||||||| |||||||||||
 pegRNA GGCTGGAGG TGTAAGGGGTTAAGCACCGA
 PBS RT scaffold
HEK4-off3 (+3 TAA ins)
Spacer Mismatch PAM
HEK4-off4 (+2 G to T)
Major edited sequence
 ▼ Nick
WT ACAGCaGCtCTGCGGCTGGAGGaGGGGGAGTGGTGATG
 ||||||||||||||||||||||..||||||||||||||
Edited ACAGCAGCTCTGCGGCTGGAGGTTGGGGAGTGGTGATG
 |||||||||||||||
 pegRNA GGCTGGAGGTTGGGGTTAAGCACCGA
 PBS RT scaffold
Spacer Mismatch PAM
Two-sided unpaired student t-test. NC: negative control. The bars represent the mean. Error bars indicate standard deviation (n = 3 independent transfection). Small letters indicate mismatches compared to the pegRNA. Major edited rate corresponds to the frequency of the ‘Edited’ sequence.

## Slide 8
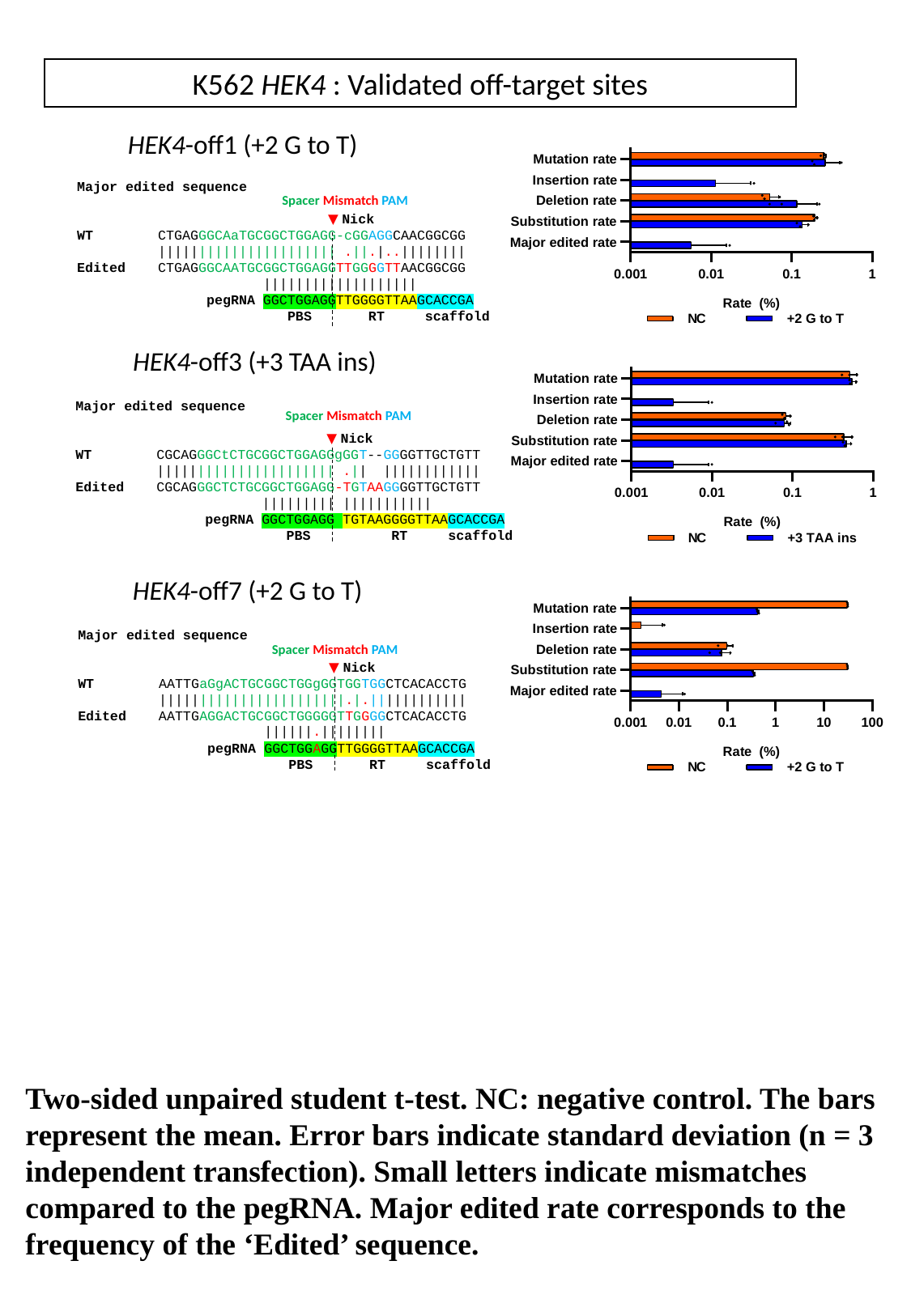

K562 HEK4 : Validated off-target sites
HEK4-off1 (+2 G to T)
Major edited sequence
 ▼ Nick
WT CTGAGGGCAaTGCGGCTGGAGG-cGGAGGCAACGGCGG
 |||||||||||||||||||||| .||.|..||||||||
Edited CTGAGGGCAATGCGGCTGGAGGTTGGGGTTAACGGCGG
 |||||||||||||||||||
 pegRNA GGCTGGAGGTTGGGGTTAAGCACCGA
 PBS RT scaffold
Spacer Mismatch PAM
HEK4-off3 (+3 TAA ins)
Major edited sequence
 ▼ Nick
WT CGCAGGGCtCTGCGGCTGGAGGgGGT--GGGGTTGCTGTT
 |||||||||||||||||||||| .||  ||||||||||||
Edited CGCAGGGCTCTGCGGCTGGAGG-TGTAAGGGGTTGCTGTT
 ||||||||| |||||||||||
 pegRNA GGCTGGAGG TGTAAGGGGTTAAGCACCGA
 PBS RT scaffold
Spacer Mismatch PAM
HEK4-off7 (+2 G to T)
Major edited sequence
 ▼ Nick
WT AATTGaGgACTGCGGCTGGgGGTGGTGGCTCACACCTG
 |||||||||||||||||||||||.|.||||||||||||
Edited AATTGAGGACTGCGGCTGGGGGTTGGGGCTCACACCTG
 ||||||.||||||||
 pegRNA GGCTGGAGGTTGGGGTTAAGCACCGA
 PBS RT scaffold
Spacer Mismatch PAM
Two-sided unpaired student t-test. NC: negative control. The bars represent the mean. Error bars indicate standard deviation (n = 3 independent transfection). Small letters indicate mismatches compared to the pegRNA. Major edited rate corresponds to the frequency of the ‘Edited’ sequence.

## Slide 9
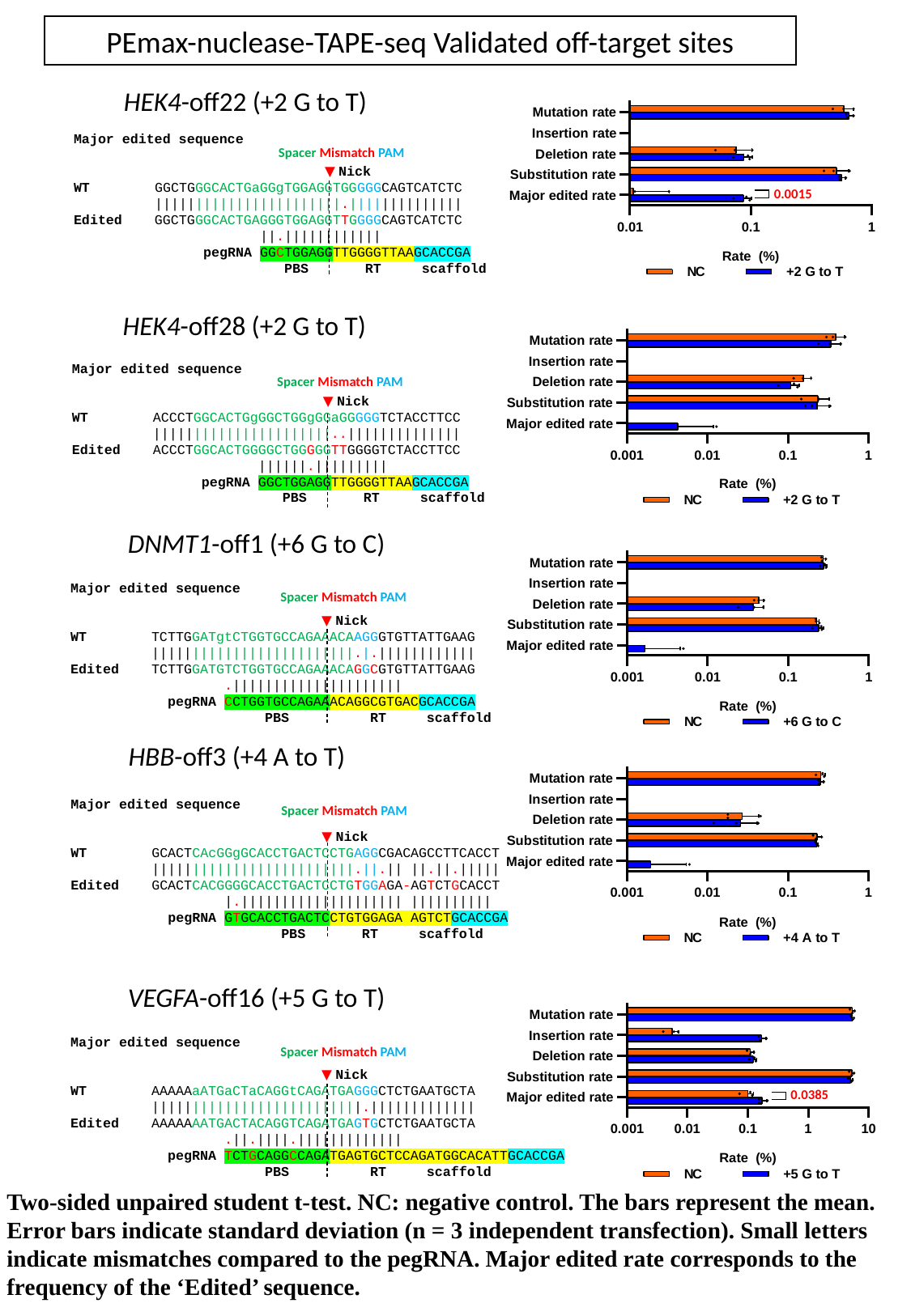

PEmax-nuclease-TAPE-seq Validated off-target sites
0.0015
HEK4-off22 (+2 G to T)
Major edited sequence
 ▼ Nick
WT GGCTGGGCACTGaGGgTGGAGGTGGGGGCAGTCATCTC
 |||||||||||||||||||||||.||||||||||||||
Edited GGCTGGGCACTGAGGGTGGAGGTTGGGGCAGTCATCTC
 ||.||||||||||||
 pegRNA GGCTGGAGGTTGGGGTTAAGCACCGA
 PBS RT scaffold
Spacer Mismatch PAM
HEK4-off28 (+2 G to T)
Major edited sequence
 ▼ Nick
WT ACCCTGGCACTGgGGCTGGgGGaGGGGGTCTACCTTCC
 ||||||||||||||||||||||..||||||||||||||
Edited ACCCTGGCACTGGGGCTGGGGGTTGGGGTCTACCTTCC
 ||||||.|||||||||
 pegRNA GGCTGGAGGTTGGGGTTAAGCACCGA
 PBS RT scaffold
Spacer Mismatch PAM
DNMT1-off1 (+6 G to C)
Major edited sequence
 ▼ Nick
WT TCTTGGATgtCTGGTGCCAGAAACAAGGGTGTTATTGAAG
 |||||||||||||||||||||||||.|.||||||||||||
Edited TCTTGGATGTCTGGTGCCAGAAACAGGCGTGTTATTGAAG
 .|||||||||||||||||||||
 pegRNA CCTGGTGCCAGAAACAGGCGTGACGCACCGA
 PBS RT scaffold
Spacer Mismatch PAM
HBB-off3 (+4 A to T)
Spacer Mismatch PAM
Major edited sequence
 ▼ Nick
WT GCACTCAcGGgGCACCTGACTCCTGAGGCGACAGCCTTCACCT
 |||||||||||||||||||||||||.||.|| ||.||.|||||
Edited GCACTCACGGGGCACCTGACTCCTGTGGAGA-AGTCTGCACCT
 |.|||||||||||||||||||| ||||||||||
 pegRNA GTGCACCTGACTCCTGTGGAGA AGTCTGCACCGA
 PBS RT scaffold
VEGFA-off16 (+5 G to T)
Major edited sequence
 ▼ Nick
WT AAAAAaATGaCTaCAGGtCAGATGAGGGCTCTGAATGCTA
 ||||||||||||||||||||||||||.|||||||||||||
Edited AAAAAAATGACTACAGGTCAGATGAGTGCTCTGAATGCTA
 .||.||||.|||||||||||||
 pegRNA TCTGCAGGCCAGATGAGTGCTCCAGATGGCACATTGCACCGA
 PBS RT scaffold
Spacer Mismatch PAM
0.0385
Two-sided unpaired student t-test. NC: negative control. The bars represent the mean. Error bars indicate standard deviation (n = 3 independent transfection). Small letters indicate mismatches compared to the pegRNA. Major edited rate corresponds to the frequency of the ‘Edited’ sequence.
