# Supplementary material for: TAPE-seq is a cell-based method for predicting genome-wide off-target effects of prime editor
Source: Nat Commun. 2022 Dec 29;13:7975. doi: 10.1038/s41467-022-35743-y (PMC9800413; doi:10.1038/s41467-022-35743-y)
Supplement: Supplementary file 12 — Supplementary Data 10. Vector construct sequence information [file 41467_2022_35743_MOESM12_ESM.docx]

**Supplementary Data 10. Vector construct sequence information**

pRG2-pegRNA

U6 Promoter Stuffer sgRNA scaffold pUC origin Amp resistance

gacgaagactcaattgtcgattagtgaacggatctcgacggtatcgatcacgagactagcctcgagcggccgcccccttcaccgagggcctatttcccatgattccttcatatttgcatatacgatacaaggctgttagagagataattggaattaatttgactgtaaacacaaagatattagtacaaaatacgtgacgtagaaagtaataatttcttgggtagtttgcagttttaaaattatgttttaaaatggactatcatatgcttaccgtaacttgaaagtatttcgatttcttggctttatatatcttgtggaaaggacgaaacaccggagaccacatttccccgaaaagtgccacctgacgtctaagaaaccattattatcatgacattaacctataaaaataggcgtatcacgaggccctttcgtctcgcgcgtttcggtgatgacggtgaaaacctctgacacatgcagctcccggagacggtcacagcttgtctgtaagcggatgccgggagcagacaagcccgtcagggcgcgtcagcgggtgttggcgggtgtcggggctggcttaactatgcggcatcagagcagattgtactgagagtgcaccatatgcggtgtgaaataccgcacagatgcgtaaggagaaaataccgcatcaggcgccattcgccattcaggctgcgcaactgttgggaagggcgatcggtgcgggcctcttcgctattacgccagctggcgaaagggggatgtgctgcaaggcgattaagttgggtaacgccagggttttcccagtcacgacgttgtaaaacgacggccagtgccaagcttgcatgcctgcaggtcgactctagaggatccccgggtaccgagctcgaattcgtaatcatggtcatagctgtttcctgtgtgaaattgttatccgctcacaattccacacaacatacgagccggaagcataaagtgtaaagcctggggtgcctaatagatccggtctccgttttagagctagaaatagcaagttaaaataaggctagtccgttatcaacttgaaaaagtggcaccgagtcggtgcggagacgtgtctgtaagcggatgccgggagcagacaagcccgtcagggcgcgtcagcgggtgttggcgggtgtcggggctggcttaactatgcggcatcagagcagattgtactgagagtgcaccatatgcggtgtgaaataccgcacagatgcgtaaggagaaaataccgcatcaggcgccattcgccattcaggctgcgcaactgttgggaagggcgatcggtgcgggcctcttcgctattacgccagctggcgaaagggggatgtgctgcaaggcgattaagttgggtaacgccagggttttcccagtcacgacgttgtaaaacgacggccagtgccaagcttgcatgcctgcaggtcgactctagaggatccccgggtaccgagctcgaattcgtaatcatggtcatagctgtttcctgtgtgaaattgttatccgctcacaattccacacaacatacgagccggcgtctccctagattcgcgatgtacgggccagatatacgcgttgacattgattattgactagttgtcttcctgcattaatgaatcggccaacgcgcggggagaggcggtttgcgtattgggcgctcttccgcttcctcgctcactgactcgctgcgctcggtcgttcggctgcggcgagcggtatcagctcactcaaaggcggtaatacggttatccacagaatcaggggataacgcaggaaagaacatgtgagcaaaaggccagcaaaaggccaggaaccgtaaaaaggccgcgttgctggcgtttttccataggctccgcccccctgacgagcatcacaaaaatcgacgctcaagtcagaggtggcgaaacccgacaggactataaagataccaggcgtttccccctggaagctccctcgtgcgctctcctgttccgaccctgccgcttaccggatacctgtccgcctttctcccttcgggaagcgtggcgctttctcaatgctcacgctgtaggtatctcagttcggtgtaggtcgttcgctccaagctgggctgtgtgcacgaaccccccgttcagcccgaccgctgcgccttatccggtaactatcgtcttgagtccaacccggtaagacacgacttatcgccactggcagcagccactggtaacaggattagcagagcgaggtatgtaggcggtgctacagagttcttgaagtggtggcctaactacggctacactagaaggacagtatttggtatctgcgctctgctgaagccagttaccttcggaaaaagagttggtagctcttgatccggcaaacaaaccaccgctggtagcggtggtttttttgtttgcaagcagcagattacgcgcagaaaaaaaggatctcaagaagatcctttgatcttttctacggggtctgacgctcagtggaacgaaaactcacgttaagggattttggtcatgagattatcaaaaaggatcttcacctagatccttttaaattaaaaatgaagttttaaatcaatctaaagtatatatgagtaaacttggtctgacagttaccaatgcttaatcagtgaggcacctatctcagcgatctgtctatttcgttcatccatagttgcctgactccccgtcgtgtagataactacgatacgggagggcttaccatctggccccagtgctgcaatgataccgcgagatccacgctcaccggctccagatttatcagcaataaaccagccagccggaagggccgagcgcagaagtggtcctgcaactttatccgcctccatccagtctattaattgttgccgggaagctagagtaagtagttcgccagttaatagtttgcgcaacgttgttgccattgctacaggcatcgtggtgtcacgctcgtcgtttggtatggcttcattcagctccggttcccaacgatcaaggcgagttacatgatcccccatgttgtgcaaaaaagcggttagctccttcggtcctccgatcgttgtcagaagtaagttggccgcagtgttatcactcatggttatggcagcactgcataattctcttactgtcatgccatccgtaagatgcttttctgtgactggtgagtactcaaccaagtcattctgagaatagtgtatgcggcgaccgagttgctcttgcccggcgtcaatacgggataataccgcgccacatagcagaactttaaaagtgctcatcattggaaaacgttcttcggggcgaaaactctcaaggatcttaccgctgttgagatccagttcgatgtaacccactcgtgcacccaactgatcttcagcatcttttactttcaccagcgtttctgggtgagcaaaaacaggaaggcaaaatgccgcaaaaaagggaataagggcgacacggaaatgttgaatactcatactcttcctttttcaatattattgaagcatttatcagggttattgtctcatgagcggatacatatttgaatgtatttagaaaaataaacaaataggggttccgcgcacatttccccgaaaagtgccacctgacgtc

pAllin1-PE2. ITR, inverted terminal repeat.

pegRNA U6 Promoter CMV Promoter EF1alpha Promoter Puromycin resistance ITR

cgatgtacgggccagatatacgcgcgtatatctggcccgtacatcgcgaatctagAAAAAANNNNNNNNNNNNNNNNNNNNNNNNNNNNNgcaccgactcggtgccactttttcaagttgataacggactagccttattttaacttgctatttctagctctaaaacNNNNNNNNNNNNNNNNNNNcggtgtttcgtcctttccacaagatatataaagccaagaaatcgaaatactttcaagttacggtaagcatatgatagtccattttaaaacataattttaaaactgcaaactacccaagaaattattactttctacgtcacgtattttgtactaatatctttgtgtttacagtcaaattaattccaattatctctctaacagccttgtatcgtatatgcaaatatgaaggaatcatgggaaataggccctccgcgttgacattgattattgactagttattaatagtaatcaattacggggtcattagttcatagcccatatatggagttccgcgttacataacttacggtaaatggcccgcctggctgaccgcccaacgacccccgcccattgacgtcaataatgacgtatgttcccatagtaacgccaatagggactttccattgacgtcaatgggtggagtatttacggtaaactgcccacttggcagtacatcaagtgtatcatatgccaagtacgccccctattgacgtcaatgacggtaaatggcccgcctggcattatgcccagtacatgaccttatgggactttcctacttggcagtacatctacgtattagtcatcgctattaccatggtgatgcggttttggcagtacatcaatgggcgtggatagcggtttgactcacggggatttccaagtctccaccccattgacgtcaatgggagtttgttttggcaccaaaatcaacgggactttccaaaatgtcgtaacaactccgccccattgacgcaaatgggcggtaggcgtgtacggtgggaggtctatataagcagagctggtttagtgaaccgtcagatccgctagagatccgcggccgctaatacgactcactatagggagagccgccaccatgaaacggacagccgacggaagcgagttcgagtcaccaaagaagaagcggaaagtcgacaagaagtacagcatcggcctggacatcggcaccaactctgtgggctgggccgtgatcaccgacgagtacaaggtgcccagcaagaaattcaaggtgctgggcaacaccgaccggcacagcatcaagaagaacctgatcggagccctgctgttcgacagcggcgaaacagccgaggccacccggctgaagagaaccgccagaagaagatacaccagacggaagaaccggatctgctatctgcaagagatcttcagcaacgagatggccaaggtggacgacagcttcttccacagactggaagagtccttcctggtggaagaggataagaagcacgagcggcaccccatcttcggcaacatcgtggacgaggtggcctaccacgagaagtaccccaccatctaccacctgagaaagaaactggtggacagcaccgacaaggccgacctgcggctgatctatctggccctggcccacatgatcaagttccggggccacttcctgatcgagggcgacctgaaccccgacaacagcgacgtggacaagctgttcatccagctggtgcagacctacaaccagctgttcgaggaaaaccccatcaacgccagcggcgtggacgccaaggccatcctgtctgccagactgagcaagagcagacggctggaaaatctgatcgcccagctgcccggcgagaagaagaatggcctgttcggaaacctgattgccctgagcctgggcctgacccccaacttcaagagcaacttcgacctggccgaggatgccaaactgcagctgagcaaggacacctacgacgacgacctggacaacctgctggcccagatcggcgaccagtacgccgacctgtttctggccgccaagaacctgtccgacgccatcctgctgagcgacatcctgagagtgaacaccgagatcaccaaggcccccctgagcgcctctatgatcaagagatacgacgagcaccaccaggacctgaccctgctgaaagctctcgtgcggcagcagctgcctgagaagtacaaagagattttcttcgaccagagcaagaacggctacgccggctacattgacggcggagccagccaggaagagttctacaagttcatcaagcccatcctggaaaagatggacggcaccgaggaactgctcgtgaagctgaacagagaggacctgctgcggaagcagcggaccttcgacaacggcagcatcccccaccagatccacctgggagagctgcacgccattctgcggcggcaggaagatttttacccattcctgaaggacaaccgggaaaagatcgagaagatcctgaccttccgcatcccctactacgtgggccctctggccaggggaaacagcagattcgcctggatgaccagaaagagcgaggaaaccatcaccccctggaacttcgaggaagtggtggacaagggcgcttccgcccagagcttcatcgagcggatgaccaacttcgataagaacctgcccaacgagaaggtgctgcccaagcacagcctgctgtacgagtacttcaccgtgtataacgagctgaccaaagtgaaatacgtgaccgagggaatgagaaagcccgccttcctgagcggcgagcagaaaaaggccatcgtggacctgctgttcaagaccaaccggaaagtgaccgtgaagcagctgaaagaggactacttcaagaaaatcgagtgcttcgactccgtggaaatctccggcgtggaagatcggttcaacgcctccctgggcacataccacgatctgctgaaaattatcaaggacaaggacttcctggacaatgaggaaaacgaggacattctggaagatatcgtgctgaccctgacactgtttgaggacagagagatgatcgaggaacggctgaaaacctatgcccacctgttcgacgacaaagtgatgaagcagctgaagcggcggagatacaccggctggggcaggctgagccggaagctgatcaacggcatccgggacaagcagtccggcaagacaatcctggatttcctgaagtccgacggcttcgccaacagaaacttcatgcagctgatccacgacgacagcctgacctttaaagaggacatccagaaagcccaggtgtccggccagggcgatagcctgcacgagcacattgccaatctggccggcagccccgccattaagaagggcatcctgcagacagtgaaggtggtggacgagctcgtgaaagtgatgggccggcacaagcccgagaacatcgtgatcgaaatggccagagagaaccagaccacccagaagggacagaagaacagccgcgagagaatgaagcggatcgaagagggcatcaaagagctgggcagccagatcctgaaagaacaccccgtggaaaacacccagctgcagaacgagaagctgtacctgtactacctgcagaatgggcgggatatgtacgtggaccaggaactggacatcaaccggctgtccgactacgatgtggacgctatcgtgcctcagagctttctgaaggacgactccatcgacaacaaggtgctgaccagaagcgacaagaaccggggcaagagcgacaacgtgccctccgaagaggtcgtgaagaagatgaagaactactggcggcagctgctgaacgccaagctgattacccagagaaagttcgacaatctgaccaaggccgagagaggcggcctgagcgaactggataaggccggcttcatcaagagacagctggtggaaacccggcagatcacaaagcacgtggcacagatcctggactcccggatgaacactaagtacgacgagaatgacaagctgatccgggaagtgaaagtgatcaccctgaagtccaagctggtgtccgatttccggaaggatttccagttttacaaagtgcgcgagatcaacaactaccaccacgcccacgacgcctacctgaacgccgtcgtgggaaccgccctgatcaaaaagtaccctaagctggaaagcgagttcgtgtacggcgactacaaggtgtacgacgtgcggaagatgatcgccaagagcgagcaggaaatcggcaaggctaccgccaagtacttcttctacagcaacatcatgaactttttcaagaccgagattaccctggccaacggcgagatccggaagcggcctctgatcgagacaaacggcgaaaccggggagatcgtgtgggataagggccgggattttgccaccgtgcggaaagtgctgagcatgccccaagtgaatatcgtgaaaaagaccgaggtgcagacaggcggcttcagcaaagagtctatcctgcccaagaggaacagcgataagctgatcgccagaaagaaggactgggaccctaagaagtacggcggcttcgacagccccaccgtggcctattctgtgctggtggtggccaaagtggaaaagggcaagtccaagaaactgaagagtgtgaaagagctgctggggatcaccatcatggaaagaagcagcttcgagaagaatcccatcgactttctggaagccaagggctacaaagaagtgaaaaaggacctgatcatcaagctgcctaagtactccctgttcgagctggaaaacggccggaagagaatgctggcctctgccggcgaactgcagaagggaaacgaactggccctgccctccaaatatgtgaacttcctgtacctggccagccactatgagaagctgaagggctcccccgaggataatgagcagaaacagctgtttgtggaacagcacaagcactacctggacgagatcatcgagcagatcagcgagttctccaagagagtgatcctggccgacgctaatctggacaaagtgctgtccgcctacaacaagcaccgggataagcccatcagagagcaggccgagaatatcatccacctgtttaccctgaccaatctgggagcccctgccgccttcaagtactttgacaccaccatcgaccggaagaggtacaccagcaccaaagaggtgctggacgccaccctgatccaccagagcatcaccggcctgtacgagacacggatcgacctgtctcagctgggaggtgactctggaggatctagcggaggatcctctggcagcgagacaccaggaacaagcgagtcagcaacaccagagagcagtggcggcagcagcggcggcagcagcaccctaaatatagaagatgagtatcggctacatgagacctcaaaagagccagatgtttctctagggtccacatggctgtctgattttcctcaggcctgggcggaaaccgggggcatgggactggcagttcgccaagctcctctgatcatacctctgaaagcaacctctacccccgtgtccataaaacaataccccatgtcacaagaagccagactggggatcaagccccacatacagagactgttggaccagggaatactggtaccctgccagtccccctggaacacgcccctgctacccgttaagaaaccagggactaatgattataggcctgtccaggatctgagagaagtcaacaagcgggtggaagacatccaccccaccgtgcccaacccttacaacctcttgagcgggctcccaccgtcccaccagtggtacactgtgcttgatttaaaggatgcctttttctgcctgagactccaccccaccagtcagcctctcttcgcctttgagtggagagatccagagatgggaatctcaggacaattgacctggaccagactcccacagggtttcaaaaacagtcccaccctgtttaatgaggcactgcacagagacctagcagacttccggatccagcacccagacttgatcctgctacagtacgtggatgacttactgctggccgccacttctgagctagactgccaacaaggtactcgggccctgttacaaaccctagggaacctcgggtatcgggcctcggccaagaaagcccaaatttgccagaaacaggtcaagtatctggggtatcttctaaaagagggtcagagatggctgactgaggccagaaaagagactgtgatggggcagcctactccgaagacccctcgacaactaagggagttcctagggaaggcaggcttctgtcgcctcttcatccctgggtttgcagaaatggcagcccccctgtaccctctcaccaaaccggggactctgtttaattggggcccagaccaacaaaaggcctatcaagaaatcaagcaagctcttctaactgccccagccctggggttgccagatttgactaagccctttgaactctttgtcgacgagaagcagggctacgccaaaggtgtcctaacgcaaaaactgggaccttggcgtcggccggtggcctacctgtccaaaaagctagacccagtagcagctgggtggcccccttgcctacggatggtagcagccattgccgtactgacaaaggatgcaggcaagctaaccatgggacagccactagtcattctggccccccatgcagtagaggcactagtcaaacaaccccccgaccgctggctttccaacgcccggatgactcactatcaggccttgcttttggacacggaccgggtccagttcggaccggtggtagccctgaacccggctacgctgctcccactgcctgaggaagggctgcaacacaactgccttgatatcctggccgaagcccacggaacccgacccgacctaacggaccagccgctcccagacgccgaccacacctggtacacggatggaagcagtctcttacaagagggacagcgtaaggcgggagctgcggtgaccaccgagaccgaggtaatctgggctaaagccctgccagccgggacatccgctcagcgggctgaactgatagcactcacccaggccctaaagatggcagaaggtaagaagctaaatgtttatactgatagccgttatgcttttgctactgcccatatccatggagaaatatacagaaggcgtgggtggctcacatcagaaggcaaagagatcaaaaataaagacgagatcttggccctactaaaagccctctttctgcccaaaagacttagcataatccattgtccaggacatcaaaagggacacagcgccgaggctagaggcaaccggatggctgaccaagcggcccgaaaggcagccatcacagagactccagacacctctaccctcctcatagaaaattcatcaccctctggcggctcaaaaagaaccgccgacggcagcgaattcgagcccaagaagaagaggaaagtctaaccggtcatcatcaccatcaccattgagttttacccctacgacgtgcccgactacgcctaataactcgagcatgcatctagagggccctattctatagtgtcacctaaatgctagagctcgctgatcagcctcgactgtgccttctagttgccagccatctgttgtttgcccctcccccgtgccttccttgaccctggaaggtgccactcccactgtcctttcctaataaaatgaggaaattgcatcgcattgtctgagtaggtgtcattctattctggggggtggggtggggcaggacagcaagggggaggattgggaagacaatagcaggcatgctggggaggatctgcgatcgctccggtgcccgtcagtgggcagagcgcacatcgcccacagtccccgagaagttggggggaggggtcggcaattgaacgggtgcctagagaaggtggcgcggggtaaactgggaaagtgatgtcgtgtactggctccgcctttttcccgagggtgggggagaaccgtatataagtgcagtagtcgccgtgaacgttctttttcgcaacgggtttgccgccagaacacagctgaagcttcgaggggctcgcatctctccttcacgcgcccgccgccctacctgaggccgccatccacgccggttgagtcgcgttctgccgcctcccgcctgtggtgcctcctgaactgcgtccgccgtctaggtaagtttaaagctcaggtcgagaccgggcctttgtccggcgctcccttggagcctacctagactcagccggctctccacgctttgcctgaccctgcttgctcaactctacgtctttgtttcgttttctgttctgcgccgttacagatccaagctgtgaccggcgcctacATGACCGAGTACAAGCCCACGGTGCGCCTCGCCACCCGCGACGACGTCCCCAGGGCCGTACGCACCCTCGCCGCCGCGTTCGCCGACTACCCCGCCACGCGCCACACCGTCGATCCAGACCGCCACATCGAGCGGGTCACCGAGCTGCAAGAACTCTTCCTCACGCGCGTCGGGCTCGACATCGGCAAGGTGTGGGTCGCGGACGACGGCGCCGCGGTGGCGGTCTGGACCACGCCGGAGAGCGTCGAAGCGGGGGCGGTGTTCGCCGAGATCGGCCCGCGCATGGCCGAGTTGAGCGGTTCCCGGCTGGCCGCGCAGCAACAGATGGAAGGTCTCCTGGCGCCGCACCGGCCCAAGGAGCCCGCGTGGTTCCTGGCCACCGTCGGCGTCTCGCCCGACCACCAGGGCAAGGGTCTGGGCAGCGCCGTCGTGCTCCCCGGAGTGGAGGCGGCCGAGCGCGCCGGGGTGCCCGCCTTCCTGGAGACCTCCGCGCCCCGCAACCTCCCCTTCTACGAGCGGCTCGGCTTCACCGTCACCGCCGACGTCGAGGTGCCCGAAGGACCGCGCACCTGGTGCATGACCCGCAAGCCCGGTGCCTGAgctagaagctcgctttcttgctgtccaatttctattaaaggttcctttgttccctaagtccaactactaaactgggggatattatgaagggccttgagcatctggattctgcctaataaaaaacatttattttcattgcaatgatgtatttaaattatttctgaatattttactaaaaagggaatgtgggaggtcagtgcatttaaaacataaagaaatgaagagctagttcaaaccttgggaaaatacactatatcttaaactccatgaaagaaggtgaggctgcaaacagctaatgcacattggcaacagcccctgatgcctatgccttattcatccctcagaaaaggattcaagtagaggcttgatttggaggttaaagttttgctatgctgtattttagaattccataccacatttgtagaggttttacttgctttaaaaaacctcccacacctccccctgaacctgaaacataaaatgaatgcaattgttgttgttttaccaatgcttaatcagtgaggcacctatctcagcgatctgtctatttcgttcatccatagttgcctgactccccgtcgtgtagataactacgatacgggagggcttaccatctggccccagtgctgcaatgataccgcgagacccacgctcaccggctccagatttatcagcaataaaccagccagccggaagggccgagcgcagaagtggtcctgcaactttatccgcctccatccagtctattaattgttgccgggaagctagagtaagtagttcgccagttaatagtttgcgcaacgttgttgccattgctacaggcatcgtggtgtcacgctcgtcgtttggtatggcttcattcagctccggttcccaacgatcaaggcgagttacatgatcccccatgttgtgcaaaaaagcggttagctccttcggtcctccgatcgttgtcagaagtaagttggccgcagtgttatcactcatggttatggcagcactgcataattctcttactgtcatgccatccgtaagatgcttttctgtgactggtgagtactcaaccaagtcattctgagaatagtgtatgcggcgaccgagttgctcttgcccggcgtcaatacgggataataccgcgccacatagcagaactttaaaagtgctcatcattggaaaacgttcttcggggcgaaaactctcaaggatcttaccgctgttgagatccagttcgatgtaacccactcgtgcacccaactgatcttcagcatcttttactttcaccagcgtttctgggtgagcaaaaacaggaaggcaaaatgccgcaaaaaagggaataagggcgacacggaaatgttgaatactcatactcttcctttttcaatattattgaagcatttatcagggttattgtctcatgagcggatacatatttgaatgtatttagaaaaataaacaaataggggttccgcgaacttgtttattgcagcttataatggttacaaataaagcaatagcatcacaaatttcacaaataaagcatttttttcactgcattctagttgtggtttgtccaaactcatcaatgtatcttatcatgtctggccagctagccaacaagctcgtcatcgctttgcagaagagcagagaggatatgctcatcgtctaaagaactacccattttattatatattagtcacgatatctataacaagaaaatatatatataataagttatcacgtaagtagaacatgaaataacaatataattatcgtatgagttaaatcttaaaagtcacgtaaaagataatcatgcgtcattttgactcacgcggtcgttatagttcaaaatcagtgacacttaccgcattgacaagcacgcctcacgggagctccaagcggcgactgagatgtcctaaatgcacagcgacggattcgcgctatttagaaagagagagcaatatttcaagaatgcatgcgtcaattttacgcagactatctttctagggttaaaaaagatttgcgctttactcgacctaaactttaaacacgtcatagaatcttcgtttgacaaaaaccacattgtggccaagctgtgtgacgcgacgcgcgctaaagaatggcaaaccaagtcgcgcgaggtacccagcttttgttccctttagtgagggttaattccgagcttggcgtaatcatggtcatagctgtttcctgtgtgaaattgttatccgctcacaattccacacaacatacgagccggaagcataaagtgtaaagcctggggtgcctaatgagtgagctaactcacattaattgcgttgcgctcactgcccgctttccagtcgggaaacctgtcgtgccagctgcattaatgaatcggccaacgcgcggggagaggcggtttgcgtattgggcgctcttccgcttcctcgctcactgactcgctgcgctcggtcgttcggctgcggcgagcggtatcagctcactcaaaggcggtaatacggttatccacagaatcaggggataacgcaggaaagaacatgtgagcaaaaggccagcaaaaggccaggaaccgtaaaaaggccgcgttgctggcgtttttccataggctccgcccccctgacgagcatcacaaaaatcgacgctcaagtcagaggtggcgaaacccgacaggactataaagataccaggcgtttccccctggaagctccctcgtgcgctctcctgttccgaccctgccgcttaccggatacctgtccgcctttctcccttcgggaagcgtggcgctttctcatagctcacgctgtaggtatctcagttcggtgtaggtcgttcgctccaagctgggctgtgtgcacgaaccccccgttcagcccgaccgctgcgccttatccggtaactatcgtcttgagtccaacccggtaagacacgacttatcgccactggcagcagccactggtaacaggattagcagagcgaggtatgtaggcggtgctacagagttcttgaagtggtggcctaactacggctacactagaaggacagtatttggtatctgcgctctgctgaagccagttaccttcggaaaaagagttggtagctcttgatccggcaaacaaaccaccgctggtagcggtggtttttttgtttgcaagcagcagattacgcgcagaaaaaaaggatctcaagaagatcctttgatcttttctacggggtctgacgctcagtggaacgaaaactcacgttaagggattttggtcatgattaaccctagaaagataatcatattgtgacgtacgttaaagataatcatgcgtaaaattgacgcatgtgttttatcggtctgtatatcgaggtttatttattaatttgaatagatattaagttttattatatttacacttacatactaataataaattcaacaaacaatttatttatgtttatttatttattaaaaaaaaacaaaaactcaaaatttcttctataaagtaacaaaacttttaaacattctctcttttacaaaaataaacttattttgtactttaaaaacagtcatgttgtattataaaataagtaattagcttaacctatacataatagaaacaaattatacttattagtcagtcagaaacaaCTTTGGCACATATCAATATTATGCTCTCGTTAATTAA

piggy-PE2

CMV Promoter EF1alpha Promoter Puromycin resistance ITR

CGATGTACGGGCCAGATATACGCGTTGACATTGATTATTGACTAGTTATTAATAGTAATCAATTACGGGGTCATTAGTTCATAGCCCATATATGGAGTTCCGCGTTACATAACTTACGGTAAATGGCCCGCCTGGCTGACCGCCCAACGACCCCCGCCCATTGACGTCAATAATGACGTATGTTCCCATAGTAACGCCAATAGGGACTTTCCATTGACGTCAATGGGTGGAGTATTTACGGTAAACTGCCCACTTGGCAGTACATCAAGTGTATCATATGCCAAGTACGCCCCCTATTGACGTCAATGACGGTAAATGGCCCGCCTGGCATTATGCCCAGTACATGACCTTATGGGACTTTCCTACTTGGCAGTACATCTACGTATTAGTCATCGCTATTACCATGGTGATGCGGTTTTGGCAGTACATCAATGGGCGTGGATAGCGGTTTGACTCACGGGGATTTCCAAGTCTCCACCCCATTGACGTCAATGGGAGTTTGTTTTGGCACCAAAATCAACGGGACTTTCCAAAATGTCGTAACAACTCCGCCCCATTGACGCAAATGGGCGGTAGGCGTGTACGGTGGGAGGTCTATATAAGCAGAGCTGGTTTAGTGAACCGTCAGATCCGCTAGAGATCCGCGGCCGCTAATACGACTCACTATAGGGAGAGCCGCCACCATGAAACGGACAGCCGACGGAAGCGAGTTCGAGTCACCAAAGAAGAAGCGGAAAGTCGACAAGAAGTACAGCATCGGCCTGGACATCGGCACCAACTCTGTGGGCTGGGCCGTGATCACCGACGAGTACAAGGTGCCCAGCAAGAAATTCAAGGTGCTGGGCAACACCGACCGGCACAGCATCAAGAAGAACCTGATCGGAGCCCTGCTGTTCGACAGCGGCGAAACAGCCGAGGCCACCCGGCTGAAGAGAACCGCCAGAAGAAGATACACCAGACGGAAGAACCGGATCTGCTATCTGCAAGAGATCTTCAGCAACGAGATGGCCAAGGTGGACGACAGCTTCTTCCACAGACTGGAAGAGTCCTTCCTGGTGGAAGAGGATAAGAAGCACGAGCGGCACCCCATCTTCGGCAACATCGTGGACGAGGTGGCCTACCACGAGAAGTACCCCACCATCTACCACCTGAGAAAGAAACTGGTGGACAGCACCGACAAGGCCGACCTGCGGCTGATCTATCTGGCCCTGGCCCACATGATCAAGTTCCGGGGCCACTTCCTGATCGAGGGCGACCTGAACCCCGACAACAGCGACGTGGACAAGCTGTTCATCCAGCTGGTGCAGACCTACAACCAGCTGTTCGAGGAAAACCCCATCAACGCCAGCGGCGTGGACGCCAAGGCCATCCTGTCTGCCAGACTGAGCAAGAGCAGACGGCTGGAAAATCTGATCGCCCAGCTGCCCGGCGAGAAGAAGAATGGCCTGTTCGGAAACCTGATTGCCCTGAGCCTGGGCCTGACCCCCAACTTCAAGAGCAACTTCGACCTGGCCGAGGATGCCAAACTGCAGCTGAGCAAGGACACCTACGACGACGACCTGGACAACCTGCTGGCCCAGATCGGCGACCAGTACGCCGACCTGTTTCTGGCCGCCAAGAACCTGTCCGACGCCATCCTGCTGAGCGACATCCTGAGAGTGAACACCGAGATCACCAAGGCCCCCCTGAGCGCCTCTATGATCAAGAGATACGACGAGCACCACCAGGACCTGACCCTGCTGAAAGCTCTCGTGCGGCAGCAGCTGCCTGAGAAGTACAAAGAGATTTTCTTCGACCAGAGCAAGAACGGCTACGCCGGCTACATTGACGGCGGAGCCAGCCAGGAAGAGTTCTACAAGTTCATCAAGCCCATCCTGGAAAAGATGGACGGCACCGAGGAACTGCTCGTGAAGCTGAACAGAGAGGACCTGCTGCGGAAGCAGCGGACCTTCGACAACGGCAGCATCCCCCACCAGATCCACCTGGGAGAGCTGCACGCCATTCTGCGGCGGCAGGAAGATTTTTACCCATTCCTGAAGGACAACCGGGAAAAGATCGAGAAGATCCTGACCTTCCGCATCCCCTACTACGTGGGCCCTCTGGCCAGGGGAAACAGCAGATTCGCCTGGATGACCAGAAAGAGCGAGGAAACCATCACCCCCTGGAACTTCGAGGAAGTGGTGGACAAGGGCGCTTCCGCCCAGAGCTTCATCGAGCGGATGACCAACTTCGATAAGAACCTGCCCAACGAGAAGGTGCTGCCCAAGCACAGCCTGCTGTACGAGTACTTCACCGTGTATAACGAGCTGACCAAAGTGAAATACGTGACCGAGGGAATGAGAAAGCCCGCCTTCCTGAGCGGCGAGCAGAAAAAGGCCATCGTGGACCTGCTGTTCAAGACCAACCGGAAAGTGACCGTGAAGCAGCTGAAAGAGGACTACTTCAAGAAAATCGAGTGCTTCGACTCCGTGGAAATCTCCGGCGTGGAAGATCGGTTCAACGCCTCCCTGGGCACATACCACGATCTGCTGAAAATTATCAAGGACAAGGACTTCCTGGACAATGAGGAAAACGAGGACATTCTGGAAGATATCGTGCTGACCCTGACACTGTTTGAGGACAGAGAGATGATCGAGGAACGGCTGAAAACCTATGCCCACCTGTTCGACGACAAAGTGATGAAGCAGCTGAAGCGGCGGAGATACACCGGCTGGGGCAGGCTGAGCCGGAAGCTGATCAACGGCATCCGGGACAAGCAGTCCGGCAAGACAATCCTGGATTTCCTGAAGTCCGACGGCTTCGCCAACAGAAACTTCATGCAGCTGATCCACGACGACAGCCTGACCTTTAAAGAGGACATCCAGAAAGCCCAGGTGTCCGGCCAGGGCGATAGCCTGCACGAGCACATTGCCAATCTGGCCGGCAGCCCCGCCATTAAGAAGGGCATCCTGCAGACAGTGAAGGTGGTGGACGAGCTCGTGAAAGTGATGGGCCGGCACAAGCCCGAGAACATCGTGATCGAAATGGCCAGAGAGAACCAGACCACCCAGAAGGGACAGAAGAACAGCCGCGAGAGAATGAAGCGGATCGAAGAGGGCATCAAAGAGCTGGGCAGCCAGATCCTGAAAGAACACCCCGTGGAAAACACCCAGCTGCAGAACGAGAAGCTGTACCTGTACTACCTGCAGAATGGGCGGGATATGTACGTGGACCAGGAACTGGACATCAACCGGCTGTCCGACTACGATGTGGACGCTATCGTGCCTCAGAGCTTTCTGAAGGACGACTCCATCGACAACAAGGTGCTGACCAGAAGCGACAAGAACCGGGGCAAGAGCGACAACGTGCCCTCCGAAGAGGTCGTGAAGAAGATGAAGAACTACTGGCGGCAGCTGCTGAACGCCAAGCTGATTACCCAGAGAAAGTTCGACAATCTGACCAAGGCCGAGAGAGGCGGCCTGAGCGAACTGGATAAGGCCGGCTTCATCAAGAGACAGCTGGTGGAAACCCGGCAGATCACAAAGCACGTGGCACAGATCCTGGACTCCCGGATGAACACTAAGTACGACGAGAATGACAAGCTGATCCGGGAAGTGAAAGTGATCACCCTGAAGTCCAAGCTGGTGTCCGATTTCCGGAAGGATTTCCAGTTTTACAAAGTGCGCGAGATCAACAACTACCACCACGCCCACGACGCCTACCTGAACGCCGTCGTGGGAACCGCCCTGATCAAAAAGTACCCTAAGCTGGAAAGCGAGTTCGTGTACGGCGACTACAAGGTGTACGACGTGCGGAAGATGATCGCCAAGAGCGAGCAGGAAATCGGCAAGGCTACCGCCAAGTACTTCTTCTACAGCAACATCATGAACTTTTTCAAGACCGAGATTACCCTGGCCAACGGCGAGATCCGGAAGCGGCCTCTGATCGAGACAAACGGCGAAACCGGGGAGATCGTGTGGGATAAGGGCCGGGATTTTGCCACCGTGCGGAAAGTGCTGAGCATGCCCCAAGTGAATATCGTGAAAAAGACCGAGGTGCAGACAGGCGGCTTCAGCAAAGAGTCTATCCTGCCCAAGAGGAACAGCGATAAGCTGATCGCCAGAAAGAAGGACTGGGACCCTAAGAAGTACGGCGGCTTCGACAGCCCCACCGTGGCCTATTCTGTGCTGGTGGTGGCCAAAGTGGAAAAGGGCAAGTCCAAGAAACTGAAGAGTGTGAAAGAGCTGCTGGGGATCACCATCATGGAAAGAAGCAGCTTCGAGAAGAATCCCATCGACTTTCTGGAAGCCAAGGGCTACAAAGAAGTGAAAAAGGACCTGATCATCAAGCTGCCTAAGTACTCCCTGTTCGAGCTGGAAAACGGCCGGAAGAGAATGCTGGCCTCTGCCGGCGAACTGCAGAAGGGAAACGAACTGGCCCTGCCCTCCAAATATGTGAACTTCCTGTACCTGGCCAGCCACTATGAGAAGCTGAAGGGCTCCCCCGAGGATAATGAGCAGAAACAGCTGTTTGTGGAACAGCACAAGCACTACCTGGACGAGATCATCGAGCAGATCAGCGAGTTCTCCAAGAGAGTGATCCTGGCCGACGCTAATCTGGACAAAGTGCTGTCCGCCTACAACAAGCACCGGGATAAGCCCATCAGAGAGCAGGCCGAGAATATCATCCACCTGTTTACCCTGACCAATCTGGGAGCCCCTGCCGCCTTCAAGTACTTTGACACCACCATCGACCGGAAGAGGTACACCAGCACCAAAGAGGTGCTGGACGCCACCCTGATCCACCAGAGCATCACCGGCCTGTACGAGACACGGATCGACCTGTCTCAGCTGGGAGGTGACTCTGGAGGATCTAGCGGAGGATCCTCTGGCAGCGAGACACCAGGAACAAGCGAGTCAGCAACACCAGAGAGCAGTGGCGGCAGCAGCGGCGGCAGCAGCACCCTAAATATAGAAGATGAGTATCGGCTACATGAGACCTCAAAAGAGCCAGATGTTTCTCTAGGGTCCACATGGCTGTCTGATTTTCCTCAGGCCTGGGCGGAAACCGGGGGCATGGGACTGGCAGTTCGCCAAGCTCCTCTGATCATACCTCTGAAAGCAACCTCTACCCCCGTGTCCATAAAACAATACCCCATGTCACAAGAAGCCAGACTGGGGATCAAGCCCCACATACAGAGACTGTTGGACCAGGGAATACTGGTACCCTGCCAGTCCCCCTGGAACACGCCCCTGCTACCCGTTAAGAAACCAGGGACTAATGATTATAGGCCTGTCCAGGATCTGAGAGAAGTCAACAAGCGGGTGGAAGACATCCACCCCACCGTGCCCAACCCTTACAACCTCTTGAGCGGGCTCCCACCGTCCCACCAGTGGTACACTGTGCTTGATTTAAAGGATGCCTTTTTCTGCCTGAGACTCCACCCCACCAGTCAGCCTCTCTTCGCCTTTGAGTGGAGAGATCCAGAGATGGGAATCTCAGGACAATTGACCTGGACCAGACTCCCACAGGGTTTCAAAAACAGTCCCACCCTGTTTAATGAGGCACTGCACAGAGACCTAGCAGACTTCCGGATCCAGCACCCAGACTTGATCCTGCTACAGTACGTGGATGACTTACTGCTGGCCGCCACTTCTGAGCTAGACTGCCAACAAGGTACTCGGGCCCTGTTACAAACCCTAGGGAACCTCGGGTATCGGGCCTCGGCCAAGAAAGCCCAAATTTGCCAGAAACAGGTCAAGTATCTGGGGTATCTTCTAAAAGAGGGTCAGAGATGGCTGACTGAGGCCAGAAAAGAGACTGTGATGGGGCAGCCTACTCCGAAGACCCCTCGACAACTAAGGGAGTTCCTAGGGAAGGCAGGCTTCTGTCGCCTCTTCATCCCTGGGTTTGCAGAAATGGCAGCCCCCCTGTACCCTCTCACCAAACCGGGGACTCTGTTTAATTGGGGCCCAGACCAACAAAAGGCCTATCAAGAAATCAAGCAAGCTCTTCTAACTGCCCCAGCCCTGGGGTTGCCAGATTTGACTAAGCCCTTTGAACTCTTTGTCGACGAGAAGCAGGGCTACGCCAAAGGTGTCCTAACGCAAAAACTGGGACCTTGGCGTCGGCCGGTGGCCTACCTGTCCAAAAAGCTAGACCCAGTAGCAGCTGGGTGGCCCCCTTGCCTACGGATGGTAGCAGCCATTGCCGTACTGACAAAGGATGCAGGCAAGCTAACCATGGGACAGCCACTAGTCATTCTGGCCCCCCATGCAGTAGAGGCACTAGTCAAACAACCCCCCGACCGCTGGCTTTCCAACGCCCGGATGACTCACTATCAGGCCTTGCTTTTGGACACGGACCGGGTCCAGTTCGGACCGGTGGTAGCCCTGAACCCGGCTACGCTGCTCCCACTGCCTGAGGAAGGGCTGCAACACAACTGCCTTGATATCCTGGCCGAAGCCCACGGAACCCGACCCGACCTAACGGACCAGCCGCTCCCAGACGCCGACCACACCTGGTACACGGATGGAAGCAGTCTCTTACAAGAGGGACAGCGTAAGGCGGGAGCTGCGGTGACCACCGAGACCGAGGTAATCTGGGCTAAAGCCCTGCCAGCCGGGACATCCGCTCAGCGGGCTGAACTGATAGCACTCACCCAGGCCCTAAAGATGGCAGAAGGTAAGAAGCTAAATGTTTATACTGATAGCCGTTATGCTTTTGCTACTGCCCATATCCATGGAGAAATATACAGAAGGCGTGGGTGGCTCACATCAGAAGGCAAAGAGATCAAAAATAAAGACGAGATCTTGGCCCTACTAAAAGCCCTCTTTCTGCCCAAAAGACTTAGCATAATCCATTGTCCAGGACATCAAAAGGGACACAGCGCCGAGGCTAGAGGCAACCGGATGGCTGACCAAGCGGCCCGAAAGGCAGCCATCACAGAGACTCCAGACACCTCTACCCTCCTCATAGAAAATTCATCACCCTCTGGCGGCTCAAAAAGAACCGCCGACGGCAGCGAATTCGAGCCCAAGAAGAAGAGGAAAGTCTAACCGGTCATCATCACCATCACCATTGAGTTTTACCCCTACGACGTGCCCGACTACGCCTAATAACTCGAGCATGCATCTAGAGGGCCCTATTCTATAGTGTCACCTAAATGCTAGAGCTCGCTGATCAGCCTCGACTGTGCCTTCTAGTTGCCAGCCATCTGTTGTTTGCCCCTCCCCCGTGCCTTCCTTGACCCTGGAAGGTGCCACTCCCACTGTCCTTTCCTAATAAAATGAGGAAATTGCATCGCATTGTCTGAGTAGGTGTCATTCTATTCTGGGGGGTGGGGTGGGGCAGGACAGCAAGGGGGAGGATTGGGAAGACAATAGCAGGCATGCTGGGGAGGATCTGCGATCGCTCCGGTGCCCGTCAGTGGGCAGAGCGCACATCGCCCACAGTCCCCGAGAAGTTGGGGGGAGGGGTCGGCAATTGAACGGGTGCCTAGAGAAGGTGGCGCGGGGTAAACTGGGAAAGTGATGTCGTGTACTGGCTCCGCCTTTTTCCCGAGGGTGGGGGAGAACCGTATATAAGTGCAGTAGTCGCCGTGAACGTTCTTTTTCGCAACGGGTTTGCCGCCAGAACACAGCTGAAGCTTCGAGGGGCTCGCATCTCTCCTTCACGCGCCCGCCGCCCTACCTGAGGCCGCCATCCACGCCGGTTGAGTCGCGTTCTGCCGCCTCCCGCCTGTGGTGCCTCCTGAACTGCGTCCGCCGTCTAGGTAAGTTTAAAGCTCAGGTCGAGACCGGGCCTTTGTCCGGCGCTCCCTTGGAGCCTACCTAGACTCAGCCGGCTCTCCACGCTTTGCCTGACCCTGCTTGCTCAACTCTACGTCTTTGTTTCGTTTTCTGTTCTGCGCCGTTACAGATCCAAGCTGTGACCGGCGCCTACATGACCGAGTACAAGCCCACGGTGCGCCTCGCCACCCGCGACGACGTCCCCAGGGCCGTACGCACCCTCGCCGCCGCGTTCGCCGACTACCCCGCCACGCGCCACACCGTCGATCCAGACCGCCACATCGAGCGGGTCACCGAGCTGCAAGAACTCTTCCTCACGCGCGTCGGGCTCGACATCGGCAAGGTGTGGGTCGCGGACGACGGCGCCGCGGTGGCGGTCTGGACCACGCCGGAGAGCGTCGAAGCGGGGGCGGTGTTCGCCGAGATCGGCCCGCGCATGGCCGAGTTGAGCGGTTCCCGGCTGGCCGCGCAGCAACAGATGGAAGGTCTCCTGGCGCCGCACCGGCCCAAGGAGCCCGCGTGGTTCCTGGCCACCGTCGGCGTCTCGCCCGACCACCAGGGCAAGGGTCTGGGCAGCGCCGTCGTGCTCCCCGGAGTGGAGGCGGCCGAGCGCGCCGGGGTGCCCGCCTTCCTGGAGACCTCCGCGCCCCGCAACCTCCCCTTCTACGAGCGGCTCGGCTTCACCGTCACCGCCGACGTCGAGGTGCCCGAAGGACCGCGCACCTGGTGCATGACCCGCAAGCCCGGTGCCTGAGCTAGAAGCTCGCTTTCTTGCTGTCCAATTTCTATTAAAGGTTCCTTTGTTCCCTAAGTCCAACTACTAAACTGGGGGATATTATGAAGGGCCTTGAGCATCTGGATTCTGCCTAATAAAAAACATTTATTTTCATTGCAATGATGTATTTAAATTATTTCTGAATATTTTACTAAAAAGGGAATGTGGGAGGTCAGTGCATTTAAAACATAAAGAAATGAAGAGCTAGTTCAAACCTTGGGAAAATACACTATATCTTAAACTCCATGAAAGAAGGTGAGGCTGCAAACAGCTAATGCACATTGGCAACAGCCCCTGATGCCTATGCCTTATTCATCCCTCAGAAAAGGATTCAAGTAGAGGCTTGATTTGGAGGTTAAAGTTTTGCTATGCTGTATTTTAGAATTCCATACCACATTTGTAGAGGTTTTACTTGCTTTAAAAAACCTCCCACACCTCCCCCTGAACCTGAAACATAAAATGAATGCAATTGTTGTTGTTTTACCAATGCTTAATCAGTGAGGCACCTATCTCAGCGATCTGTCTATTTCGTTCATCCATAGTTGCCTGACTCCCCGTCGTGTAGATAACTACGATACGGGAGGGCTTACCATCTGGCCCCAGTGCTGCAATGATACCGCGAGACCCACGCTCACCGGCTCCAGATTTATCAGCAATAAACCAGCCAGCCGGAAGGGCCGAGCGCAGAAGTGGTCCTGCAACTTTATCCGCCTCCATCCAGTCTATTAATTGTTGCCGGGAAGCTAGAGTAAGTAGTTCGCCAGTTAATAGTTTGCGCAACGTTGTTGCCATTGCTACAGGCATCGTGGTGTCACGCTCGTCGTTTGGTATGGCTTCATTCAGCTCCGGTTCCCAACGATCAAGGCGAGTTACATGATCCCCCATGTTGTGCAAAAAAGCGGTTAGCTCCTTCGGTCCTCCGATCGTTGTCAGAAGTAAGTTGGCCGCAGTGTTATCACTCATGGTTATGGCAGCACTGCATAATTCTCTTACTGTCATGCCATCCGTAAGATGCTTTTCTGTGACTGGTGAGTACTCAACCAAGTCATTCTGAGAATAGTGTATGCGGCGACCGAGTTGCTCTTGCCCGGCGTCAATACGGGATAATACCGCGCCACATAGCAGAACTTTAAAAGTGCTCATCATTGGAAAACGTTCTTCGGGGCGAAAACTCTCAAGGATCTTACCGCTGTTGAGATCCAGTTCGATGTAACCCACTCGTGCACCCAACTGATCTTCAGCATCTTTTACTTTCACCAGCGTTTCTGGGTGAGCAAAAACAGGAAGGCAAAATGCCGCAAAAAAGGGAATAAGGGCGACACGGAAATGTTGAATACTCATACTCTTCCTTTTTCAATATTATTGAAGCATTTATCAGGGTTATTGTCTCATGAGCGGATACATATTTGAATGTATTTAGAAAAATAAACAAATAGGGGTTCCGCGAACTTGTTTATTGCAGCTTATAATGGTTACAAATAAAGCAATAGCATCACAAATTTCACAAATAAAGCATTTTTTTCACTGCATTCTAGTTGTGGTTTGTCCAAACTCATCAATGTATCTTATCATGTCTGGCCAGCTAGCCAACAAGCTCGTCATCGCTTTGCAGAAGAGCAGAGAGGATATGCTCATCGTCTAAAGAACTACCCATTTTATTATATATTAGTCACGATATCTATAACAAGAAAATATATATATAATAAGTTATCACGTAAGTAGAACATGAAATAACAATATAATTATCGTATGAGTTAAATCTTAAAAGTCACGTAAAAGATAATCATGCGTCATTTTGACTCACGCGGTCGTTATAGTTCAAAATCAGTGACACTTACCGCATTGACAAGCACGCCTCACGGGAGCTCCAAGCGGCGACTGAGATGTCCTAAATGCACAGCGACGGATTCGCGCTATTTAGAAAGAGAGAGCAATATTTCAAGAATGCATGCGTCAATTTTACGCAGACTATCTTTCTAGGGTTAAAAAAGATTTGCGCTTTACTCGACCTAAACTTTAAACACGTCATAGAATCTTCGTTTGACAAAAACCACATTGTGGCCAAGCTGTGTGACGCGACGCGCGCTAAAGAATGGCAAACCAAGTCGCGCGAGGTACCCAGCTTTTGTTCCCTTTAGTGAGGGTTAATTCCGAGCTTGGCGTAATCATGGTCATAGCTGTTTCCTGTGTGAAATTGTTATCCGCTCACAATTCCACACAACATACGAGCCGGAAGCATAAAGTGTAAAGCCTGGGGTGCCTAATGAGTGAGCTAACTCACATTAATTGCGTTGCGCTCACTGCCCGCTTTCCAGTCGGGAAACCTGTCGTGCCAGCTGCATTAATGAATCGGCCAACGCGCGGGGAGAGGCGGTTTGCGTATTGGGCGCTCTTCCGCTTCCTCGCTCACTGACTCGCTGCGCTCGGTCGTTCGGCTGCGGCGAGCGGTATCAGCTCACTCAAAGGCGGTAATACGGTTATCCACAGAATCAGGGGATAACGCAGGAAAGAACATGTGAGCAAAAGGCCAGCAAAAGGCCAGGAACCGTAAAAAGGCCGCGTTGCTGGCGTTTTTCCATAGGCTCCGCCCCCCTGACGAGCATCACAAAAATCGACGCTCAAGTCAGAGGTGGCGAAACCCGACAGGACTATAAAGATACCAGGCGTTTCCCCCTGGAAGCTCCCTCGTGCGCTCTCCTGTTCCGACCCTGCCGCTTACCGGATACCTGTCCGCCTTTCTCCCTTCGGGAAGCGTGGCGCTTTCTCATAGCTCACGCTGTAGGTATCTCAGTTCGGTGTAGGTCGTTCGCTCCAAGCTGGGCTGTGTGCACGAACCCCCCGTTCAGCCCGACCGCTGCGCCTTATCCGGTAACTATCGTCTTGAGTCCAACCCGGTAAGACACGACTTATCGCCACTGGCAGCAGCCACTGGTAACAGGATTAGCAGAGCGAGGTATGTAGGCGGTGCTACAGAGTTCTTGAAGTGGTGGCCTAACTACGGCTACACTAGAAGGACAGTATTTGGTATCTGCGCTCTGCTGAAGCCAGTTACCTTCGGAAAAAGAGTTGGTAGCTCTTGATCCGGCAAACAAACCACCGCTGGTAGCGGTGGTTTTTTTGTTTGCAAGCAGCAGATTACGCGCAGAAAAAAAGGATCTCAAGAAGATCCTTTGATCTTTTCTACGGGGTCTGACGCTCAGTGGAACGAAAACTCACGTTAAGGGATTTTGGTCATGATTAACCCTAGAAAGATAATCATATTGTGACGTACGTTAAAGATAATCATGCGTAAAATTGACGCATGTGTTTTATCGGTCTGTATATCGAGGTTTATTTATTAATTTGAATAGATATTAAGTTTTATTATATTTACACTTACATACTAATAATAAATTCAACAAACAATTTATTTATGTTTATTTATTTATTAAAAAAAAACAAAAACTCAAAATTTCTTCTATAAAGTAACAAAACTTTTAAACATTCTCTCTTTTACAAAAATAAACTTATTTTGTACTTTAAAAACAGTCATGTTGTATTATAAAATAAGTAATTAGCTTAACCTATACATAATAGAAACAAATTATACTTATTAGTCAGTCAGAAACAACTTTGGCACATATCAATATTATGCTCTCGTTAATTAA

pRG2-epegRNA

U6 promoter Stuffer sgRNA scaffold tevopreQ1 pUC origin Amp resistance

GACGAAGACTCAATTGTCGATTAGTGAACGGATCTCGACGGTATCGATCACGAGACTAGCCTCGAGCGGCCGCCCCCTTCACCGAGGGCCTATTTCCCATGATTCCTTCATATTTGCATATACGATACAAGGCTGTTAGAGAGATAATTGGAATTAATTTGACTGTAAACACAAAGATATTAGTACAAAATACGTGACGTAGAAAGTAATAATTTCTTGGGTAGTTTGCAGTTTTAAAATTATGTTTTAAAATGGACTATCATATGCTTACCGTAACTTGAAAGTATTTCGATTTCTTGGCTTTATATATCTTGTGGAAAGGACGAAACACCGGAGACCACATTTCCCCGAAAAGTGCCACCTGACGTCTAAGAAACCATTATTATCATGACATTAACCTATAAAAATAGGCGTATCACGAGGCCCTTTCGTCTCGCGCGTTTCGGTGATGACGGTGAAAACCTCTGACACATGCAGCTCCCGGAGACGGTCACAGCTTGTCTGTAAGCGGATGCCGGGAGCAGACAAGCCCGTCAGGGCGCGTCAGCGGGTGTTGGCGGGTGTCGGGGCTGGCTTAACTATGCGGCATCAGAGCAGATTGTACTGAGAGTGCACCATATGCGGTGTGAAATACCGCACAGATGCGTAAGGAGAAAATACCGCATCAGGCGCCATTCGCCATTCAGGCTGCGCAACTGTTGGGAAGGGCGATCGGTGCGGGCCTCTTCGCTATTACGCCAGCTGGCGAAAGGGGGATGTGCTGCAAGGCGATTAAGTTGGGTAACGCCAGGGTTTTCCCAGTCACGACGTTGTAAAACGACGGCCAGTGCCAAGCTTGCATGCCTGCAGGTCGACTCTAGAGGATCCCCGGGTACCGAGCTCGAATTCGTAATCATGGTCATAGCTGTTTCCTGTGTGAAATTGTTATCCGCTCACAATTCCACACAACATACGAGCCGGAAGCATAAAGTGTAAAGCCTGGGGTGCCTAATAGATCCGGTCTCCGTTTTAGAGCTAGAAATAGCAAGTTAAAATAAGGCTAGTCCGTTATCAACTTGAAAAAGTGGCACCGAGTCGGTGCGGAGACGTGTCTGTAAGCGGATGCCGGGAGCAGACAAGCCCGTCAGGGCGCGTCAGCGGGTGTTGGCGGGTGTCGGGGCTGGCTTAACTATGCGGCATCAGAGCAGATTGTACTGAGAGTGCACCATATGCGGTGTGAAATACCGCACAGATGCGTAAGGAGAAAATACCGCATCAGGCGCCATTCGCCATTCAGGCTGCGCAACTGTTGGGAAGGGCGATCGGTGCGGGCCTCTTCGCTATTACGCCAGCTGGCGAAAGGGGGATGTGCTGCAAGGCGATTAAGTTGGGTAACGCCAGGGTTTTCCCAGTCACGACGTTGTAAAACGACGGCCAGTGCCAAGCTTGCATGCCTGCAGGTCGACTCTAGAGGATCCCCGGGTACCGAGCTCGAATTCGTAATCATGGTCATAGCTGTTTCCTGTGTGAAATTGTTATCCGCTCACAATTCCACACAACATACGAGCCGGCGTCTCCCGCGGTTCTATCTAGTTACGCGTTAAACCAACTAGAATTTTTTAGATATACGCGTTGACATTGATTATTGACTAGTTGTCTTCCTGCATTAATGAATCGGCCAACGCGCGGGGAGAGGCGGTTTGCGTATTGGGCGCTCTTCCGCTTCCTCGCTCACTGACTCGCTGCGCTCGGTCGTTCGGCTGCGGCGAGCGGTATCAGCTCACTCAAAGGCGGTAATACGGTTATCCACAGAATCAGGGGATAACGCAGGAAAGAACATGTGAGCAAAAGGCCAGCAAAAGGCCAGGAACCGTAAAAAGGCCGCGTTGCTGGCGTTTTTCCATAGGCTCCGCCCCCCTGACGAGCATCACAAAAATCGACGCTCAAGTCAGAGGTGGCGAAACCCGACAGGACTATAAAGATACCAGGCGTTTCCCCCTGGAAGCTCCCTCGTGCGCTCTCCTGTTCCGACCCTGCCGCTTACCGGATACCTGTCCGCCTTTCTCCCTTCGGGAAGCGTGGCGCTTTCTCAATGCTCACGCTGTAGGTATCTCAGTTCGGTGTAGGTCGTTCGCTCCAAGCTGGGCTGTGTGCACGAACCCCCCGTTCAGCCCGACCGCTGCGCCTTATCCGGTAACTATCGTCTTGAGTCCAACCCGGTAAGACACGACTTATCGCCACTGGCAGCAGCCACTGGTAACAGGATTAGCAGAGCGAGGTATGTAGGCGGTGCTACAGAGTTCTTGAAGTGGTGGCCTAACTACGGCTACACTAGAAGGACAGTATTTGGTATCTGCGCTCTGCTGAAGCCAGTTACCTTCGGAAAAAGAGTTGGTAGCTCTTGATCCGGCAAACAAACCACCGCTGGTAGCGGTGGTTTTTTTGTTTGCAAGCAGCAGATTACGCGCAGAAAAAAAGGATCTCAAGAAGATCCTTTGATCTTTTCTACGGGGTCTGACGCTCAGTGGAACGAAAACTCACGTTAAGGGATTTTGGTCATGAGATTATCAAAAAGGATCTTCACCTAGATCCTTTTAAATTAAAAATGAAGTTTTAAATCAATCTAAAGTATATATGAGTAAACTTGGTCTGACAGTTACCAATGCTTAATCAGTGAGGCACCTATCTCAGCGATCTGTCTATTTCGTTCATCCATAGTTGCCTGACTCCCCGTCGTGTAGATAACTACGATACGGGAGGGCTTACCATCTGGCCCCAGTGCTGCAATGATACCGCGAGATCCACGCTCACCGGCTCCAGATTTATCAGCAATAAACCAGCCAGCCGGAAGGGCCGAGCGCAGAAGTGGTCCTGCAACTTTATCCGCCTCCATCCAGTCTATTAATTGTTGCCGGGAAGCTAGAGTAAGTAGTTCGCCAGTTAATAGTTTGCGCAACGTTGTTGCCATTGCTACAGGCATCGTGGTGTCACGCTCGTCGTTTGGTATGGCTTCATTCAGCTCCGGTTCCCAACGATCAAGGCGAGTTACATGATCCCCCATGTTGTGCAAAAAAGCGGTTAGCTCCTTCGGTCCTCCGATCGTTGTCAGAAGTAAGTTGGCCGCAGTGTTATCACTCATGGTTATGGCAGCACTGCATAATTCTCTTACTGTCATGCCATCCGTAAGATGCTTTTCTGTGACTGGTGAGTACTCAACCAAGTCATTCTGAGAATAGTGTATGCGGCGACCGAGTTGCTCTTGCCCGGCGTCAATACGGGATAATACCGCGCCACATAGCAGAACTTTAAAAGTGCTCATCATTGGAAAACGTTCTTCGGGGCGAAAACTCTCAAGGATCTTACCGCTGTTGAGATCCAGTTCGATGTAACCCACTCGTGCACCCAACTGATCTTCAGCATCTTTTACTTTCACCAGCGTTTCTGGGTGAGCAAAAACAGGAAGGCAAAATGCCGCAAAAAAGGGAATAAGGGCGACACGGAAATGTTGAATACTCATACTCTTCCTTTTTCAATATTATTGAAGCATTTATCAGGGTTATTGTCTCATGAGCGGATACATATTTGAATGTATTTAGAAAAATAAACAAATAGGGGTTCCGCGCACATTTCCCCGAAAAGTGCCACCTGACGTC

pAllin1-PE4

pegRNA U6 Promoter CMV Promoter PE4 EF1alpha Promoter Puromycin resistance ITR

CGATGTACGGGCCAGATATACGCGCGTATATCTGGCCCGTACATCGCGAATCTAGAAAAAANNNNNNNNNNNNNNNNNNNNNNNNNNNNNGCACCGACTCGGTGCCACTTTTTCAAGTTGATAACGGACTAGCCTTATTTTAACTTGCTATTTCTAGCTCTAAAACNNNNNNNNNNNNNNNNNNNCGGTGTTTCGTCCTTTCCACAAGATATATAAAGCCAAGAAATCGAAATACTTTCAAGTTACGGTAAGCATATGATAGTCCATTTTAAAACATAATTTTAAAACTGCAAACTACCCAAGAAATTATTACTTTCTACGTCACGTATTTTGTACTAATATCTTTGTGTTTACAGTCAAATTAATTCCAATTATCTCTCTAACAGCCTTGTATCGTATATGCAAATATGAAGGAATCATGGGAAATAGGCCCTCCGCGTTGACATTGATTATTGACTAGTTATTAATAGTAATCAATTACGGGGTCATTAGTTCATAGCCCATATATGGAGTTCCGCGTTACATAACTTACGGTAAATGGCCCGCCTGGCTGACCGCCCAACGACCCCCGCCCATTGACGTCAATAATGACGTATGTTCCCATAGTAACGCCAATAGGGACTTTCCATTGACGTCAATGGGTGGAGTATTTACGGTAAACTGCCCACTTGGCAGTACATCAAGTGTATCATATGCCAAGTACGCCCCCTATTGACGTCAATGACGGTAAATGGCCCGCCTGGCATTATGCCCAGTACATGACCTTATGGGACTTTCCTACTTGGCAGTACATCTACGTATTAGTCATCGCTATTACCATGGTGATGCGGTTTTGGCAGTACATCAATGGGCGTGGATAGCGGTTTGACTCACGGGGATTTCCAAGTCTCCACCCCATTGACGTCAATGGGAGTTTGTTTTGGCACCAAAATCAACGGGACTTTCCAAAATGTCGTAACAACTCCGCCCCATTGACGCAAATGGGCGGTAGGCGTGTACGGTGGGAGGTCTATATAAGCAGAGCTGGTTTAGTGAACCGTCAGATCCGCTAGAGATCCGCGGCCGCTAATACGACTCACTATAGGGAGAGCCGCCACCATGAAACGGACAGCCGACGGAAGCGAGTTCGAGTCACCAAAGAAGAAGCGGAAAGTCGACAAGAAGTACAGCATCGGCCTGGACATCGGCACCAACTCTGTGGGCTGGGCCGTGATCACCGACGAGTACAAGGTGCCCAGCAAGAAATTCAAGGTGCTGGGCAACACCGACCGGCACAGCATCAAGAAGAACCTGATCGGAGCCCTGCTGTTCGACAGCGGCGAAACAGCCGAGGCCACCCGGCTGAAGAGAACCGCCAGAAGAAGATACACCAGACGGAAGAACCGGATCTGCTATCTGCAAGAGATCTTCAGCAACGAGATGGCCAAGGTGGACGACAGCTTCTTCCACAGACTGGAAGAGTCCTTCCTGGTGGAAGAGGATAAGAAGCACGAGCGGCACCCCATCTTCGGCAACATCGTGGACGAGGTGGCCTACCACGAGAAGTACCCCACCATCTACCACCTGAGAAAGAAACTGGTGGACAGCACCGACAAGGCCGACCTGCGGCTGATCTATCTGGCCCTGGCCCACATGATCAAGTTCCGGGGCCACTTCCTGATCGAGGGCGACCTGAACCCCGACAACAGCGACGTGGACAAGCTGTTCATCCAGCTGGTGCAGACCTACAACCAGCTGTTCGAGGAAAACCCCATCAACGCCAGCGGCGTGGACGCCAAGGCCATCCTGTCTGCCAGACTGAGCAAGAGCAGACGGCTGGAAAATCTGATCGCCCAGCTGCCCGGCGAGAAGAAGAATGGCCTGTTCGGAAACCTGATTGCCCTGAGCCTGGGCCTGACCCCCAACTTCAAGAGCAACTTCGACCTGGCCGAGGATGCCAAACTGCAGCTGAGCAAGGACACCTACGACGACGACCTGGACAACCTGCTGGCCCAGATCGGCGACCAGTACGCCGACCTGTTTCTGGCCGCCAAGAACCTGTCCGACGCCATCCTGCTGAGCGACATCCTGAGAGTGAACACCGAGATCACCAAGGCCCCCCTGAGCGCCTCTATGATCAAGAGATACGACGAGCACCACCAGGACCTGACCCTGCTGAAAGCTCTCGTGCGGCAGCAGCTGCCTGAGAAGTACAAAGAGATTTTCTTCGACCAGAGCAAGAACGGCTACGCCGGCTACATTGACGGCGGAGCCAGCCAGGAAGAGTTCTACAAGTTCATCAAGCCCATCCTGGAAAAGATGGACGGCACCGAGGAACTGCTCGTGAAGCTGAACAGAGAGGACCTGCTGCGGAAGCAGCGGACCTTCGACAACGGCAGCATCCCCCACCAGATCCACCTGGGAGAGCTGCACGCCATTCTGCGGCGGCAGGAAGATTTTTACCCATTCCTGAAGGACAACCGGGAAAAGATCGAGAAGATCCTGACCTTCCGCATCCCCTACTACGTGGGCCCTCTGGCCAGGGGAAACAGCAGATTCGCCTGGATGACCAGAAAGAGCGAGGAAACCATCACCCCCTGGAACTTCGAGGAAGTGGTGGACAAGGGCGCTTCCGCCCAGAGCTTCATCGAGCGGATGACCAACTTCGATAAGAACCTGCCCAACGAGAAGGTGCTGCCCAAGCACAGCCTGCTGTACGAGTACTTCACCGTGTATAACGAGCTGACCAAAGTGAAATACGTGACCGAGGGAATGAGAAAGCCCGCCTTCCTGAGCGGCGAGCAGAAAAAGGCCATCGTGGACCTGCTGTTCAAGACCAACCGGAAAGTGACCGTGAAGCAGCTGAAAGAGGACTACTTCAAGAAAATCGAGTGCTTCGACTCCGTGGAAATCTCCGGCGTGGAAGATCGGTTCAACGCCTCCCTGGGCACATACCACGATCTGCTGAAAATTATCAAGGACAAGGACTTCCTGGACAATGAGGAAAACGAGGACATTCTGGAAGATATCGTGCTGACCCTGACACTGTTTGAGGACAGAGAGATGATCGAGGAACGGCTGAAAACCTATGCCCACCTGTTCGACGACAAAGTGATGAAGCAGCTGAAGCGGCGGAGATACACCGGCTGGGGCAGGCTGAGCCGGAAGCTGATCAACGGCATCCGGGACAAGCAGTCCGGCAAGACAATCCTGGATTTCCTGAAGTCCGACGGCTTCGCCAACAGAAACTTCATGCAGCTGATCCACGACGACAGCCTGACCTTTAAAGAGGACATCCAGAAAGCCCAGGTGTCCGGCCAGGGCGATAGCCTGCACGAGCACATTGCCAATCTGGCCGGCAGCCCCGCCATTAAGAAGGGCATCCTGCAGACAGTGAAGGTGGTGGACGAGCTCGTGAAAGTGATGGGCCGGCACAAGCCCGAGAACATCGTGATCGAAATGGCCAGAGAGAACCAGACCACCCAGAAGGGACAGAAGAACAGCCGCGAGAGAATGAAGCGGATCGAAGAGGGCATCAAAGAGCTGGGCAGCCAGATCCTGAAAGAACACCCCGTGGAAAACACCCAGCTGCAGAACGAGAAGCTGTACCTGTACTACCTGCAGAATGGGCGGGATATGTACGTGGACCAGGAACTGGACATCAACCGGCTGTCCGACTACGATGTGGACGCTATCGTGCCTCAGAGCTTTCTGAAGGACGACTCCATCGACAACAAGGTGCTGACCAGAAGCGACAAGAACCGGGGCAAGAGCGACAACGTGCCCTCCGAAGAGGTCGTGAAGAAGATGAAGAACTACTGGCGGCAGCTGCTGAACGCCAAGCTGATTACCCAGAGAAAGTTCGACAATCTGACCAAGGCCGAGAGAGGCGGCCTGAGCGAACTGGATAAGGCCGGCTTCATCAAGAGACAGCTGGTGGAAACCCGGCAGATCACAAAGCACGTGGCACAGATCCTGGACTCCCGGATGAACACTAAGTACGACGAGAATGACAAGCTGATCCGGGAAGTGAAAGTGATCACCCTGAAGTCCAAGCTGGTGTCCGATTTCCGGAAGGATTTCCAGTTTTACAAAGTGCGCGAGATCAACAACTACCACCACGCCCACGACGCCTACCTGAACGCCGTCGTGGGAACCGCCCTGATCAAAAAGTACCCTAAGCTGGAAAGCGAGTTCGTGTACGGCGACTACAAGGTGTACGACGTGCGGAAGATGATCGCCAAGAGCGAGCAGGAAATCGGCAAGGCTACCGCCAAGTACTTCTTCTACAGCAACATCATGAACTTTTTCAAGACCGAGATTACCCTGGCCAACGGCGAGATCCGGAAGCGGCCTCTGATCGAGACAAACGGCGAAACCGGGGAGATCGTGTGGGATAAGGGCCGGGATTTTGCCACCGTGCGGAAAGTGCTGAGCATGCCCCAAGTGAATATCGTGAAAAAGACCGAGGTGCAGACAGGCGGCTTCAGCAAAGAGTCTATCCTGCCCAAGAGGAACAGCGATAAGCTGATCGCCAGAAAGAAGGACTGGGACCCTAAGAAGTACGGCGGCTTCGACAGCCCCACCGTGGCCTATTCTGTGCTGGTGGTGGCCAAAGTGGAAAAGGGCAAGTCCAAGAAACTGAAGAGTGTGAAAGAGCTGCTGGGGATCACCATCATGGAAAGAAGCAGCTTCGAGAAGAATCCCATCGACTTTCTGGAAGCCAAGGGCTACAAAGAAGTGAAAAAGGACCTGATCATCAAGCTGCCTAAGTACTCCCTGTTCGAGCTGGAAAACGGCCGGAAGAGAATGCTGGCCTCTGCCGGCGAACTGCAGAAGGGAAACGAACTGGCCCTGCCCTCCAAATATGTGAACTTCCTGTACCTGGCCAGCCACTATGAGAAGCTGAAGGGCTCCCCCGAGGATAATGAGCAGAAACAGCTGTTTGTGGAACAGCACAAGCACTACCTGGACGAGATCATCGAGCAGATCAGCGAGTTCTCCAAGAGAGTGATCCTGGCCGACGCTAATCTGGACAAAGTGCTGTCCGCCTACAACAAGCACCGGGATAAGCCCATCAGAGAGCAGGCCGAGAATATCATCCACCTGTTTACCCTGACCAATCTGGGAGCCCCTGCCGCCTTCAAGTACTTTGACACCACCATCGACCGGAAGAGGTACACCAGCACCAAAGAGGTGCTGGACGCCACCCTGATCCACCAGAGCATCACCGGCCTGTACGAGACACGGATCGACCTGTCTCAGCTGGGAGGTGACTCTGGAGGATCTAGCGGAGGATCCTCTGGCAGCGAGACACCAGGAACAAGCGAGTCAGCAACACCAGAGAGCAGTGGCGGCAGCAGCGGCGGCAGCAGCACCCTAAATATAGAAGATGAGTATCGGCTACATGAGACCTCAAAAGAGCCAGATGTTTCTCTAGGGTCCACATGGCTGTCTGATTTTCCTCAGGCCTGGGCGGAAACCGGGGGCATGGGACTGGCAGTTCGCCAAGCTCCTCTGATCATACCTCTGAAAGCAACCTCTACCCCCGTGTCCATAAAACAATACCCCATGTCACAAGAAGCCAGACTGGGGATCAAGCCCCACATACAGAGACTGTTGGACCAGGGAATACTGGTACCCTGCCAGTCCCCCTGGAACACGCCCCTGCTACCCGTTAAGAAACCAGGGACTAATGATTATAGGCCTGTCCAGGATCTGAGAGAAGTCAACAAGCGGGTGGAAGACATCCACCCCACCGTGCCCAACCCTTACAACCTCTTGAGCGGGCTCCCACCGTCCCACCAGTGGTACACTGTGCTTGATTTAAAGGATGCCTTTTTCTGCCTGAGACTCCACCCCACCAGTCAGCCTCTCTTCGCCTTTGAGTGGAGAGATCCAGAGATGGGAATCTCAGGACAATTGACCTGGACCAGACTCCCACAGGGTTTCAAAAACAGTCCCACCCTGTTTAATGAGGCACTGCACAGAGACCTAGCAGACTTCCGGATCCAGCACCCAGACTTGATCCTGCTACAGTACGTGGATGACTTACTGCTGGCCGCCACTTCTGAGCTAGACTGCCAACAAGGTACTCGGGCCCTGTTACAAACCCTAGGGAACCTCGGGTATCGGGCCTCGGCCAAGAAAGCCCAAATTTGCCAGAAACAGGTCAAGTATCTGGGGTATCTTCTAAAAGAGGGTCAGAGATGGCTGACTGAGGCCAGAAAAGAGACTGTGATGGGGCAGCCTACTCCGAAGACCCCTCGACAACTAAGGGAGTTCCTAGGGAAGGCAGGCTTCTGTCGCCTCTTCATCCCTGGGTTTGCAGAAATGGCAGCCCCCCTGTACCCTCTCACCAAACCGGGGACTCTGTTTAATTGGGGCCCAGACCAACAAAAGGCCTATCAAGAAATCAAGCAAGCTCTTCTAACTGCCCCAGCCCTGGGGTTGCCAGATTTGACTAAGCCCTTTGAACTCTTTGTCGACGAGAAGCAGGGCTACGCCAAAGGTGTCCTAACGCAAAAACTGGGACCTTGGCGTCGGCCGGTGGCCTACCTGTCCAAAAAGCTAGACCCAGTAGCAGCTGGGTGGCCCCCTTGCCTACGGATGGTAGCAGCCATTGCCGTACTGACAAAGGATGCAGGCAAGCTAACCATGGGACAGCCACTAGTCATTCTGGCCCCCCATGCAGTAGAGGCACTAGTCAAACAACCCCCCGACCGCTGGCTTTCCAACGCCCGGATGACTCACTATCAGGCCTTGCTTTTGGACACGGACCGGGTCCAGTTCGGACCGGTGGTAGCCCTGAACCCGGCTACGCTGCTCCCACTGCCTGAGGAAGGGCTGCAACACAACTGCCTTGATATCCTGGCCGAAGCCCACGGAACCCGACCCGACCTAACGGACCAGCCGCTCCCAGACGCCGACCACACCTGGTACACGGATGGAAGCAGTCTCTTACAAGAGGGACAGCGTAAGGCGGGAGCTGCGGTGACCACCGAGACCGAGGTAATCTGGGCTAAAGCCCTGCCAGCCGGGACATCCGCTCAGCGGGCTGAACTGATAGCACTCACCCAGGCCCTAAAGATGGCAGAAGGTAAGAAGCTAAATGTTTATACTGATAGCCGTTATGCTTTTGCTACTGCCCATATCCATGGAGAAATATACAGAAGGCGTGGGTGGCTCACATCAGAAGGCAAAGAGATCAAAAATAAAGACGAGATCTTGGCCCTACTAAAAGCCCTCTTTCTGCCCAAAAGACTTAGCATAATCCATTGTCCAGGACATCAAAAGGGACACAGCGCCGAGGCTAGAGGCAACCGGATGGCTGACCAAGCGGCCCGAAAGGCAGCCATCACAGAGACTCCAGACACCTCTACCCTCCTCATAGAAAATTCATCACCCTCTGGCGGCTCAAAAAGAACCGCCGACGGCAGCGAATTCGAGCCCAAGAAGAAGAGGAAAGTCGGAAGCGGAGCTACTAACTTCAGCCTGCTGAAGCAGGCTGGAGACGTGGAGGAGAACCCTGGACCTAGCTTCGTTGCTGGAGTCATCCGGAGACTGGACGAGACAGTGGTGAACAGAATTGCCGCCGGCGAGGTGATCCAGAGACCTGCCAATGCAATAAAGGAGATGATCGAGAACTGTCTGGACGCCAAGTCCACAAGCATTCAGGTGATCGTGAAGGAGGGCGGACTGAAGCTGATCCAGATCCAAGACAACGGCACAGGCATCAGAAAGGAAGATCTGGACATCGTGTGTGAACGGTTCACCACATCTAAGCTGCAGTCTTTTGAGGATCTGGCCTCTATCAGTACCTACGGCTTCAGAGGCGAGGCCCTGGCCAGCATCAGCCACGTGGCCCATGTGACCATCACCACCAAAACCGCCGACGGCAAATGCGCTTATCGCGCTAGCTACAGCGACGGCAAGCTGAAAGCCCCGCCAAAGCCTTGCGCCGGCAACCAGGGTACACAGATAACAGTGGAGGATCTGTTCTACAACATCGCCACCCGGAGAAAGGCCCTGAAAAATCCCAGCGAGGAGTACGGCAAGATCCTGGAAGTCGTCGGCAGATACTCCGTGCACAACGCCGGAATCAGCTTTAGCGTAAAGAAGCAGGGAGAAACCGTGGCCGATGTGCGCACCCTGCCAAATGCCAGCACCGTGGATAACATCAGAAGCATTTTCGGAAATGCCGTGTCCAGAGAACTGATCGAGATCGGCTGCGAAGATAAGACCCTGGCTTTTAAGATGAACGGCTACATCAGCAACGCCAATTACTCTGTGAAGAAGTGCATCTTTCTTCTGTTCATCAACCACAGACTGGTGGAAAGCACCAGCCTGCGGAAAGCCATCGAGACAGTGTACGCCGCCTACCTGCCTAAGAACACCCACCCCTTCCTGTACCTGAGCCTCGAGATCAGCCCTCAGAACGTGGACGTCAATGTGCATCCTACAAAGCACGAGGTGCACTTCCTGCACGAGGAAAGCATCCTGGAAAGAGTGCAGCAGCACATTGAGAGCAAGCTGCTGGGCTCTAACAGCAGCAGAATGTACTTCACACAGACCCTGCTGCCTGGCCTGGCCGGCCCCTCAGGCGAAATGGTTAAGTCCACAACCTCTCTGACCTCATCCAGCACCAGCGGTTCTTCCGATAAGGTGTACGCCCACCAGATGGTGCGGACCGACTCTCGGGAGCAGAAGCTGGACGCCTTTCTGCAACCTCTGAGCAAACCTCTGAGCTCTCAGCCTCAGGCCATCGTGACCGAGGACAAGACAGATATCTCCTCCGGCCGTGCCAGACAGCAGGACGAAGAAATGCTCGAGCTGCCAGCTCCTGCCGAGGTGGCCGCCAAGAACCAGAGCCTGGAGGGAGATACCACAAAGGGCACCAGCGAAATGAGCGAGAAGCGGGGCCCTACCTCCAGCAACCCCAGAAAACGGCACCGGGAGGACAGCGACGTGGAAATGGTGGAGGACGACAGCAGAAAGGAAATGACAGCCGCTTGTACCCCTAGAAGAAGAATCATCAACCTGACCTCCGTGCTGAGCCTGCAGGAGGAGATCAACGAGCAGGGCCACGAGGTGCTGAGAGAGATGCTGCACAATCACAGCTTCGTGGGCTGCGTGAACCCTCAATGGGCCCTGGCTCAGCATCAAACAAAGCTGTACCTGCTGAACACCACCAAGCTGAGCGAAGAGCTGTTCTACCAGATCCTCATCTACGACTTCGCCAACTTCGGCGTGCTACGCCTGAGCGAGCCCGCCCCTCTGTTTGACCTGGCCATGCTGGCTCTGGATAGCCCAGAAAGCGGCTGGACAGAAGAGGACGGACCTAAAGAGGGGCTGGCTGAATACATCGTGGAGTTCCTGAAGAAAAAGGCCGAGATGCTGGCCGACTACTTTTCTCTGGAAATCGACGAGGAAGGCAACCTGATCGGCCTGCCTCTGCTGATCGATAACTACGTGCCTCCCCTGGAAGGCCTGCCCATCTTCATCCTGAGACTGGCTACAGAGGTGAACTGGGACGAGGAAAAGGAATGCTTCGAGTCTCTGAGCAAGGAGTGCGCCATGTTCTATAGCATCAGAAAACAGTACATCTCTGAAGAGAGCACTCTGTCTGGCCAGCAGAGTGAAGTGCCCGGAAGCATCCCCAACAGCTGGAAGTGGACCGTGGAACACATCGTGTACAAGGCCCTGCGGAGCCACATTCTCCCTCCTAAGCACTTCACCGAGGACGGCAACATCCTGCAGCTGGCCAACCTGCCCGACCTTTATAAGGTTTTCTAACTCGAGCATGCATCTAGAGGGCCCTATTCTATAGTGTCACCTAAATGCTAGAGCTCGCTGATCAGCCTCGACTGTGCCTTCTAGTTGCCAGCCATCTGTTGTTTGCCCCTCCCCCGTGCCTTCCTTGACCCTGGAAGGTGCCACTCCCACTGTCCTTTCCTAATAAAATGAGGAAATTGCATCGCATTGTCTGAGTAGGTGTCATTCTATTCTGGGGGGTGGGGTGGGGCAGGACAGCAAGGGGGAGGATTGGGAAGACAATAGCAGGCATGCTGGGGAGGATCTGCGATCGCTCCGGTGCCCGTCAGTGGGCAGAGCGCACATCGCCCACAGTCCCCGAGAAGTTGGGGGGAGGGGTCGGCAATTGAACGGGTGCCTAGAGAAGGTGGCGCGGGGTAAACTGGGAAAGTGATGTCGTGTACTGGCTCCGCCTTTTTCCCGAGGGTGGGGGAGAACCGTATATAAGTGCAGTAGTCGCCGTGAACGTTCTTTTTCGCAACGGGTTTGCCGCCAGAACACAGCTGAAGCTTCGAGGGGCTCGCATCTCTCCTTCACGCGCCCGCCGCCCTACCTGAGGCCGCCATCCACGCCGGTTGAGTCGCGTTCTGCCGCCTCCCGCCTGTGGTGCCTCCTGAACTGCGTCCGCCGTCTAGGTAAGTTTAAAGCTCAGGTCGAGACCGGGCCTTTGTCCGGCGCTCCCTTGGAGCCTACCTAGACTCAGCCGGCTCTCCACGCTTTGCCTGACCCTGCTTGCTCAACTCTACGTCTTTGTTTCGTTTTCTGTTCTGCGCCGTTACAGATCCAAGCTGTGACCGGCGCCTACATGACCGAGTACAAGCCCACGGTGCGCCTCGCCACCCGCGACGACGTCCCCAGGGCCGTACGCACCCTCGCCGCCGCGTTCGCCGACTACCCCGCCACGCGCCACACCGTCGATCCAGACCGCCACATCGAGCGGGTCACCGAGCTGCAAGAACTCTTCCTCACGCGCGTCGGGCTCGACATCGGCAAGGTGTGGGTCGCGGACGACGGCGCCGCGGTGGCGGTCTGGACCACGCCGGAGAGCGTCGAAGCGGGGGCGGTGTTCGCCGAGATCGGCCCGCGCATGGCCGAGTTGAGCGGTTCCCGGCTGGCCGCGCAGCAACAGATGGAAGGTCTCCTGGCGCCGCACCGGCCCAAGGAGCCCGCGTGGTTCCTGGCCACCGTCGGCGTCTCGCCCGACCACCAGGGCAAGGGTCTGGGCAGCGCCGTCGTGCTCCCCGGAGTGGAGGCGGCCGAGCGCGCCGGGGTGCCCGCCTTCCTGGAGACCTCCGCGCCCCGCAACCTCCCCTTCTACGAGCGGCTCGGCTTCACCGTCACCGCCGACGTCGAGGTGCCCGAAGGACCGCGCACCTGGTGCATGACCCGCAAGCCCGGTGCCTGAGCTAGAAGCTCGCTTTCTTGCTGTCCAATTTCTATTAAAGGTTCCTTTGTTCCCTAAGTCCAACTACTAAACTGGGGGATATTATGAAGGGCCTTGAGCATCTGGATTCTGCCTAATAAAAAACATTTATTTTCATTGCAATGATGTATTTAAATTATTTCTGAATATTTTACTAAAAAGGGAATGTGGGAGGTCAGTGCATTTAAAACATAAAGAAATGAAGAGCTAGTTCAAACCTTGGGAAAATACACTATATCTTAAACTCCATGAAAGAAGGTGAGGCTGCAAACAGCTAATGCACATTGGCAACAGCCCCTGATGCCTATGCCTTATTCATCCCTCAGAAAAGGATTCAAGTAGAGGCTTGATTTGGAGGTTAAAGTTTTGCTATGCTGTATTTTAGAATTCCATACCACATTTGTAGAGGTTTTACTTGCTTTAAAAAACCTCCCACACCTCCCCCTGAACCTGAAACATAAAATGAATGCAATTGTTGTTGTTTTACCAATGCTTAATCAGTGAGGCACCTATCTCAGCGATCTGTCTATTTCGTTCATCCATAGTTGCCTGACTCCCCGTCGTGTAGATAACTACGATACGGGAGGGCTTACCATCTGGCCCCAGTGCTGCAATGATACCGCGAGACCCACGCTCACCGGCTCCAGATTTATCAGCAATAAACCAGCCAGCCGGAAGGGCCGAGCGCAGAAGTGGTCCTGCAACTTTATCCGCCTCCATCCAGTCTATTAATTGTTGCCGGGAAGCTAGAGTAAGTAGTTCGCCAGTTAATAGTTTGCGCAACGTTGTTGCCATTGCTACAGGCATCGTGGTGTCACGCTCGTCGTTTGGTATGGCTTCATTCAGCTCCGGTTCCCAACGATCAAGGCGAGTTACATGATCCCCCATGTTGTGCAAAAAAGCGGTTAGCTCCTTCGGTCCTCCGATCGTTGTCAGAAGTAAGTTGGCCGCAGTGTTATCACTCATGGTTATGGCAGCACTGCATAATTCTCTTACTGTCATGCCATCCGTAAGATGCTTTTCTGTGACTGGTGAGTACTCAACCAAGTCATTCTGAGAATAGTGTATGCGGCGACCGAGTTGCTCTTGCCCGGCGTCAATACGGGATAATACCGCGCCACATAGCAGAACTTTAAAAGTGCTCATCATTGGAAAACGTTCTTCGGGGCGAAAACTCTCAAGGATCTTACCGCTGTTGAGATCCAGTTCGATGTAACCCACTCGTGCACCCAACTGATCTTCAGCATCTTTTACTTTCACCAGCGTTTCTGGGTGAGCAAAAACAGGAAGGCAAAATGCCGCAAAAAAGGGAATAAGGGCGACACGGAAATGTTGAATACTCATACTCTTCCTTTTTCAATATTATTGAAGCATTTATCAGGGTTATTGTCTCATGAGCGGATACATATTTGAATGTATTTAGAAAAATAAACAAATAGGGGTTCCGCGAACTTGTTTATTGCAGCTTATAATGGTTACAAATAAAGCAATAGCATCACAAATTTCACAAATAAAGCATTTTTTTCACTGCATTCTAGTTGTGGTTTGTCCAAACTCATCAATGTATCTTATCATGTCTGGcagctagccaacaagctcgtcatcgctttgcagaagagcagagaggatatgctcatcgtctaaagaactacccattCGATATCTATAACAAGAAAATATATATATAATAAGTTATCACGTAAGTAGAACATGAAATAACAATATAATTATCGTATGAGTTAAATCTTAAAAGTCACGTAAAAGATAATCATGCGTCATTTTGACTCACGCGGTCGTTATAGTTCAAAATCAGTGACACTTACCGCATTGACAAGCACGCCTCACGGGAGCTCCAAGCGGCGACTGAGATGTCCTAAATGCACAGCGACGGATTCGCGCTATTTAGAAAGAGAGAGCAATATTTCAAGAATGCATGCGTCAATTTTACGCAGACTATCTTTCTAGGGTTAAAAAAGATTTGCGCTTTACTCGACCTAAACTTTAAACACGTCATAGAATCTTCGTTTGACAAAAACCACATTGTGGCCAAGCTGTGTGACGCGACGCGCGCTAAAGAATGGCAAACCAAGTCGCGCGAGGTACCCAGCTTTTGTTCCCTTTAGTGAGGGTTAATTCCGAGCTTGGCGTAATCATGGTCATAGCTGTTTCCTGTGTGAAATTGTTATCCGCTCACAATTCCACACAACATACGAGCCGGAAGCATAAAGTGTAAAGCCTGGGGTGCCTAATGAGTGAGCTAACTCACATTAATTGCGTTGCGCTCACTGCCCGCTTTCCAGTCGGGAAACCTGTCGTGCCAGCTGCATTAATGAATCGGCCAACGCGCGGGGAGAGGCGGTTTGCGTATTGGGCGCTCTTCCGCTTCCTCGCTCACTGACTCGCTGCGCTCGGTCGTTCGGCTGCGGCGAGCGGTATCAGCTCACTCAAAGGCGGTAATACGGTTATCCACAGAATCAGGGGATAACGCAGGAAAGAACATGTGAGCAAAAGGCCAGCAAAAGGCCAGGAACCGTAAAAAGGCCGCGTTGCTGGCGTTTTTCCATAGGCTCCGCCCCCCTGACGAGCATCACAAAAATCGACGCTCAAGTCAGAGGTGGCGAAACCCGACAGGACTATAAAGATACCAGGCGTTTCCCCCTGGAAGCTCCCTCGTGCGCTCTCCTGTTCCGACCCTGCCGCTTACCGGATACCTGTCCGCCTTTCTCCCTTCGGGAAGCGTGGCGCTTTCTCATAGCTCACGCTGTAGGTATCTCAGTTCGGTGTAGGTCGTTCGCTCCAAGCTGGGCTGTGTGCACGAACCCCCCGTTCAGCCCGACCGCTGCGCCTTATCCGGTAACTATCGTCTTGAGTCCAACCCGGTAAGACACGACTTATCGCCACTGGCAGCAGCCACTGGTAACAGGATTAGCAGAGCGAGGTATGTAGGCGGTGCTACAGAGTTCTTGAAGTGGTGGCCTAACTACGGCTACACTAGAAGGACAGTATTTGGTATCTGCGCTCTGCTGAAGCCAGTTACCTTCGGAAAAAGAGTTGGTAGCTCTTGATCCGGCAAACAAACCACCGCTGGTAGCGGTGGTTTTTTTGTTTGCAAGCAGCAGATTACGCGCAGAAAAAAAGGATCTCAAGAAGATCCTTTGATCTTTTCTACGGGGTCTGACGCTCAGTGGAACGAAAACTCACGTTAAGGGATTTTGGTCATGATTAACCCTAGAAAGATAATCATATTGTGACGTACGTTAAAGATAATCATGCGTAAAATTGACGCATGTGTTTTATCGGTCTGTATATCGAGGTTTATTTATTAATTTGAATAGATATTAAGTTTTATTATATTTACACTTACATACTAATAATAAATTCAACAAACAATTTATTTATGTTTATTTATTTATTAAAAAAAAACAAAAACTCAAAATTTCTTCTATAAAGTAACAAAacttttaaacattctctcttttacaaaaataaacttattttgtactttaaaaacagtcatgttgtattataaaataagtaattagcttaacctatacataatagaaacaaattatacttattagtcagtcagaaacaaCTTTGGCACATATCAATATTATGCTCTCGTTAATTAA

pAllin1-PE2-nuclease

pegRNA U6 Promoter CMV Promoter PE2-nuclease EF1alpha Promoter Puromycin resistance ITR

CGATGTACGGGCCAGATATACGCGCGTATATCTGGCCCGTACATCGCGAATCTAGAAAAAANNNNNNNNNNNNNNNNNNNNNNNNNNNNNGCACCGACTCGGTGCCACTTTTTCAAGTTGATAACGGACTAGCCTTATTTTAACTTGCTATTTCTAGCTCTAAAACNNNNNNNNNNNNNNNNNNNCGGTGTTTCGTCCTTTCCACAAGATATATAAAGCCAAGAAATCGAAATACTTTCAAGTTACGGTAAGCATATGATAGTCCATTTTAAAACATAATTTTAAAACTGCAAACTACCCAAGAAATTATTACTTTCTACGTCACGTATTTTGTACTAATATCTTTGTGTTTACAGTCAAATTAATTCCAATTATCTCTCTAACAGCCTTGTATCGTATATGCAAATATGAAGGAATCATGGGAAATAGGCCCTCCGCGTTGACATTGATTATTGACTAGTTATTAATAGTAATCAATTACGGGGTCATTAGTTCATAGCCCATATATGGAGTTCCGCGTTACATAACTTACGGTAAATGGCCCGCCTGGCTGACCGCCCAACGACCCCCGCCCATTGACGTCAATAATGACGTATGTTCCCATAGTAACGCCAATAGGGACTTTCCATTGACGTCAATGGGTGGAGTATTTACGGTAAACTGCCCACTTGGCAGTACATCAAGTGTATCATATGCCAAGTACGCCCCCTATTGACGTCAATGACGGTAAATGGCCCGCCTGGCATTATGCCCAGTACATGACCTTATGGGACTTTCCTACTTGGCAGTACATCTACGTATTAGTCATCGCTATTACCATGGTGATGCGGTTTTGGCAGTACATCAATGGGCGTGGATAGCGGTTTGACTCACGGGGATTTCCAAGTCTCCACCCCATTGACGTCAATGGGAGTTTGTTTTGGCACCAAAATCAACGGGACTTTCCAAAATGTCGTAACAACTCCGCCCCATTGACGCAAATGGGCGGTAGGCGTGTACGGTGGGAGGTCTATATAAGCAGAGCTCTCTGGCTAACTAGAGAACCCACTGCTTACTGGCTTATCGAAATTTCCCTATCAGTGATAGAGATTGACATCCCTATCAGTGATAGAGATACTGAGCACATCAGCAGGACGCACTGACCAGGGAGACCCAAGCTTGCCACCATGGTGTACCCCTACGACGTGCCCGACTACGCCGAATTGCCTCCAAAAAAGAAGAGAAAGGTAGGGATCCTGGACAAGAAGTACAGCATCGGCCTGGACATCGGTACCAACAGCGTGGGCTGGGCCGTGATCACCGACGAGTACAAGGTGCCCAGCAAGAAGTTCAAGGTGCTGGGCAACACCGACCGCCACAGCATCAAGAAGAACCTGATCGGCGCCCTGCTGTTCGACAGCGGCGAGACCGCCGAGGCCACCCGCCTGAAGCGCACCGCCCGCCGCCGCTACACCCGCCGCAAGAACCGCATCTGCTACCTGCAGGAGATCTTCAGCAACGAGATGGCCAAGGTGGACGACAGCTTCTTCCACCGCCTGGAGGAGAGCTTCCTGGTGGAGGAGGACAAGAAGCACGAGCGCCACCCCATCTTCGGCAACATCGTGGACGAGGTGGCCTACCACGAGAAGTACCCCACCATCTACCACCTGCGCAAGAAGCTGGTGGACAGCACCGACAAGGCCGACCTGCGCCTGATCTACCTGGCCCTGGCCCACATGATCAAGTTCCGCGGCCACTTCCTGATCGAGGGCGACCTGAACCCCGACAACAGCGACGTGGACAAGCTGTTCATCCAGCTGGTGCAGACCTACAACCAGCTGTTCGAGGAGAACCCCATCAACGCCAGCGGCGTGGACGCCAAGGCCATCCTGAGCGCCCGCCTGAGCAAGAGCCGCCGCCTGGAGAACCTGATCGCCCAGCTGCCCGGCGAGAAGAAGAACGGCCTGTTCGGCAACCTGATCGCCCTGAGCCTGGGCCTGACCCCCAACTTCAAGAGCAACTTCGACCTGGCCGAGGACGCCAAGCTGCAGCTGAGCAAGGACACCTACGACGACGACCTGGACAACCTGCTGGCCCAGATCGGCGACCAGTACGCCGACCTGTTCCTGGCCGCCAAGAACCTGAGCGACGCCATCCTGCTGAGCGACATCCTGCGCGTGAACACCGAGATCACCAAGGCCCCCCTGAGCGCCAGCATGATCAAGCGCTACGACGAGCACCACCAGGACCTGACCCTGCTGAAGGCCCTGGTGCGCCAGCAGCTGCCCGAGAAGTACAAGGAGATCTTCTTCGACCAGAGCAAGAACGGCTACGCCGGCTACATCGACGGCGGCGCCAGCCAGGAGGAGTTCTACAAGTTCATCAAGCCCATCCTGGAGAAGATGGACGGCACCGAGGAGCTGCTGGTGAAGCTGAACCGCGAGGACCTGCTGCGCAAGCAGCGCACCTTCGACAACGGCAGCATCCCCCACCAGATCCACCTGGGCGAGCTGCACGCCATCCTGCGCCGCCAGGAGGACTTCTACCCCTTCCTGAAGGACAACCGCGAGAAGATCGAGAAGATCCTGACCTTCCGCATCCCCTACTACGTGGGCCCCCTGGCCCGCGGCAACAGCCGCTTCGCCTGGATGACCCGCAAGAGCGAGGAGACCATCACCCCCTGGAACTTCGAGGAGGTGGTGGACAAGGGCGCCAGCGCCCAGAGCTTCATCGAGCGCATGACCAACTTCGACAAGAACCTGCCCAACGAGAAGGTGCTGCCCAAGCACAGCCTGCTGTACGAGTACTTCACCGTGTACAACGAGCTGACCAAGGTGAAGTACGTGACCGAGGGCATGCGCAAGCCCGCCTTCCTGAGCGGCGAGCAGAAGAAGGCCATCGTGGACCTGCTGTTCAAGACCAACCGCAAGGTGACCGTGAAGCAGCTGAAGGAGGACTACTTCAAGAAGATCGAGTGCTTCGACAGCGTGGAGATCAGCGGCGTGGAGGACCGCTTCAACGCCAGCCTGGGCACCTACCACGACCTGCTGAAGATCATCAAGGACAAGGACTTCCTGGACAACGAGGAGAACGAGGACATCCTGGAGGACATCGTGCTGACCCTGACCCTGTTCGAGGACCGCGAGATGATCGAGGAGCGCCTGAAGACCTACGCCCACCTGTTCGACGACAAGGTGATGAAGCAGCTGAAGCGCCGCCGCTACACCGGCTGGGGCCGCCTGAGCCGCAAGCTTATCAACGGCATCCGCGACAAGCAGAGCGGCAAGACCATCCTGGACTTCCTGAAGAGCGACGGCTTCGCCAACCGCAACTTCATGCAGCTGATCCACGACGACAGCCTGACCTTCAAGGAGGACATCCAGAAGGCCCAGGTGAGCGGCCAGGGCGACAGCCTGCACGAGCACATCGCCAACCTGGCCGGCAGCCCCGCCATCAAGAAGGGCATCCTGCAGACCGTGAAGGTGGTGGACGAGCTGGTGAAGGTGATGGGCCGCCACAAGCCCGAGAACATCGTGATCGAGATGGCCCGCGAGAACCAGACCACCCAGAAGGGCCAGAAGAACAGCCGCGAGCGCATGAAGCGCATCGAGGAGGGCATCAAGGAGCTGGGCAGCCAGATCCTGAAGGAGCACCCCGTGGAGAACACCCAGCTGCAGAACGAGAAGCTGTACCTGTACTACCTGCAGAACGGCCGCGACATGTACGTGGACCAGGAGCTGGACATCAACCGCCTGAGCGACTACGACGTGGACCACATCGTGCCCCAGAGCTTCCTGAAGGACGACAGCATCGACAACAAGGTGCTGACCCGCAGCGACAAGAACCGCGGCAAGAGCGACAACGTGCCCAGCGAGGAGGTGGTGAAGAAGATGAAGAACTACTGGCGCCAGCTGCTGAACGCCAAGCTGATCACCCAGCGCAAGTTCGACAACCTGACCAAGGCCGAGCGCGGCGGCCTGAGCGAGCTGGACAAGGCCGGCTTCATCAAGCGCCAGCTGGTGGAGACCCGCCAGATCACCAAGCACGTGGCACAGATCCTGGACTCCCGGATGAACACTAAGTACGACGAGAATGACAAGCTGATCCGGGAAGTGAAAGTGATCACCCTGAAGTCCAAGCTGGTGTCCGATTTCCGGAAGGATTTCCAGTTTTACAAAGTGCGCGAGATCAACAACTACCACCACGCCCACGACGCCTACCTGAACGCCGTCGTGGGAACCGCCCTGATCAAAAAGTACCCTAAGCTGGAAAGCGAGTTCGTGTACGGCGACTACAAGGTGTACGACGTGCGGAAGATGATCGCCAAGAGCGAGCAGGAAATCGGCAAGGCTACCGCCAAGTACTTCTTCTACAGCAACATCATGAACTTTTTCAAGACCGAGATTACCCTGGCCAACGGCGAGATCCGGAAGCGGCCTCTGATCGAGACAAACGGCGAAACCGGGGAGATCGTGTGGGATAAGGGCCGGGATTTTGCCACCGTGCGGAAAGTGCTGAGCATGCCCCAAGTGAATATCGTGAAAAAGACCGAGGTGCAGACAGGCGGCTTCAGCAAAGAGTCTATCCTGCCCAAGAGGAACAGCGATAAGCTGATCGCCAGAAAGAAGGACTGGGACCCTAAGAAGTACGGCGGCTTCGACAGCCCCACCGTGGCCTATTCTGTGCTGGTGGTGGCCAAAGTGGAAAAGGGCAAGTCCAAGAAACTGAAGAGTGTGAAAGAGCTGCTGGGGATCACCATCATGGAAAGAAGCAGCTTCGAGAAGAATCCCATCGACTTTCTGGAAGCCAAGGGCTACAAAGAAGTGAAAAAGGACCTGATCATCAAGCTGCCTAAGTACTCCCTGTTCGAGCTGGAAAACGGCCGGAAGAGAATGCTGGCCTCTGCCGGCGAACTGCAGAAGGGAAACGAACTGGCCCTGCCCTCCAAATATGTGAACTTCCTGTACCTGGCCAGCCACTATGAGAAGCTGAAGGGCTCCCCCGAGGATAATGAGCAGAAACAGCTGTTTGTGGAACAGCACAAGCACTACCTGGACGAGATCATCGAGCAGATCAGCGAGTTCTCCAAGAGAGTGATCCTGGCCGACGCTAATCTGGACAAAGTGCTGTCCGCCTACAACAAGCACCGGGATAAGCCCATCAGAGAGCAGGCCGAGAATATCATCCACCTGTTTACCCTGACCAATCTGGGAGCCCCTGCCGCCTTCAAGTACTTTGACACCACCATCGACCGGAAGAGGTACACCAGCACCAAAGAGGTGCTGGACGCCACCCTGATCCACCAGAGCATCACCGGCCTGTACGAGACACGGATCGACCTGTCTCAGCTGGGAGGTGACTCTGGAGGATCTAGCGGAGGATCCTCTGGCAGCGAGACACCAGGAACAAGCGAGTCAGCAACACCAGAGAGCAGTGGCGGCAGCAGCGGCGGCAGCAGCACCCTAAATATAGAAGATGAGTATCGGCTACATGAGACCTCAAAAGAGCCAGATGTTTCTCTAGGGTCCACATGGCTGTCTGATTTTCCTCAGGCCTGGGCGGAAACCGGGGGCATGGGACTGGCAGTTCGCCAAGCTCCTCTGATCATACCTCTGAAAGCAACCTCTACCCCCGTGTCCATAAAACAATACCCCATGTCACAAGAAGCCAGACTGGGGATCAAGCCCCACATACAGAGACTGTTGGACCAGGGAATACTGGTACCCTGCCAGTCCCCCTGGAACACGCCCCTGCTACCCGTTAAGAAACCAGGGACTAATGATTATAGGCCTGTCCAGGATCTGAGAGAAGTCAACAAGCGGGTGGAAGACATCCACCCCACCGTGCCCAACCCTTACAACCTCTTGAGCGGGCTCCCACCGTCCCACCAGTGGTACACTGTGCTTGATTTAAAGGATGCCTTTTTCTGCCTGAGACTCCACCCCACCAGTCAGCCTCTCTTCGCCTTTGAGTGGAGAGATCCAGAGATGGGAATCTCAGGACAATTGACCTGGACCAGACTCCCACAGGGTTTCAAAAACAGTCCCACCCTGTTTAATGAGGCACTGCACAGAGACCTAGCAGACTTCCGGATCCAGCACCCAGACTTGATCCTGCTACAGTACGTGGATGACTTACTGCTGGCCGCCACTTCTGAGCTAGACTGCCAACAAGGTACTCGGGCCCTGTTACAAACCCTAGGGAACCTCGGGTATCGGGCCTCGGCCAAGAAAGCCCAAATTTGCCAGAAACAGGTCAAGTATCTGGGGTATCTTCTAAAAGAGGGTCAGAGATGGCTGACTGAGGCCAGAAAAGAGACTGTGATGGGGCAGCCTACTCCGAAGACCCCTCGACAACTAAGGGAGTTCCTAGGGAAGGCAGGCTTCTGTCGCCTCTTCATCCCTGGGTTTGCAGAAATGGCAGCCCCCCTGTACCCTCTCACCAAACCGGGGACTCTGTTTAATTGGGGCCCAGACCAACAAAAGGCCTATCAAGAAATCAAGCAAGCTCTTCTAACTGCCCCAGCCCTGGGGTTGCCAGATTTGACTAAGCCCTTTGAACTCTTTGTCGACGAGAAGCAGGGCTACGCCAAAGGTGTCCTAACGCAAAAACTGGGACCTTGGCGTCGGCCGGTGGCCTACCTGTCCAAAAAGCTAGACCCAGTAGCAGCTGGGTGGCCCCCTTGCCTACGGATGGTAGCAGCCATTGCCGTACTGACAAAGGATGCAGGCAAGCTAACCATGGGACAGCCACTAGTCATTCTGGCCCCCCATGCAGTAGAGGCACTAGTCAAACAACCCCCCGACCGCTGGCTTTCCAACGCCCGGATGACTCACTATCAGGCCTTGCTTTTGGACACGGACCGGGTCCAGTTCGGACCGGTGGTAGCCCTGAACCCGGCTACGCTGCTCCCACTGCCTGAGGAAGGGCTGCAACACAACTGCCTTGATATCCTGGCCGAAGCCCACGGAACCCGACCCGACCTAACGGACCAGCCGCTCCCAGACGCCGACCACACCTGGTACACGGATGGAAGCAGTCTCTTACAAGAGGGACAGCGTAAGGCGGGAGCTGCGGTGACCACCGAGACCGAGGTAATCTGGGCTAAAGCCCTGCCAGCCGGGACATCCGCTCAGCGGGCTGAACTGATAGCACTCACCCAGGCCCTAAAGATGGCAGAAGGTAAGAAGCTAAATGTTTATACTGATAGCCGTTATGCTTTTGCTACTGCCCATATCCATGGAGAAATATACAGAAGGCGTGGGTGGCTCACATCAGAAGGCAAAGAGATCAAAAATAAAGACGAGATCTTGGCCCTACTAAAAGCCCTCTTTCTGCCCAAAAGACTTAGCATAATCCATTGTCCAGGACATCAAAAGGGACACAGCGCCGAGGCTAGAGGCAACCGGATGGCTGACCAAGCGGCCCGAAAGGCAGCCATCACAGAGACTCCAGACACCTCTACCCTCCTCATAGAAAATTCATCACCCTCTGGCGGCTCAAAAAGAACCGCCGACGGCAGCGAATTCGAGCCCAAGAAGAAGAGGAAAGTCTAACCGGTCATCATCACCATCACCATTGAGTTTTACCCCTACGACGTGCCCGACTACGCCTAATAACTCGAGCATGCATCTAGAGGGCCCTATTCTATAGTGTCACCTAAATGCTAGAGCTCGCTGATCAGCCTCGACTGTGCCTTCTAGTTGCCAGCCATCTGTTGTTTGCCCCTCCCCCGTGCCTTCCTTGACCCTGGAAGGTGCCACTCCCACTGTCCTTTCCTAATAAAATGAGGAAATTGCATCGCATTGTCTGAGTAGGTGTCATTCTATTCTGGGGGGTGGGGTGGGGCAGGACAGCAAGGGGGAGGATTGGGAAGACAATAGCAGGCATGCTGGGGAGGATCTGCGATCGCTCCGGTGCCCGTCAGTGGGCAGAGCGCACATCGCCCACAGTCCCCGAGAAGTTGGGGGGAGGGGTCGGCAATTGAACGGGTGCCTAGAGAAGGTGGCGCGGGGTAAACTGGGAAAGTGATGTCGTGTACTGGCTCCGCCTTTTTCCCGAGGGTGGGGGAGAACCGTATATAAGTGCAGTAGTCGCCGTGAACGTTCTTTTTCGCAACGGGTTTGCCGCCAGAACACAGCTGAAGCTTCGAGGGGCTCGCATCTCTCCTTCACGCGCCCGCCGCCCTACCTGAGGCCGCCATCCACGCCGGTTGAGTCGCGTTCTGCCGCCTCCCGCCTGTGGTGCCTCCTGAACTGCGTCCGCCGTCTAGGTAAGTTTAAAGCTCAGGTCGAGACCGGGCCTTTGTCCGGCGCTCCCTTGGAGCCTACCTAGACTCAGCCGGCTCTCCACGCTTTGCCTGACCCTGCTTGCTCAACTCTACGTCTTTGTTTCGTTTTCTGTTCTGCGCCGTTACAGATCCAAGCTGTGACCGGCGCCTACATGACCGAGTACAAGCCCACGGTGCGCCTCGCCACCCGCGACGACGTCCCCAGGGCCGTACGCACCCTCGCCGCCGCGTTCGCCGACTACCCCGCCACGCGCCACACCGTCGATCCAGACCGCCACATCGAGCGGGTCACCGAGCTGCAAGAACTCTTCCTCACGCGCGTCGGGCTCGACATCGGCAAGGTGTGGGTCGCGGACGACGGCGCCGCGGTGGCGGTCTGGACCACGCCGGAGAGCGTCGAAGCGGGGGCGGTGTTCGCCGAGATCGGCCCGCGCATGGCCGAGTTGAGCGGTTCCCGGCTGGCCGCGCAGCAACAGATGGAAGGTCTCCTGGCGCCGCACCGGCCCAAGGAGCCCGCGTGGTTCCTGGCCACCGTCGGCGTCTCGCCCGACCACCAGGGCAAGGGTCTGGGCAGCGCCGTCGTGCTCCCCGGAGTGGAGGCGGCCGAGCGCGCCGGGGTGCCCGCCTTCCTGGAGACCTCCGCGCCCCGCAACCTCCCCTTCTACGAGCGGCTCGGCTTCACCGTCACCGCCGACGTCGAGGTGCCCGAAGGACCGCGCACCTGGTGCATGACCCGCAAGCCCGGTGCCTGAGCTAGAAGCTCGCTTTCTTGCTGTCCAATTTCTATTAAAGGTTCCTTTGTTCCCTAAGTCCAACTACTAAACTGGGGGATATTATGAAGGGCCTTGAGCATCTGGATTCTGCCTAATAAAAAACATTTATTTTCATTGCAATGATGTATTTAAATTATTTCTGAATATTTTACTAAAAAGGGAATGTGGGAGGTCAGTGCATTTAAAACATAAAGAAATGAAGAGCTAGTTCAAACCTTGGGAAAATACACTATATCTTAAACTCCATGAAAGAAGGTGAGGCTGCAAACAGCTAATGCACATTGGCAACAGCCCCTGATGCCTATGCCTTATTCATCCCTCAGAAAAGGATTCAAGTAGAGGCTTGATTTGGAGGTTAAAGTTTTGCTATGCTGTATTTTAGAATTCCATACCACATTTGTAGAGGTTTTACTTGCTTTAAAAAACCTCCCACACCTCCCCCTGAACCTGAAACATAAAATGAATGCAATTGTTGTTGTTTTACCAATGCTTAATCAGTGAGGCACCTATCTCAGCGATCTGTCTATTTCGTTCATCCATAGTTGCCTGACTCCCCGTCGTGTAGATAACTACGATACGGGAGGGCTTACCATCTGGCCCCAGTGCTGCAATGATACCGCGAGACCCACGCTCACCGGCTCCAGATTTATCAGCAATAAACCAGCCAGCCGGAAGGGCCGAGCGCAGAAGTGGTCCTGCAACTTTATCCGCCTCCATCCAGTCTATTAATTGTTGCCGGGAAGCTAGAGTAAGTAGTTCGCCAGTTAATAGTTTGCGCAACGTTGTTGCCATTGCTACAGGCATCGTGGTGTCACGCTCGTCGTTTGGTATGGCTTCATTCAGCTCCGGTTCCCAACGATCAAGGCGAGTTACATGATCCCCCATGTTGTGCAAAAAAGCGGTTAGCTCCTTCGGTCCTCCGATCGTTGTCAGAAGTAAGTTGGCCGCAGTGTTATCACTCATGGTTATGGCAGCACTGCATAATTCTCTTACTGTCATGCCATCCGTAAGATGCTTTTCTGTGACTGGTGAGTACTCAACCAAGTCATTCTGAGAATAGTGTATGCGGCGACCGAGTTGCTCTTGCCCGGCGTCAATACGGGATAATACCGCGCCACATAGCAGAACTTTAAAAGTGCTCATCATTGGAAAACGTTCTTCGGGGCGAAAACTCTCAAGGATCTTACCGCTGTTGAGATCCAGTTCGATGTAACCCACTCGTGCACCCAACTGATCTTCAGCATCTTTTACTTTCACCAGCGTTTCTGGGTGAGCAAAAACAGGAAGGCAAAATGCCGCAAAAAAGGGAATAAGGGCGACACGGAAATGTTGAATACTCATACTCTTCCTTTTTCAATATTATTGAAGCATTTATCAGGGTTATTGTCTCATGAGCGGATACATATTTGAATGTATTTAGAAAAATAAACAAATAGGGGTTCCGCGAACTTGTTTATTGCAGCTTATAATGGTTACAAATAAAGCAATAGCATCACAAATTTCACAAATAAAGCATTTTTTTCACTGCATTCTAGTTGTGGTTTGTCCAAACTCATCAATGTATCTTATCATGTCTGGCcagctagccaacaagctcgtcatcgctttgcagaagagcagagaggatatgctcatcgtctaaagaactacccattTTATTATATATTAGTCACGATATCTATAACAAGAAAATATATATATAATAAGTTATCACGTAAGTAGAACATGAAATAACAATATAATTATCGTATGAGTTAAATCTTAAAAGTCACGTAAAAGATAATCATGCGTCATTTTGACTCACGCGGTCGTTATAGTTCAAAATCAGTGACACTTACCGCATTGACAAGCACGCCTCACGGGAGCTCCAAGCGGCGACTGAGATGTCCTAAATGCACAGCGACGGATTCGCGCTATTTAGAAAGAGAGAGCAATATTTCAAGAATGCATGCGTCAATTTTACGCAGACTATCTTTCTAGGGTTAAAAAAGATTTGCGCTTTACTCGACCTAAACTTTAAACACGTCATAGAATCTTCGTTTGACAAAAACCACATTGTGGCCAAGCTGTGTGACGCGACGCGCGCTAAAGAATGGCAAACCAAGTCGCGCGAGGTACCCAGCTTTTGTTCCCTTTAGTGAGGGTTAATTCCGAGCTTGGCGTAATCATGGTCATAGCTGTTTCCTGTGTGAAATTGTTATCCGCTCACAATTCCACACAACATACGAGCCGGAAGCATAAAGTGTAAAGCCTGGGGTGCCTAATGAGTGAGCTAACTCACATTAATTGCGTTGCGCTCACTGCCCGCTTTCCAGTCGGGAAACCTGTCGTGCCAGCTGCATTAATGAATCGGCCAACGCGCGGGGAGAGGCGGTTTGCGTATTGGGCGCTCTTCCGCTTCCTCGCTCACTGACTCGCTGCGCTCGGTCGTTCGGCTGCGGCGAGCGGTATCAGCTCACTCAAAGGCGGTAATACGGTTATCCACAGAATCAGGGGATAACGCAGGAAAGAACATGTGAGCAAAAGGCCAGCAAAAGGCCAGGAACCGTAAAAAGGCCGCGTTGCTGGCGTTTTTCCATAGGCTCCGCCCCCCTGACGAGCATCACAAAAATCGACGCTCAAGTCAGAGGTGGCGAAACCCGACAGGACTATAAAGATACCAGGCGTTTCCCCCTGGAAGCTCCCTCGTGCGCTCTCCTGTTCCGACCCTGCCGCTTACCGGATACCTGTCCGCCTTTCTCCCTTCGGGAAGCGTGGCGCTTTCTCATAGCTCACGCTGTAGGTATCTCAGTTCGGTGTAGGTCGTTCGCTCCAAGCTGGGCTGTGTGCACGAACCCCCCGTTCAGCCCGACCGCTGCGCCTTATCCGGTAACTATCGTCTTGAGTCCAACCCGGTAAGACACGACTTATCGCCACTGGCAGCAGCCACTGGTAACAGGATTAGCAGAGCGAGGTATGTAGGCGGTGCTACAGAGTTCTTGAAGTGGTGGCCTAACTACGGCTACACTAGAAGGACAGTATTTGGTATCTGCGCTCTGCTGAAGCCAGTTACCTTCGGAAAAAGAGTTGGTAGCTCTTGATCCGGCAAACAAACCACCGCTGGTAGCGGTGGTTTTTTTGTTTGCAAGCAGCAGATTACGCGCAGAAAAAAAGGATCTCAAGAAGATCCTTTGATCTTTTCTACGGGGTCTGACGCTCAGTGGAACGAAAACTCACGTTAAGGGATTTTGGTCATGATTAACCCTAGAAAGATAATCATATTGTGACGTACGTTAAAGATAATCATGCGTAAAATTGACGCATGTGTTTTATCGGTCTGTATATCGAGGTTTATTTATTAATTTGAATAGATATTAAGTTTTATTATATTTACACTTACATACTAATAATAAATTCAACAAACAATTTATTTATGTTTATTTATTTATTAAAAAAAAACAAAAACTCAAAATTTCTTCTATAAAGTAACAAAacttttaaacattctctcttttacaaaaataaacttattttgtactttaaaaacagtcatgttgtattataaaataagtaattagcttaacctatacataatagaaacaaattatacttattagtcagtcagaaacaaCTTTGGCACATATCAATATTATGCTCTCGTTAATTAA

pAllin1-PEmax-nuclease

epegRNA U6 Promoter CMV Promoter PEmax-nuclease EF1alpha Promoter Puromycin resistance ITR

CGATGTACGGGCCAGATATACGCGCAATGTCAACGCGTATATCTAAAAAATTCTAGTTGGTTTAACGCGTAACTAGATAGAACCGCGNNNNNNNNNNNNNNNNNNNNNNNNNNNNNNGCACCGACTCGGTGCCACTTTTTCAAGTTGATAACGGACTAGCCTTATTTTAACTTGCTATTTCTAGCTCTAAAACNNNNNNNNNNNNNNNNNNNCGGTGTTTCGTCCTTTCCACAAGATATATAAAGCCAAGAAATCGAAATACTTTCAAGTTACGGTAAGCATATGATAGTCCATTTTAAAACATAATTTTAAAACTGCAAACTACCCAAGAAATTATTACTTTCTACGTCACGTATTTTGTACTAATATCTTTGTGTTTACAGTCAAATTAATTCCAATTATCTCTCTAACAGCCTTGTATCGTATATGCAAATATGAAGGAATCATGGGAAATAGGCCCTCCGCGTTGACATTGATTATTGACTAGTTATTAATAGTAATCAATTACGGGGTCATTAGTTCATAGCCCATATATGGAGTTCCGCGTTACATAACTTACGGTAAATGGCCCGCCTGGCTGACCGCCCAACGACCCCCGCCCATTGACGTCAATAATGACGTATGTTCCCATAGTAACGCCAATAGGGACTTTCCATTGACGTCAATGGGTGGAGTATTTACGGTAAACTGCCCACTTGGCAGTACATCAAGTGTATCATATGCCAAGTACGCCCCCTATTGACGTCAATGACGGTAAATGGCCCGCCTGGCATTATGCCCAGTACATGACCTTATGGGACTTTCCTACTTGGCAGTACATCTACGTATTAGTCATCGCTATTACCATGGTGATGCGGTTTTGGCAGTACATCAATGGGCGTGGATAGCGGTTTGACTCACGGGGATTTCCAAGTCTCCACCCCATTGACGTCAATGGGAGTTTGTTTTGGCACCAAAATCAACGGGACTTTCCAAAATGTCGTAACAACTCCGCCCCATTGACGCAAATGGGCGGTAGGCGTGTACGGTGGGAGGTCTATATAAGCAGAGCTGGTTTAGTGAACCGTCAGATCCGCTAGAGATCCGCGGCCGCTAATACGACTCACTATAGGGAGAGCCGCCACCATGAAACGGACAGCCGACGGAAGCGAGTTCGAGTCACCAAAGAAGAAGCGGAAAGTCGACAAGAAGTACAGCATCGGCCTGGACATCGGCACCAACTCTGTGGGCTGGGCCGTGATCACCGACGAGTACAAGGTGCCCAGCAAGAAATTCAAGGTGCTGGGCAACACCGACCGGCACAGCATCAAGAAGAACCTGATCGGAGCCCTGCTGTTCGACAGCGGCGAAACAGCCGAGGCCACCCGGCTGAAGAGAACCGCCAGAAGAAGATACACCAGACGGAAGAACCGGATCTGCTATCTGCAAGAGATCTTCAGCAACGAGATGGCCAAGGTGGACGACAGCTTCTTCCACAGACTGGAAGAGTCCTTCCTGGTGGAAGAGGATAAGAAGCACGAGCGGCACCCCATCTTCGGCAACATCGTGGACGAGGTGGCCTACCACGAGAAGTACCCCACCATCTACCACCTGAGAAAGAAACTGGTGGACAGCACCGACAAGGCCGACCTGCGGCTGATCTATCTGGCCCTGGCCCACATGATCAAGTTCCGGGGCCACTTCCTGATCGAGGGCGACCTGAACCCCGACAACAGCGACGTGGACAAGCTGTTCATCCAGCTGGTGCAGACCTACAACCAGCTGTTCGAGGAAAACCCCATCAACGCCAGCGGCGTGGACGCCAAGGCCATCCTGTCTGCCAGACTGAGCAAGAGCAGAAAGCTGGAAAATCTGATCGCCCAGCTGCCCGGCGAGAAGAAGAATGGCCTGTTCGGAAACCTGATTGCCCTGAGCCTGGGCCTGACCCCCAACTTCAAGAGCAACTTCGACCTGGCCGAGGATGCCAAACTGCAGCTGAGCAAGGACACCTACGACGACGACCTGGACAACCTGCTGGCCCAGATCGGCGACCAGTACGCCGACCTGTTTCTGGCCGCCAAGAACCTGTCCGACGCCATCCTGCTGAGCGACATCCTGAGAGTGAACACCGAGATCACCAAGGCCCCCCTGAGCGCCTCTATGATCAAGAGATACGACGAGCACCACCAGGACCTGACCCTGCTGAAAGCTCTCGTGCGGCAGCAGCTGCCTGAGAAGTACAAAGAGATTTTCTTCGACCAGAGCAAGAACGGCTACGCCGGCTACATTGACGGCGGAGCCAGCCAGGAAGAGTTCTACAAGTTCATCAAGCCCATCCTGGAAAAGATGGACGGCACCGAGGAACTGCTCGTGAAGCTGAAGAGAGAGGACCTGCTGCGGAAGCAGCGGACCTTCGACAACGGCAGCATCCCCCACCAGATCCACCTGGGAGAGCTGCACGCCATTCTGCGGCGGCAGGAAGATTTTTACCCATTCCTGAAGGACAACCGGGAAAAGATCGAGAAGATCCTGACCTTCCGCATCCCCTACTACGTGGGCCCTCTGGCCAGGGGAAACAGCAGATTCGCCTGGATGACCAGAAAGAGCGAGGAAACCATCACCCCCTGGAACTTCGAGGAAGTGGTGGACAAGGGCGCTTCCGCCCAGAGCTTCATCGAGCGGATGACCAACTTCGATAAGAACCTGCCCAACGAGAAGGTGCTGCCCAAGCACAGCCTGCTGTACGAGTACTTCACCGTGTATAACGAGCTGACCAAAGTGAAATACGTGACCGAGGGAATGAGAAAGCCCGCCTTCCTGAGCGGCGAGCAGAAAAAGGCCATCGTGGACCTGCTGTTCAAGACCAACCGGAAAGTGACCGTGAAGCAGCTGAAAGAGGACTACTTCAAGAAAATCGAGTGCTTCGACTCCGTGGAAATCTCCGGCGTGGAAGATCGGTTCAACGCCTCCCTGGGCACATACCACGATCTGCTGAAAATTATCAAGGACAAGGACTTCCTGGACAATGAGGAAAACGAGGACATTCTGGAAGATATCGTGCTGACCCTGACACTGTTTGAGGACAGAGAGATGATCGAGGAACGGCTGAAAACCTATGCCCACCTGTTCGACGACAAAGTGATGAAGCAGCTGAAGCGGCGGAGATACACCGGCTGGGGCAGGCTGAGCCGGAAGCTGATCAACGGCATCCGGGACAAGCAGTCCGGCAAGACAATCCTGGATTTCCTGAAGTCCGACGGCTTCGCCAACAGAAACTTCATGCAGCTGATCCACGACGACAGCCTGACCTTTAAAGAGGACATCCAGAAAGCCCAGGTGTCCGGCCAGGGCGATAGCCTGCACGAGCACATTGCCAATCTGGCCGGCAGCCCCGCCATTAAGAAGGGCATCCTGCAGACAGTGAAGGTGGTGGACGAGCTCGTGAAAGTGATGGGCCGGCACAAGCCCGAGAACATCGTGATCGAAATGGCCAGAGAGAACCAGACCACCCAGAAGGGACAGAAGAACAGCCGCGAGAGAATGAAGCGGATCGAAGAGGGCATCAAAGAGCTGGGCAGCCAGATCCTGAAAGAACACCCCGTGGAAAACACCCAGCTGCAGAACGAGAAGCTGTACCTGTACTACCTGCAGAATGGGCGGGATATGTACGTGGACCAGGAACTGGACATCAACCGGCTGTCCGACTACGATGTGGACCACATCGTGCCTCAGAGCTTTCTGAAGGACGACTCCATCGACAACAAGGTGCTGACCAGAAGCGACAAGAACCGGGGCAAGAGCGACAACGTGCCCTCCGAAGAGGTCGTGAAGAAGATGAAGAACTACTGGCGGCAGCTGCTGAACGCCAAGCTGATTACCCAGAGAAAGTTCGACAATCTGACCAAGGCCGAGAGAGGCGGCCTGAGCGAACTGGATAAGGCCGGCTTCATCAAGAGACAGCTGGTGGAAACCCGGCAGATCACAAAGCACGTGGCACAGATCCTGGACTCCCGGATGAACACTAAGTACGACGAGAATGACAAGCTGATCCGGGAAGTGAAAGTGATCACCCTGAAGTCCAAGCTGGTGTCCGATTTCCGGAAGGATTTCCAGTTTTACAAAGTGCGCGAGATCAACAACTACCACCACGCCCACGACGCCTACCTGAACGCCGTCGTGGGAACCGCCCTGATCAAAAAGTACCCTAAGCTGGAAAGCGAGTTCGTGTACGGCGACTACAAGGTGTACGACGTGCGGAAGATGATCGCCAAGAGCGAGCAGGAAATCGGCAAGGCTACCGCCAAGTACTTCTTCTACAGCAACATCATGAACTTTTTCAAGACCGAGATTACCCTGGCCAACGGCGAGATCCGGAAGCGGCCTCTGATCGAGACAAACGGCGAAACCGGGGAGATCGTGTGGGATAAGGGCCGGGATTTTGCCACCGTGCGGAAAGTGCTGAGCATGCCCCAAGTGAATATCGTGAAAAAGACCGAGGTGCAGACAGGCGGCTTCAGCAAAGAGTCTATCCTGCCCAAGAGGAACAGCGATAAGCTGATCGCCAGAAAGAAGGACTGGGACCCTAAGAAGTACGGCGGCTTCGACAGCCCCACCGTGGCCTATTCTGTGCTGGTGGTGGCCAAAGTGGAAAAGGGCAAGTCCAAGAAACTGAAGAGTGTGAAAGAGCTGCTGGGGATCACCATCATGGAAAGAAGCAGCTTCGAGAAGAATCCCATCGACTTTCTGGAAGCCAAGGGCTACAAAGAAGTGAAAAAGGACCTGATCATCAAGCTGCCTAAGTACTCCCTGTTCGAGCTGGAAAACGGCCGGAAGAGAATGCTGGCCTCTGCCGGCGAACTGCAGAAGGGAAACGAACTGGCCCTGCCCTCCAAATATGTGAACTTCCTGTACCTGGCCAGCCACTATGAGAAGCTGAAGGGCTCCCCCGAGGATAATGAGCAGAAACAGCTGTTTGTGGAACAGCACAAGCACTACCTGGACGAGATCATCGAGCAGATCAGCGAGTTCTCCAAGAGAGTGATCCTGGCCGACGCTAATCTGGACAAAGTGCTGTCCGCCTACAACAAGCACCGGGATAAGCCCATCAGAGAGCAGGCCGAGAATATCATCCACCTGTTTACCCTGACCAATCTGGGAGCCCCTGCCGCCTTCAAGTACTTTGACACCACCATCGACCGGAAGAGGTACACCAGCACCAAAGAGGTGCTGGACGCCACCCTGATCCACCAGAGCATCACCGGCCTGTACGAGACACGGATCGACCTGTCTCAGCTGGGAGGTGACTCCGGCGGAAGCTCTGGTGGCAGCAAGCGGACCGCCGACGGCTCTGAATTCGAGAGCCCTAAGAAGAAAAGAAAGGTGAGCGGAGGCTCTAGCGGCGGAAGCACCCTGAACATTGAAGACGAGTATAGACTGCATGAAACAAGCAAGGAACCCGACGTGTCCCTGGGCTCCACCTGGCTGTCCGACTTTCCCCAGGCCTGGGCCGAGACAGGAGGAATGGGCCTGGCCGTGCGGCAGGCACCCCTGATCATCCCTCTGAAGGCCACCTCTACACCCGTGAGCATCAAGCAGTACCCTATGTCTCAGGAGGCCAGACTGGGCATCAAGCCTCACATCCAGAGGCTGCTGGACCAGGGCATCCTGGTGCCATGCCAGAGCCCCTGGAACACACCACTGCTGCCCGTGAAGAAGCCAGGCACCAATGACTATAGACCCGTGCAGGATCTGAGAGAGGTGAACAAGAGGGTGGAGGATATCCACCCCACCGTGCCCAACCCTTACAATCTGCTGTCCGGCCTGCCCCCTTCTCACCAGTGGTATACAGTGCTGGACCTGAAGGATGCCTTCTTTTGTCTGAGACTGCACCCTACCAGCCAGCCACTGTTCGCCTTTGAGTGGAGGGACCCTGAGATGGGCATCTCTGGCCAGCTGACCTGGACACGCCTGCCTCAGGGCTTCAAGAATAGCCCAACACTGTTTAACGAGGCCCTGCACCGCGACCTGGCAGATTTCCGGATCCAGCACCCAGATCTGATCCTGCTGCAGTACGTGGACGATCTGCTGCTGGCCGCCACCAGCGAGCTGGATTGCCAGCAGGGAACACGCGCCCTGCTGCAGACCCTGGGAAACCTGGGATATAGGGCATCCGCCAAGAAGGCCCAGATCTGTCAGAAGCAGGTGAAGTACCTGGGCTATCTGCTGAAGGAGGGCCAGAGATGGCTGACAGAGGCCAGGAAGGAGACAGTGATGGGCCAGCCAACACCCAAGACCCCAAGACAGCTGAGGGAGTTCCTGGGCAAAGCAGGATTTTGCAGGCTGTTCATCCCAGGATTCGCAGAGATGGCAGCACCTCTGTACCCACTGACCAAGCCGGGCACCCTGTTTAATTGGGGCCCTGACCAGCAGAAGGCCTATCAGGAGATCAAGCAGGCCCTGCTGACAGCACCAGCCCTGGGCCTGCCAGACCTGACCAAGCCTTTCGAGCTGTTTGTGGATGAGAAGCAGGGCTACGCCAAGGGCGTGCTGACCCAGAAGCTGGGACCATGGAGACGGCCCGTGGCCTATCTGTCCAAGAAGCTGGACCCAGTGGCAGCAGGATGGCCACCATGCCTGAGGATGGTGGCAGCAATCGCCGTGCTGACAAAGGATGCCGGCAAGCTGACCATGGGACAGCCACTGGTCATCCTGGCACCACACGCAGTGGAGGCCCTGGTGAAGCAGCCTCCAGATCGCTGGCTGTCTAACGCCCGGATGACACACTACCAGGCCCTGCTGCTGGACACCGATCGCGTGCAGTTTGGCCCTGTGGTGGCCCTGAATCCAGCCACCCTGCTGCCTCTGCCAGAGGAGGGCCTGCAGCACAACTGTCTGGACATCCTGGCAGAGGCACACGGAACAAGGCCAGACCTGACCGATCAGCCCCTGCCTGACGCCGATCACACATGGTATACCGATGGAAGCTCCCTGCTGCAGGAGGGCCAGAGGAAGGCAGGAGCAGCAGTGACCACAGAGACAGAAGTGATCTGGGCCAAGGCCCTGCCAGCAGGCACATCCGCCCAGCGGGCCGAGCTGATCGCCCTGACCCAGGCCCTGAAGATGGCCGAGGGCAAGAAGCTGAACGTGTACACAGACTCCAGATATGCCTTCGCCACCGCACACATCCACGGAGAGATCTACAGGCGCCGGGGCTGGCTGACCTCTGAGGGCAAGGAGATCAAGAACAAGGATGAGATCCTGGCCCTGCTGAAGGCCCTGTTTCTGCCCAAGCGGCTGAGCATCATCCACTGTCCTGGACACCAGAAGGGACACTCCGCCGAGGCAAGGGGCAATCGGATGGCCGACCAGGCCGCCAGAAAGGCTGCTATTACTGAAACTCCCGACACTTCCACTCTGCTGATTGAAAACTCCTCCCCTTCTGGCGGCTCAAAAAGAACCGCCGACGGCAGCGAATTCGAGTCTCCCAAGAAGAAGAGGAAAGTCGGCTCTGGCCCTGCCGCTAAGAGAGTGAAGCTGGACTAACCGGTCATCATCACCATCACCATTGAGTTTTACCCCTACGACGTGCCCGACTACGCCTAATAACTCGAGCATGCATCTAGAGGGCCCTATTCTATAGTGTCACCTAAATGCTAGAGCTCGCTGATCAGCCTCGACTGTGCCTTCTAGTTGCCAGCCATCTGTTGTTTGCCCCTCCCCCGTGCCTTCCTTGACCCTGGAAGGTGCCACTCCCACTGTCCTTTCCTAATAAAATGAGGAAATTGCATCGCATTGTCTGAGTAGGTGTCATTCTATTCTGGGGGGTGGGGTGGGGCAGGACAGCAAGGGGGAGGATTGGGAAGACAATAGCAGGCATGCTGGGGAGGATCTGCGATCGCTCCGGTGCCCGTCAGTGGGCAGAGCGCACATCGCCCACAGTCCCCGAGAAGTTGGGGGGAGGGGTCGGCAATTGAACGGGTGCCTAGAGAAGGTGGCGCGGGGTAAACTGGGAAAGTGATGTCGTGTACTGGCTCCGCCTTTTTCCCGAGGGTGGGGGAGAACCGTATATAAGTGCAGTAGTCGCCGTGAACGTTCTTTTTCGCAACGGGTTTGCCGCCAGAACACAGCTGAAGCTTCGAGGGGCTCGCATCTCTCCTTCACGCGCCCGCCGCCCTACCTGAGGCCGCCATCCACGCCGGTTGAGTCGCGTTCTGCCGCCTCCCGCCTGTGGTGCCTCCTGAACTGCGTCCGCCGTCTAGGTAAGTTTAAAGCTCAGGTCGAGACCGGGCCTTTGTCCGGCGCTCCCTTGGAGCCTACCTAGACTCAGCCGGCTCTCCACGCTTTGCCTGACCCTGCTTGCTCAACTCTACGTCTTTGTTTCGTTTTCTGTTCTGCGCCGTTACAGATCCAAGCTGTGACCGGCGCCTACATGACCGAGTACAAGCCCACGGTGCGCCTCGCCACCCGCGACGACGTCCCCAGGGCCGTACGCACCCTCGCCGCCGCGTTCGCCGACTACCCCGCCACGCGCCACACCGTCGATCCAGACCGCCACATCGAGCGGGTCACCGAGCTGCAAGAACTCTTCCTCACGCGCGTCGGGCTCGACATCGGCAAGGTGTGGGTCGCGGACGACGGCGCCGCGGTGGCGGTCTGGACCACGCCGGAGAGCGTCGAAGCGGGGGCGGTGTTCGCCGAGATCGGCCCGCGCATGGCCGAGTTGAGCGGTTCCCGGCTGGCCGCGCAGCAACAGATGGAAGGTCTCCTGGCGCCGCACCGGCCCAAGGAGCCCGCGTGGTTCCTGGCCACCGTCGGCGTCTCGCCCGACCACCAGGGCAAGGGTCTGGGCAGCGCCGTCGTGCTCCCCGGAGTGGAGGCGGCCGAGCGCGCCGGGGTGCCCGCCTTCCTGGAGACCTCCGCGCCCCGCAACCTCCCCTTCTACGAGCGGCTCGGCTTCACCGTCACCGCCGACGTCGAGGTGCCCGAAGGACCGCGCACCTGGTGCATGACCCGCAAGCCCGGTGCCTGAGCTAGAAGCTCGCTTTCTTGCTGTCCAATTTCTATTAAAGGTTCCTTTGTTCCCTAAGTCCAACTACTAAACTGGGGGATATTATGAAGGGCCTTGAGCATCTGGATTCTGCCTAATAAAAAACATTTATTTTCATTGCAATGATGTATTTAAATTATTTCTGAATATTTTACTAAAAAGGGAATGTGGGAGGTCAGTGCATTTAAAACATAAAGAAATGAAGAGCTAGTTCAAACCTTGGGAAAATACACTATATCTTAAACTCCATGAAAGAAGGTGAGGCTGCAAACAGCTAATGCACATTGGCAACAGCCCCTGATGCCTATGCCTTATTCATCCCTCAGAAAAGGATTCAAGTAGAGGCTTGATTTGGAGGTTAAAGTTTTGCTATGCTGTATTTTAGAATTCCATACCACATTTGTAGAGGTTTTACTTGCTTTAAAAAACCTCCCACACCTCCCCCTGAACCTGAAACATAAAATGAATGCAATTGTTGTTGTTTTACCAATGCTTAATCAGTGAGGCACCTATCTCAGCGATCTGTCTATTTCGTTCATCCATAGTTGCCTGACTCCCCGTCGTGTAGATAACTACGATACGGGAGGGCTTACCATCTGGCCCCAGTGCTGCAATGATACCGCGAGACCCACGCTCACCGGCTCCAGATTTATCAGCAATAAACCAGCCAGCCGGAAGGGCCGAGCGCAGAAGTGGTCCTGCAACTTTATCCGCCTCCATCCAGTCTATTAATTGTTGCCGGGAAGCTAGAGTAAGTAGTTCGCCAGTTAATAGTTTGCGCAACGTTGTTGCCATTGCTACAGGCATCGTGGTGTCACGCTCGTCGTTTGGTATGGCTTCATTCAGCTCCGGTTCCCAACGATCAAGGCGAGTTACATGATCCCCCATGTTGTGCAAAAAAGCGGTTAGCTCCTTCGGTCCTCCGATCGTTGTCAGAAGTAAGTTGGCCGCAGTGTTATCACTCATGGTTATGGCAGCACTGCATAATTCTCTTACTGTCATGCCATCCGTAAGATGCTTTTCTGTGACTGGTGAGTACTCAACCAAGTCATTCTGAGAATAGTGTATGCGGCGACCGAGTTGCTCTTGCCCGGCGTCAATACGGGATAATACCGCGCCACATAGCAGAACTTTAAAAGTGCTCATCATTGGAAAACGTTCTTCGGGGCGAAAACTCTCAAGGATCTTACCGCTGTTGAGATCCAGTTCGATGTAACCCACTCGTGCACCCAACTGATCTTCAGCATCTTTTACTTTCACCAGCGTTTCTGGGTGAGCAAAAACAGGAAGGCAAAATGCCGCAAAAAAGGGAATAAGGGCGACACGGAAATGTTGAATACTCATACTCTTCCTTTTTCAATATTATTGAAGCATTTATCAGGGTTATTGTCTCATGAGCGGATACATATTTGAATGTATTTAGAAAAATAAACAAATAGGGGTTCCGCGAACTTGTTTATTGCAGCTTATAATGGTTACAAATAAAGCAATAGCATCACAAATTTCACAAATAAAGCATTTTTTTCACTGCATTCTAGTTGTGGTTTGTCCAAACTCATCAATGTATCTTATCATGTCTGGCcagctagccaacaagctcgtcatcgctttgcagaagagcagagaggatatgctcatcgtctaaagaactacccattTTATTATATATTAGTCACGATATCTATAACAAGAAAATATATATATAATAAGTTATCACGTAAGTAGAACATGAAATAACAATATAATTATCGTATGAGTTAAATCTTAAAAGTCACGTAAAAGATAATCATGCGTCATTTTGACTCACGCGGTCGTTATAGTTCAAAATCAGTGACACTTACCGCATTGACAAGCACGCCTCACGGGAGCTCCAAGCGGCGACTGAGATGTCCTAAATGCACAGCGACGGATTCGCGCTATTTAGAAAGAGAGAGCAATATTTCAAGAATGCATGCGTCAATTTTACGCAGACTATCTTTCTAGGGTTAAAAAAGATTTGCGCTTTACTCGACCTAAACTTTAAACACGTCATAGAATCTTCGTTTGACAAAAACCACATTGTGGCCAAGCTGTGTGACGCGACGCGCGCTAAAGAATGGCAAACCAAGTCGCGCGAGGTACCCAGCTTTTGTTCCCTTTAGTGAGGGTTAATTCCGAGCTTGGCGTAATCATGGTCATAGCTGTTTCCTGTGTGAAATTGTTATCCGCTCACAATTCCACACAACATACGAGCCGGAAGCATAAAGTGTAAAGCCTGGGGTGCCTAATGAGTGAGCTAACTCACATTAATTGCGTTGCGCTCACTGCCCGCTTTCCAGTCGGGAAACCTGTCGTGCCAGCTGCATTAATGAATCGGCCAACGCGCGGGGAGAGGCGGTTTGCGTATTGGGCGCTCTTCCGCTTCCTCGCTCACTGACTCGCTGCGCTCGGTCGTTCGGCTGCGGCGAGCGGTATCAGCTCACTCAAAGGCGGTAATACGGTTATCCACAGAATCAGGGGATAACGCAGGAAAGAACATGTGAGCAAAAGGCCAGCAAAAGGCCAGGAACCGTAAAAAGGCCGCGTTGCTGGCGTTTTTCCATAGGCTCCGCCCCCCTGACGAGCATCACAAAAATCGACGCTCAAGTCAGAGGTGGCGAAACCCGACAGGACTATAAAGATACCAGGCGTTTCCCCCTGGAAGCTCCCTCGTGCGCTCTCCTGTTCCGACCCTGCCGCTTACCGGATACCTGTCCGCCTTTCTCCCTTCGGGAAGCGTGGCGCTTTCTCATAGCTCACGCTGTAGGTATCTCAGTTCGGTGTAGGTCGTTCGCTCCAAGCTGGGCTGTGTGCACGAACCCCCCGTTCAGCCCGACCGCTGCGCCTTATCCGGTAACTATCGTCTTGAGTCCAACCCGGTAAGACACGACTTATCGCCACTGGCAGCAGCCACTGGTAACAGGATTAGCAGAGCGAGGTATGTAGGCGGTGCTACAGAGTTCTTGAAGTGGTGGCCTAACTACGGCTACACTAGAAGGACAGTATTTGGTATCTGCGCTCTGCTGAAGCCAGTTACCTTCGGAAAAAGAGTTGGTAGCTCTTGATCCGGCAAACAAACCACCGCTGGTAGCGGTGGTTTTTTTGTTTGCAAGCAGCAGATTACGCGCAGAAAAAAAGGATCTCAAGAAGATCCTTTGATCTTTTCTACGGGGTCTGACGCTCAGTGGAACGAAAACTCACGTTAAGGGATTTTGGTCATGATTAACCCTAGAAAGATAATCATATTGTGACGTACGTTAAAGATAATCATGCGTAAAATTGACGCATGTGTTTTATCGGTCTGTATATCGAGGTTTATTTATTAATTTGAATAGATATTAAGTTTTATTATATTTACACTTACATACTAATAATAAATTCAACAAACAATTTATTTATGTTTATTTATTTATTAAAAAAAAACAAAAACTCAAAATTTCTTCTATAAAGTAACAAAacttttaaacattctctcttttacaaaaataaacttattttgtactttaaaaacagtcatgttgtattataaaataagtaattagcttaacctatacataatagaaacaaattatacttattagtcagtcagaaacaaCTTTGGCACATATCAATATTATGCTCTCGTTAATTAA
