# Supplementary material for: TAPE-seq is a cell-based method for predicting genome-wide off-target effects of prime editor
Source: Nat Commun. 2022 Dec 29;13:7975. doi: 10.1038/s41467-022-35743-y (PMC9800413; doi:10.1038/s41467-022-35743-y)
Supplement: Supplementary file 13 — Reporting Summary [file 41467_2022_35743_MOESM13_ESM.pdf]

nature portfolio

Corresponding author(s): Jungjoon K. Lee

Last updated by author(s): Dec 12, 2022

Reporting Summary

Nature Portfolio wishes to improve the reproducibility of the work that we publish. This form provides structure for consistency and transparency in reporting. For further information on Nature Portfolio policies, see our Editorial Policies and the Editorial Policy Checklist.

Statistics

For all statistical analyses, confirm that the following items are present in the figure legend, table legend, main text, or Methods section.

n/a	Confirmed	
[ ]	[x]	The exact sample size (*n*) for each experimental group/condition, given as a discrete number and unit of measurement
[ ]	[x]	A statement on whether measurements were taken from distinct samples or whether the same sample was measured repeatedly
[ ]	[x]	The statistical test(s) used AND whether they are one- or two-sided<br>*Only common tests should be described solely by name; describe more complex techniques in the Methods section.*
[x]	[ ]	A description of all covariates tested
[x]	[ ]	A description of any assumptions or corrections, such as tests of normality and adjustment for multiple comparisons
[ ]	[x]	A full description of the statistical parameters including central tendency (e.g. means) or other basic estimates (e.g. regression coefficient) AND variation (e.g. standard deviation) or associated estimates of uncertainty (e.g. confidence intervals)
[ ]	[x]	For null hypothesis testing, the test statistic (e.g. *F*, *t*, *r*) with confidence intervals, effect sizes, degrees of freedom and *P* value noted<br>*Give P values as exact values whenever suitable.*
[x]	[ ]	For Bayesian analysis, information on the choice of priors and Markov chain Monte Carlo settings
[x]	[ ]	For hierarchical and complex designs, identification of the appropriate level for tests and full reporting of outcomes
[x]	[ ]	Estimates of effect sizes (e.g. Cohen's *d*, Pearson's *r*), indicating how they were calculated

Our web collection on statistics for biologists contains articles on many of the points above.

Software and code

Policy information about availability of computer code

Data collection	Targeted deep sequencing was performed using Mi-seq system (Illumina). TAPE-seq was perfomed using Mi-seq (Illumina) and Hi-seq systems (Illumina). Genome-wide off-target prediction was collected using web version Cas-offinder (https://github.com/snugel/cas-offinder).
Data analysis	Indel frequencies from targeted deep sequencing data were calculated with web version Cas Analyzer (http://www.rgenome.net/cas-analyzer/#!). BBDuk (v.38.87), BWA (v.0.7.17-r1188), picard (v.2.20.4), Attune NxT software v4.2.0, samtools (v.1.11), Adobe Illustration CC2018, Microsoft Excel 365, and GraphPad Prism 9 were used for data analysis and visualization.

For manuscripts utilizing custom algorithms or software that are central to the research but not yet described in published literature, software must be made available to editors and reviewers. We strongly encourage code deposition in a community repository (e.g. GitHub). See the Nature Portfolio guidelines for submitting code & software for further information.

Data

Policy information about availability of data

All manuscripts must include a data availability statement. This statement should provide the following information, where applicable:

- Accession codes, unique identifiers, or web links for publicly available datasets
- A description of any restrictions on data availability
- For clinical datasets or third party data, please ensure that the statement adheres to our policy

Deep sequencing data that support the findings of this study have been deposited in NCBI Bioproject(https://www.ncbi.nlm.nih.gov/bioproject/) with the accession codes PRJNA802977.

Human research participants

Policy information about studies involving human research participants and Sex and Gender in Research.

Reporting on sex and gender	Not applicable
Population characteristics	Not applicable
Recruitment	Not applicable
Ethics oversight	Not applicable

Note that full information on the approval of the study protocol must also be provided in the manuscript.

Field-specific reporting

Please select the one below that is the best fit for your research. If you are not sure, read the appropriate sections before making your selection.

[x] Life sciences [ ] Behavioural & social sciences [ ] Ecological, evolutionary & environmental sciences

For a reference copy of the document with all sections, see nature.com/documents/nr-reporting-summary-flat.pdf

Life sciences study design

All studies must disclose on these points even when the disclosure is negative.

Sample size	10 sample sites, which were studied in the previous nDigenome-seq paper, were analyzed.
Data exclusions	No data were excluded from the analyses.
Replication	All experiments were performed from individual biological replicates (n=3). Transfection of every construct was carried out in triplicate. All attempts at replication were successful.
Randomization	Randomization was not relevant for this study as cultured cells were grown and analyzed under identical conditions.
Blinding	Blinding was not relevant for this study as cultured cells were grown and analyzed under identical conditions.

Reporting for specific materials, systems and methods

We require information from authors about some types of materials, experimental systems and methods used in many studies. Here, indicate whether each material, system or method listed is relevant to your study. If you are not sure if a list item applies to your research, read the appropriate section before selecting a response.

Materials & experimental systems

n/a	Involved in the study	
[x]	[ ]	Antibodies
[ ]	[x]	Eukaryotic cell lines
[x]	[ ]	Palaeontology and archaeology
[x]	[ ]	Animals and other organisms
[x]	[ ]	Clinical data
[x]	[ ]	Dual use research of concern

Methods

n/a	Involved in the study	
[x]	[ ]	ChIP-seq
[x]	[ ]	Flow cytometry
[x]	[ ]	MRI-based neuroimaging

Eukaryotic cell lines

Policy information about cell lines and Sex and Gender in Research

Cell line source(s)	HEK293T (ATCC, CRL-11268), HeLa (ATCC CCL-2), and K562 (Sigma 89121407)
Authentication	None of the cell lines used were authenticated.
Mycoplasma contamination	Cell lines were not tested for mycoplasma contamination.
Commonly misidentified lines (See ICLAC register)	No commonly misidentified lines were used.
